# Supplementary material for: Robust inference of bi-directional causal relationships in presence of correlated pleiotropy with GWAS summary data
Source: PLoS Genet. 2022 May 16;18(5):e1010205. doi: 10.1371/journal.pgen.1010205 (PMC9135345; doi:10.1371/journal.pgen.1010205)
Supplement: S1 Text — (PDF) [file pgen.1010205.s001.pdf]

# Supplementary to "Robust Inference of Bi-Directional Causal Relationships in Presence of Correlated Pleiotropy with GWAS Summary Data"

Haoran Xue[1] and Wei Pan[1, 2]

[1]Division of Biostatistics, School of Public Health, University of Minnesota, Minneapolis, Minnesota 55455.
[2]Corresponding author. Email: panxx014@umn.edu. Phone: 612-624-4655. Fax: 612-626-0660.

## Contents

**S1 Full Simulation Results** **2**

**S2 Full Real Data Results** **42**
S2.1 48 Risk Factor-Disease Pairs . . . . . . . . . . . . . . . . . . . . . . . . . . 42
S2.2 Pairs of 4 Diseases . . . . . . . . . . . . . . . . . . . . . . . . . . . . . . . 50
S2.3 Links to GWAS Summary Datasets . . . . . . . . . . . . . . . . . . . . . . . 51

**S3 Theoretical Results** **52**
S3.1 Proof of Theorem 1 . . . . . . . . . . . . . . . . . . . . . . . . . . . . . . . 52
S3.2 Proof of Theorem 2 . . . . . . . . . . . . . . . . . . . . . . . . . . . . . . . 55
S3.3 MR-cML with Data Perturbation . . . . . . . . . . . . . . . . . . . . . . . . 57

# S1 Full Simulation Results

S1 Fig: When both $X$ and $Y$ are continuous, $\theta_{XY} = 0$ and $\xi = 0$, the proportions of significant simulation results obtained by the methods for direction $X \to Y$ (left column) and $Y \to X$ (right column). The first row shows results for four main methods: MR-cML-DP-S, CD-cML-DP-S, CD-Ratio-S, and CD-Egger-S; the second row shows results for four methods without screening: MR-cML-DP, CD-cML-DP, CD-Ratio, and CD-Egger; the third row shows results for other five methods.

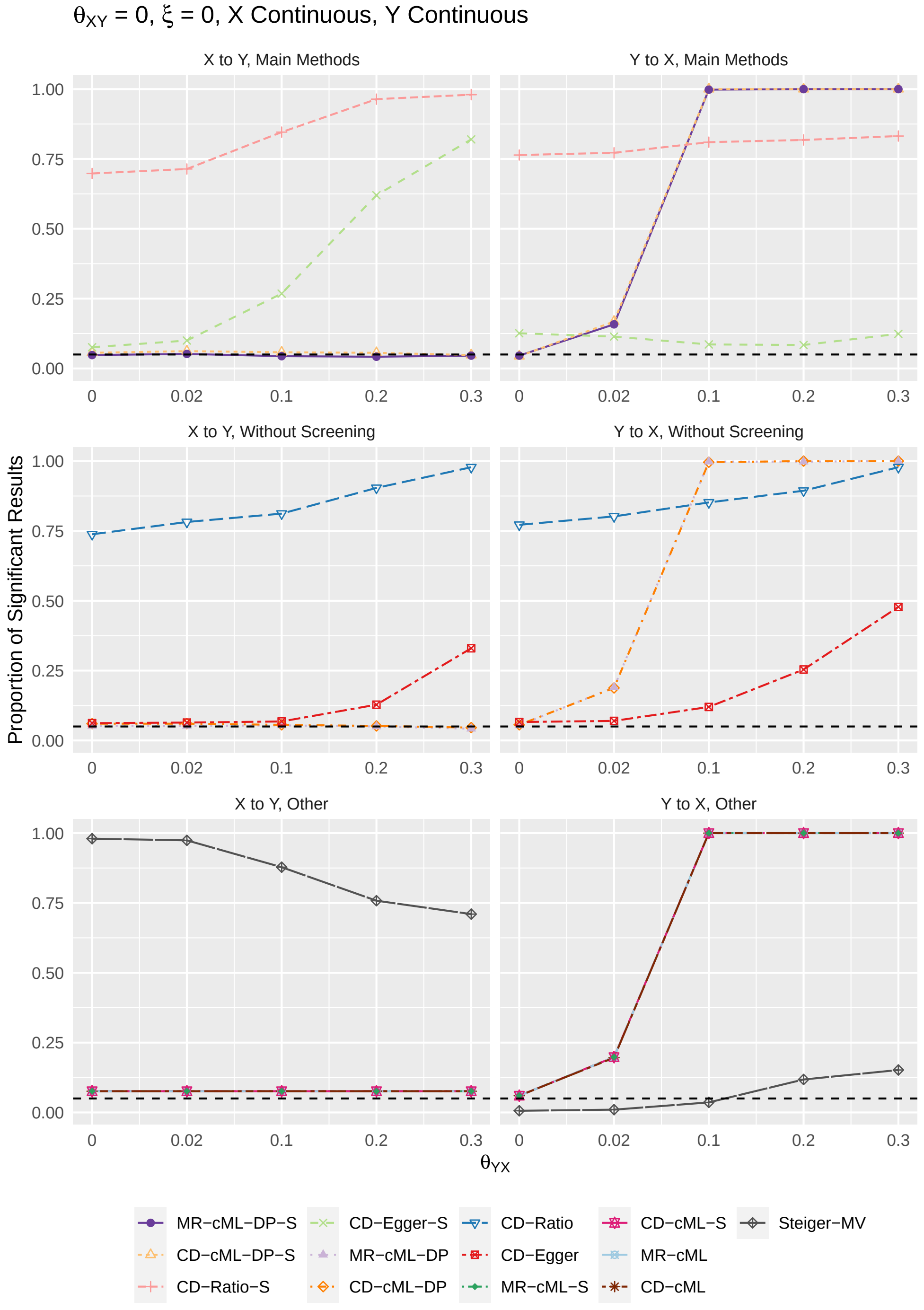

S2 Fig: When both $X$ and $Y$ are continuous, $\theta_{XY} = 0$ and $\xi \sim$ Unif(-0.2,0.2), the proportions of significant simulation results obtained by the methods for direction $X \to Y$ (left column) and $Y \to X$ (right column). The first row shows results for four main methods: MR-cML-DP-S, CD-cML-DP-S, CD-Ratio-S, and CD-Egger-S; the second row shows results for four methods without screening: MR-cML-DP, CD-cML-DP, CD-Ratio, and CD-Egger; the third row shows results for other five methods.

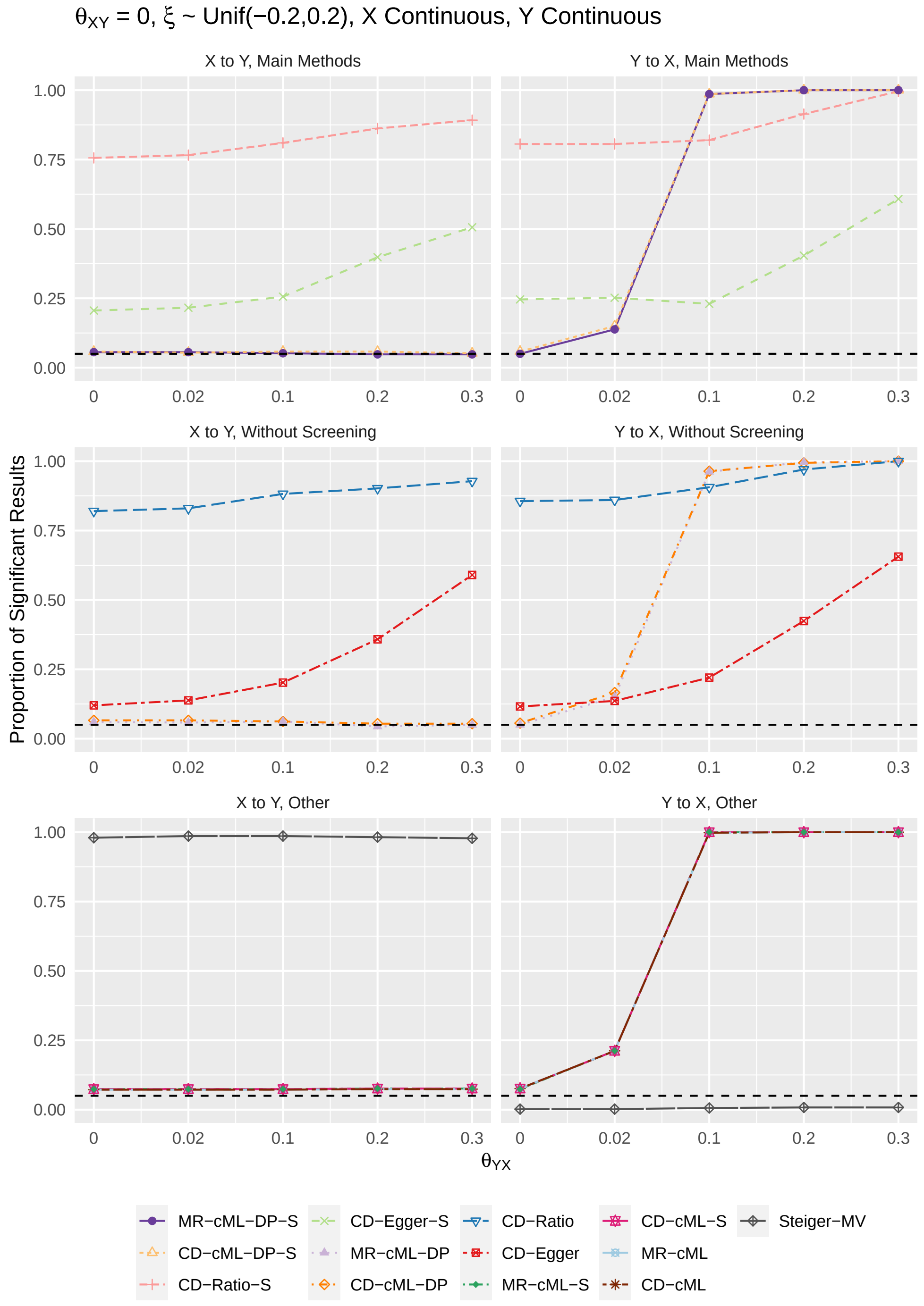

S3 Fig: When both $X$ and $Y$ are continuous, $\theta_{XY} = 0.02$ and $\xi = 0$, the proportions of significant simulation results obtained by the methods for direction $X \to Y$ (left column) and $Y \to X$ (right column). The first row shows results for four main methods: MR-cML-DP-S, CD-cML-DP-S, CD-Ratio-S, and CD-Egger-S; the second row shows results for four methods without screening: MR-cML-DP, CD-cML-DP, CD-Ratio, and CD-Egger; the third row shows results for other five methods.

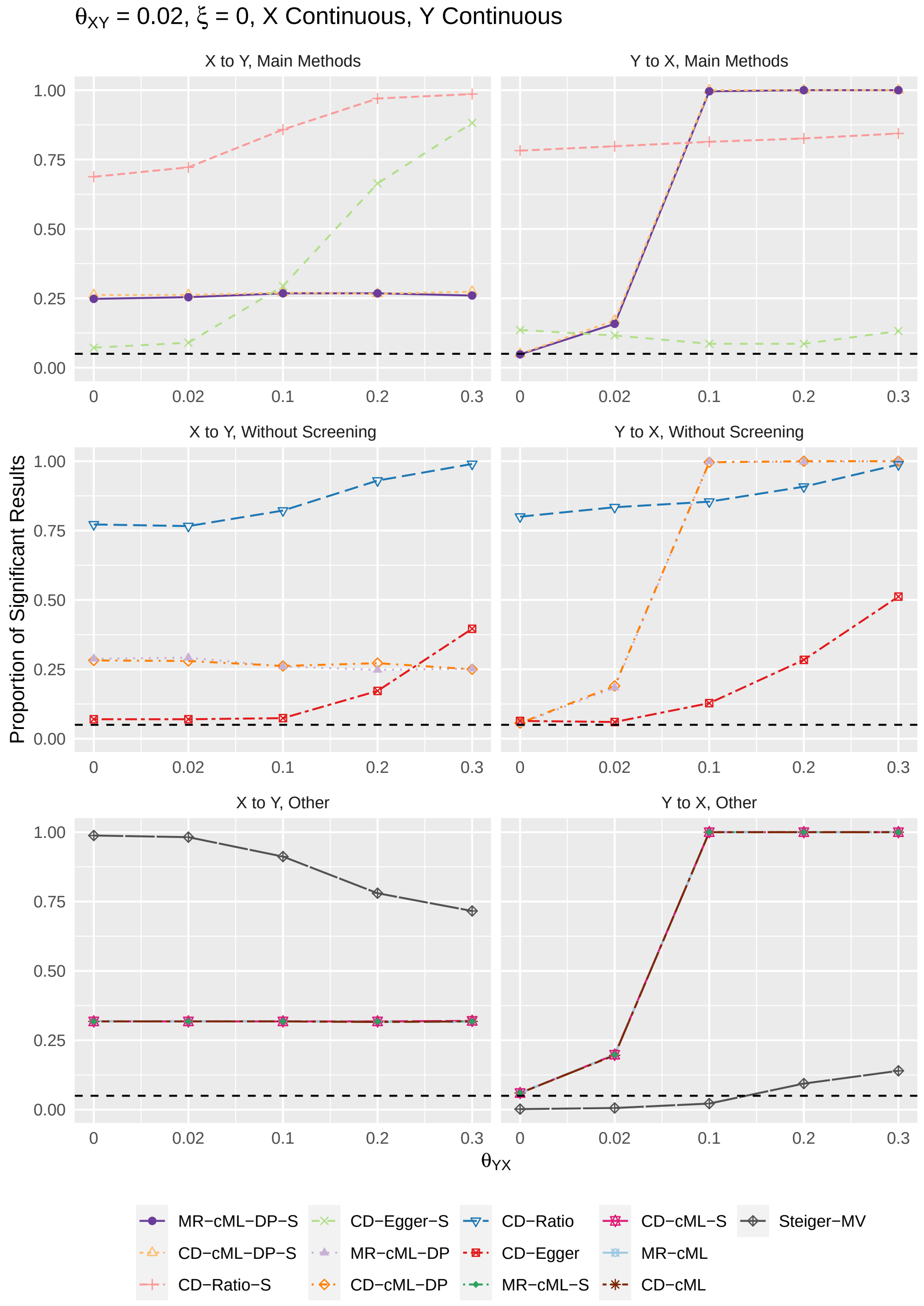

S4 Fig: When both $X$ and $Y$ are continuous, $\theta_{XY} = 0.02$ and $\xi \sim$ Unif(-0.2,0.2), the proportions of significant simulation results obtained by the methods for direction $X \to Y$ (left column) and $Y \to X$ (right column). The first row shows results for four main methods: MR-cML-DP-S, CD-cML-DP-S, CD-Ratio-S, and CD-Egger-S; the second row shows results for four methods without screening: MR-cML-DP, CD-cML-DP, CD-Ratio, and CD-Egger; the third row shows results for other five methods.

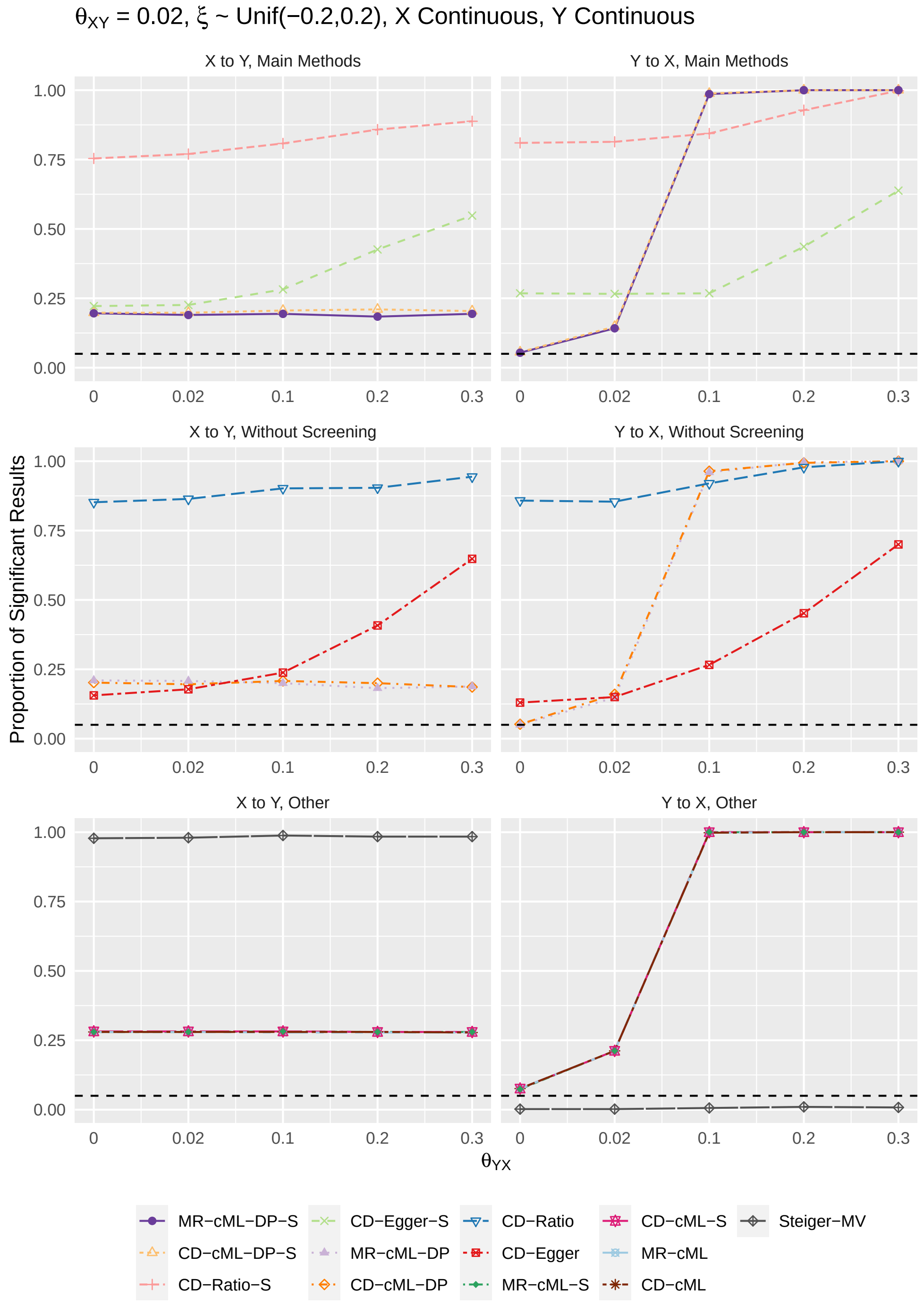

S5 Fig: When both $X$ and $Y$ are continuous, $\theta_{XY} = 0.1$ and $\xi = 0$, the proportions of significant simulation results obtained by the methods for direction $X \to Y$ (left column) and $Y \to X$ (right column). The first row shows results for four main methods: MR-cML-DP-S, CD-cML-DP-S, CD-Ratio-S, and CD-Egger-S; the second row shows results for four methods without screening: MR-cML-DP, CD-cML-DP, CD-Ratio, and CD-Egger; the third row shows results for other five methods.

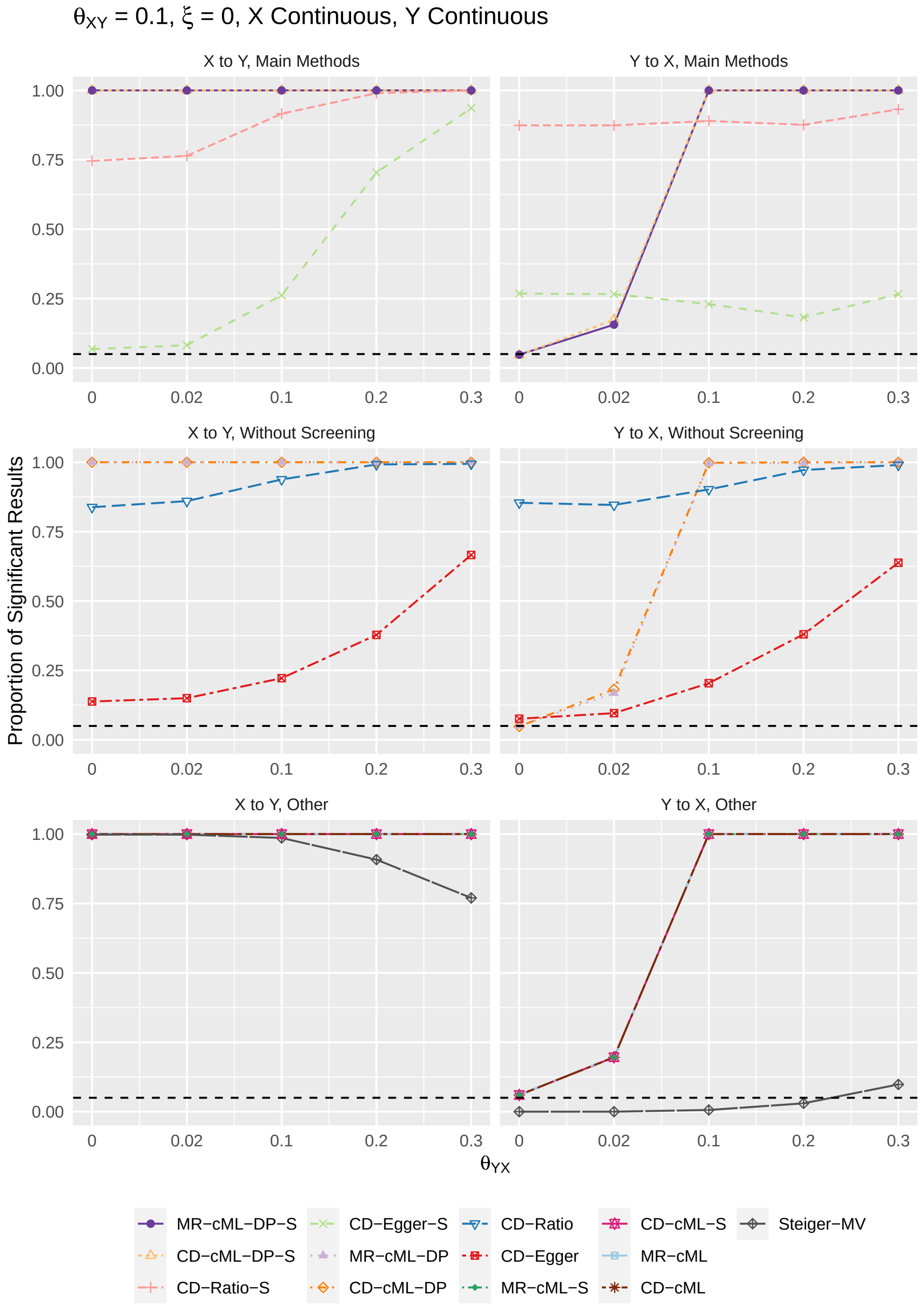

S6 Fig: When both $X$ and $Y$ are continuous, $\theta_{XY} = 0.1$ and $\xi \sim$ Unif(-0.2,0.2), the proportions of significant simulation results obtained by the methods for direction $X \to Y$ (left column) and $Y \to X$ (right column). The first row shows results for four main methods: MR-cML-DP-S, CD-cML-DP-S, CD-Ratio-S, and CD-Egger-S; the second row shows results for four methods without screening: MR-cML-DP, CD-cML-DP, CD-Ratio, and CD-Egger; the third row shows results for other five methods.

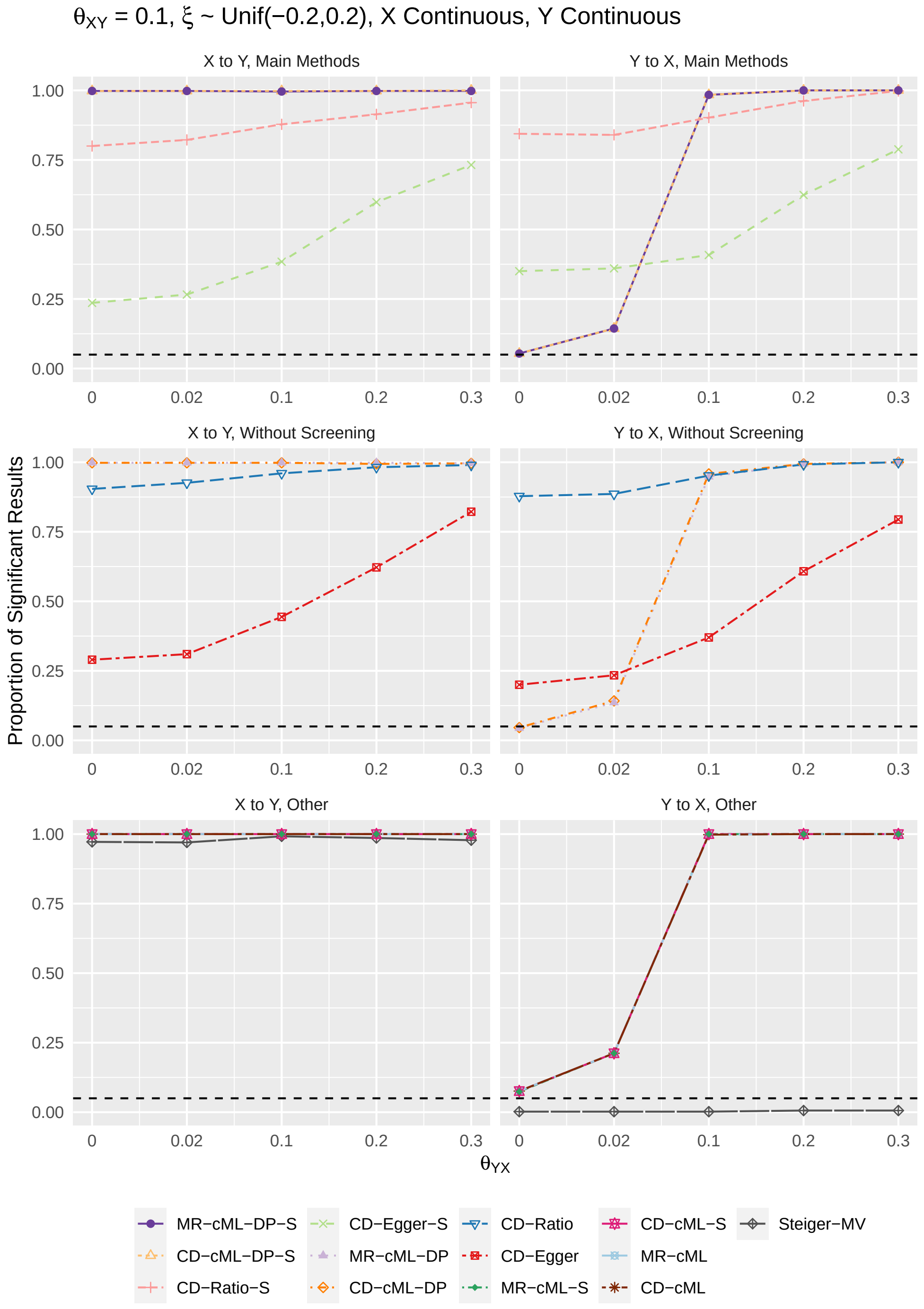

S7 Fig: When both $X$ and $Y$ are continuous, $\theta_{XY} = 0.2$ and $\xi = 0$, the proportions of significant simulation results obtained by the methods for direction $X \to Y$ (left column) and $Y \to X$ (right column). The first row shows results for four main methods: MR-cML-DP-S, CD-cML-DP-S, CD-Ratio-S, and CD-Egger-S; the second row shows results for four methods without screening: MR-cML-DP, CD-cML-DP, CD-Ratio, and CD-Egger; the third row shows results for other five methods.

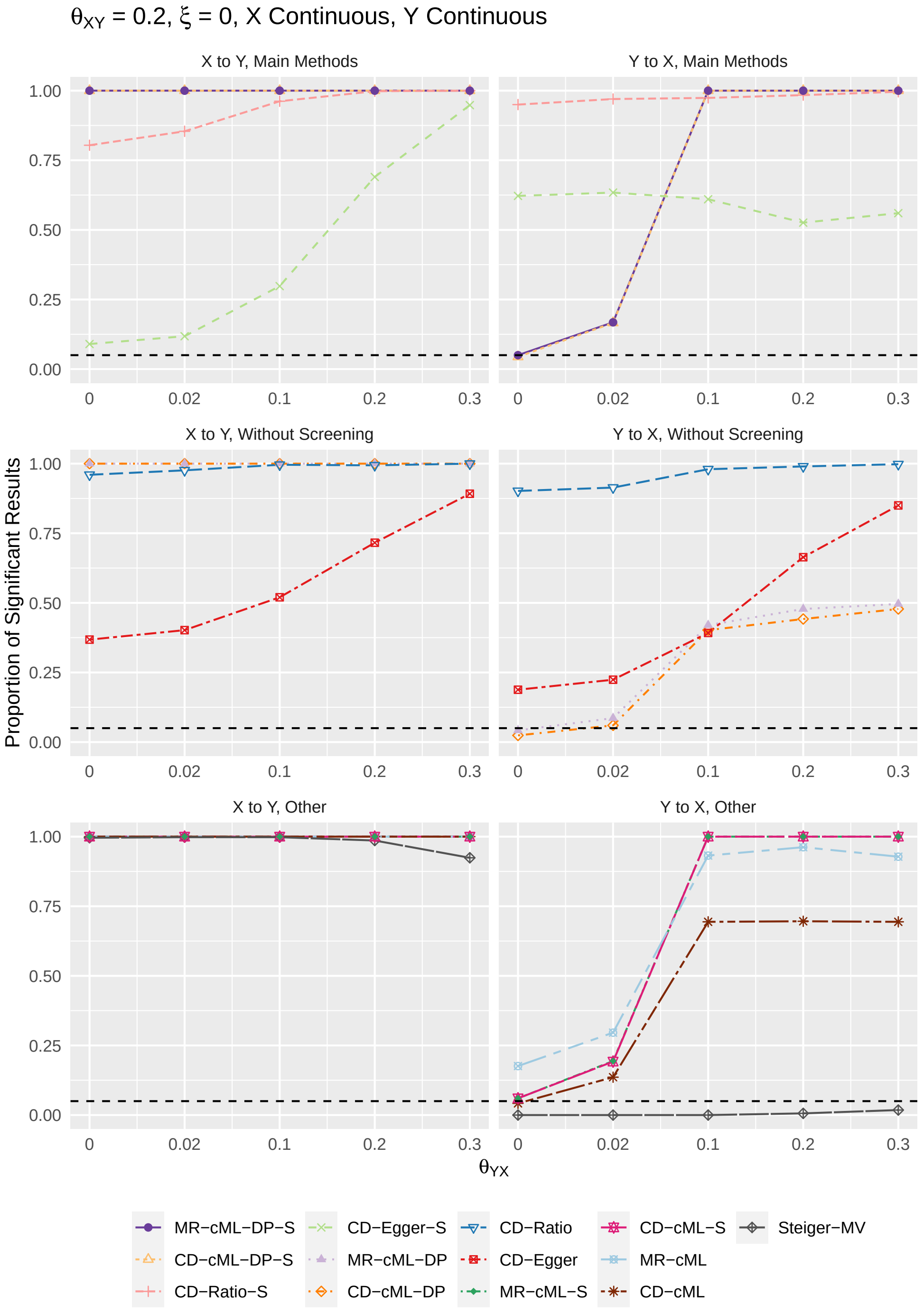

S8 Fig: When both $X$ and $Y$ are continuous, $\theta_{XY} = 0.2$ and $\xi \sim$ Unif(-0.2,0.2), the proportions of significant simulation results obtained by the methods for direction $X \rightarrow Y$ (left column) and $Y \rightarrow X$ (right column). The first row shows results for four main methods: MR-cML-DP-S, CD-cML-DP-S, CD-Ratio-S, and CD-Egger-S; the second row shows results for four methods without screening: MR-cML-DP, CD-cML-DP, CD-Ratio, and CD-Egger; the third row shows results for other five methods.

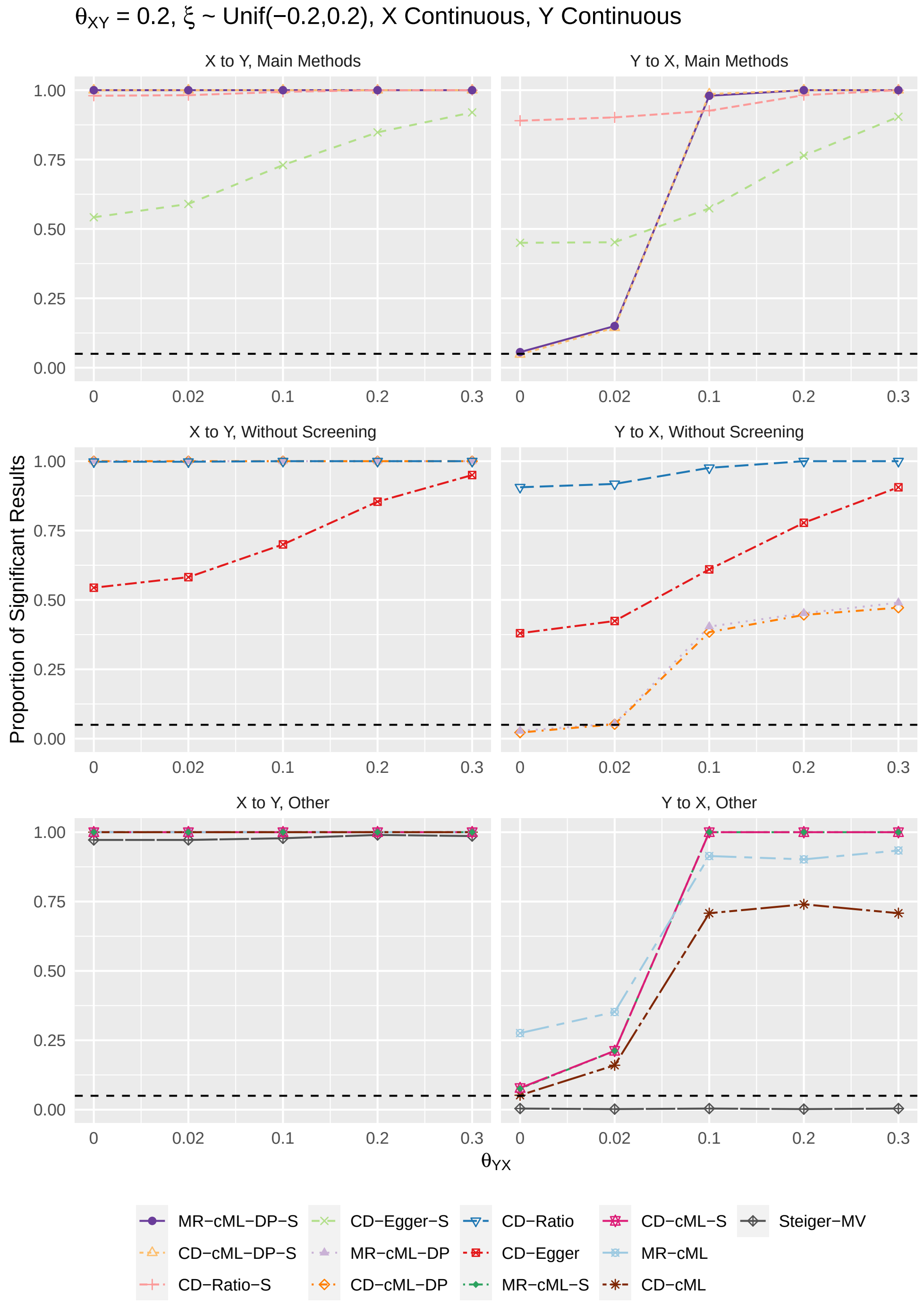

S9 Fig: When both $X$ and $Y$ are continuous, $\theta_{XY} = 0.3$ and $\xi = 0$, the proportions of significant simulation results obtained by the methods for direction $X \to Y$ (left column) and $Y \to X$ (right column). The first row shows results for four main methods: MR-cML-DP-S, CD-cML-DP-S, CD-Ratio-S, and CD-Egger-S; the second row shows results for four methods without screening: MR-cML-DP, CD-cML-DP, CD-Ratio, and CD-Egger; the third row shows results for other five methods.

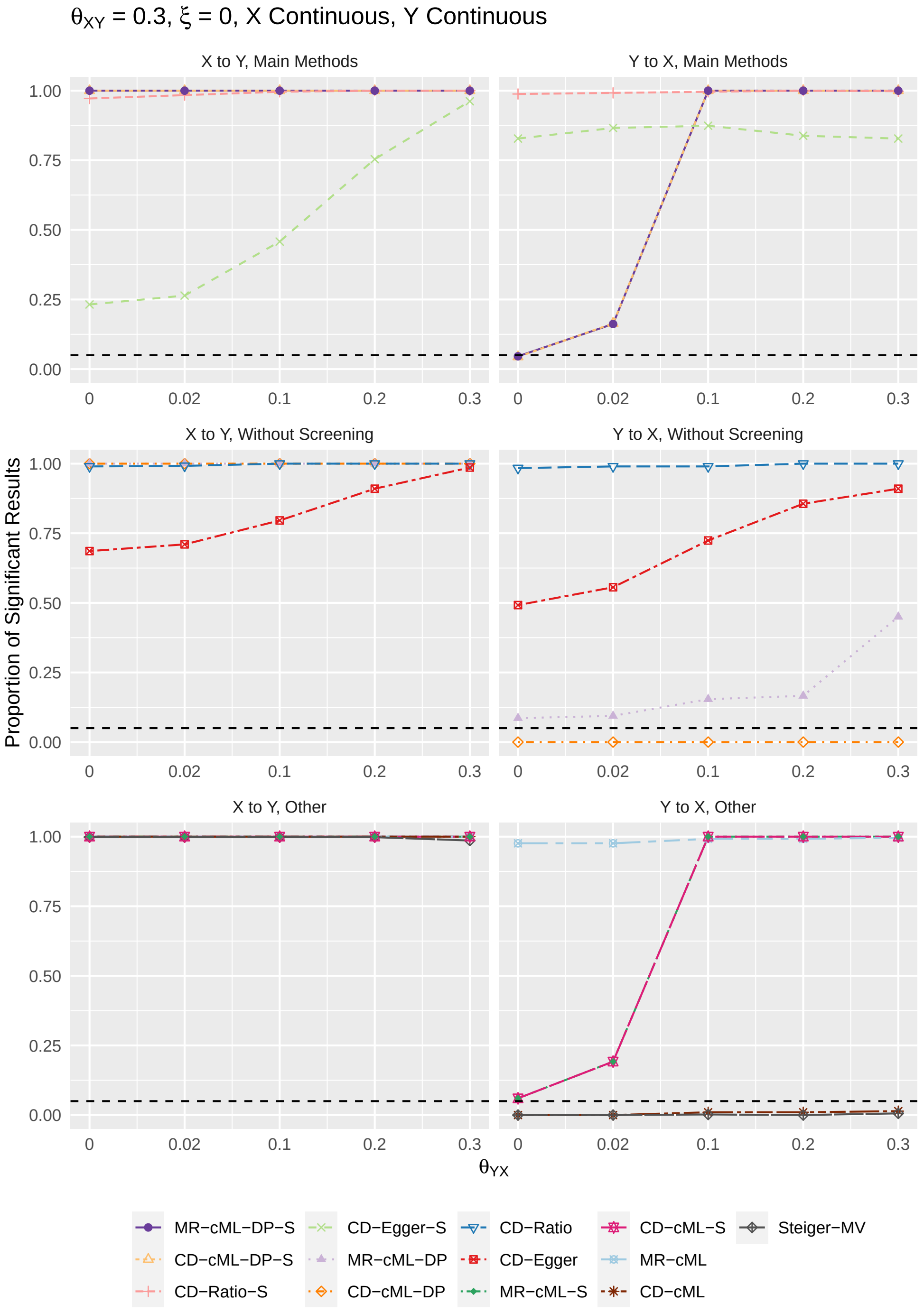

S10 Fig: When both $X$ and $Y$ are continuous, $\theta_{XY} = 0.3$ and $\xi \sim$ Unif(-0.2,0.2), the proportions of significant simulation results obtained by the methods for direction $X \to Y$ (left column) and $Y \to X$ (right column). The first row shows results for four main methods: MR-cML-DP-S, CD-cML-DP-S, CD-Ratio-S, and CD-Egger-S; the second row shows results for four methods without screening: MR-cML-DP, CD-cML-DP, CD-Ratio, and CD-Egger; the third row shows results for other five methods.

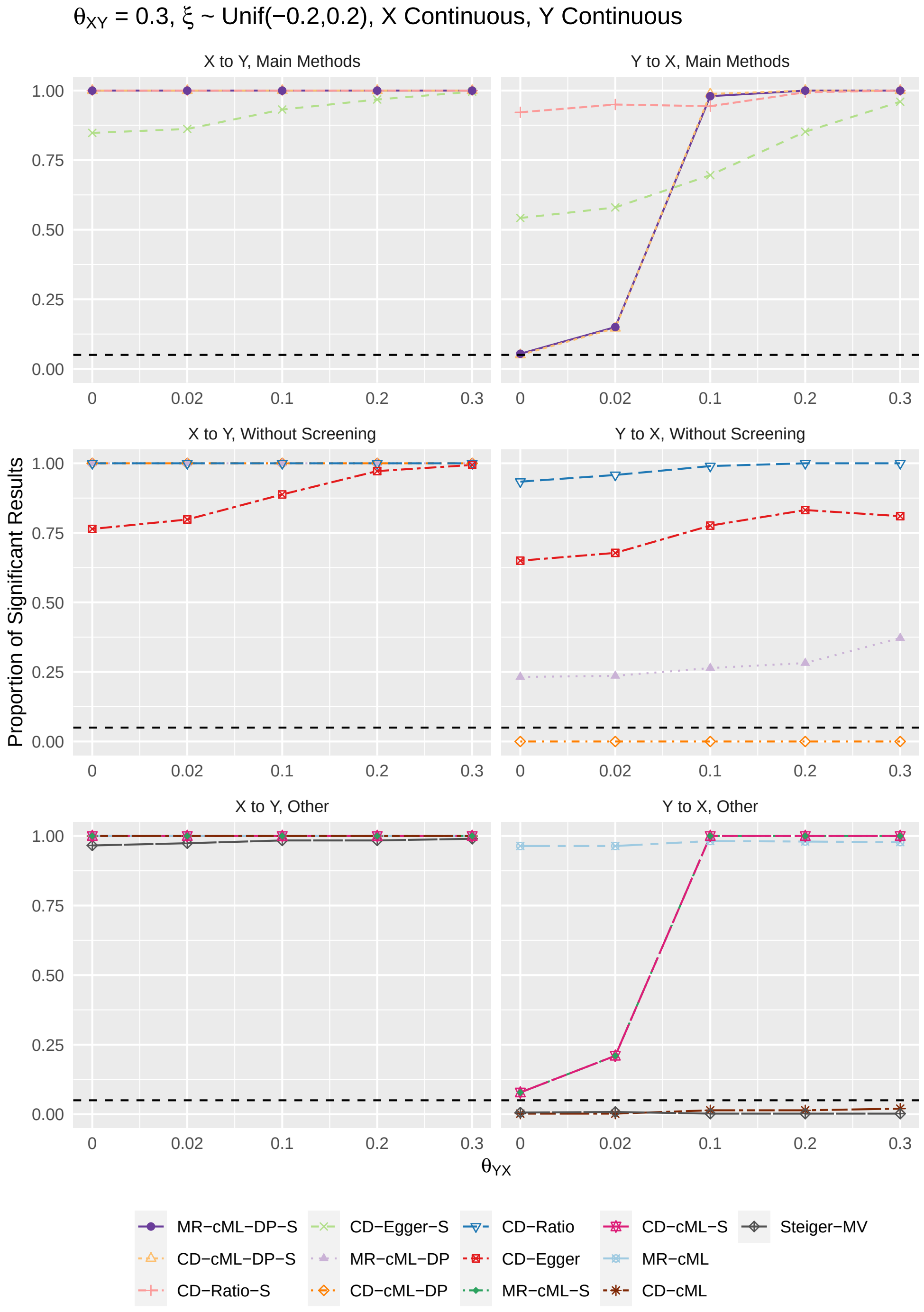

S11 Fig: When $X$ is binary, $Y$ is continuous, $\theta_{XY} = 0$ and $\xi = 0$, the proportions of significant simulation results obtained by the methods for direction $X \to Y$ (left column) and $Y \to X$ (right column). The first row shows results for four main methods: MR-cML-DP-S, CD-cML-DP-S, CD-Ratio-S, and CD-Egger-S; the second row shows results for four methods without screening: MR-cML-DP, CD-cML-DP, CD-Ratio, and CD-Egger; the third row shows results for other five methods.

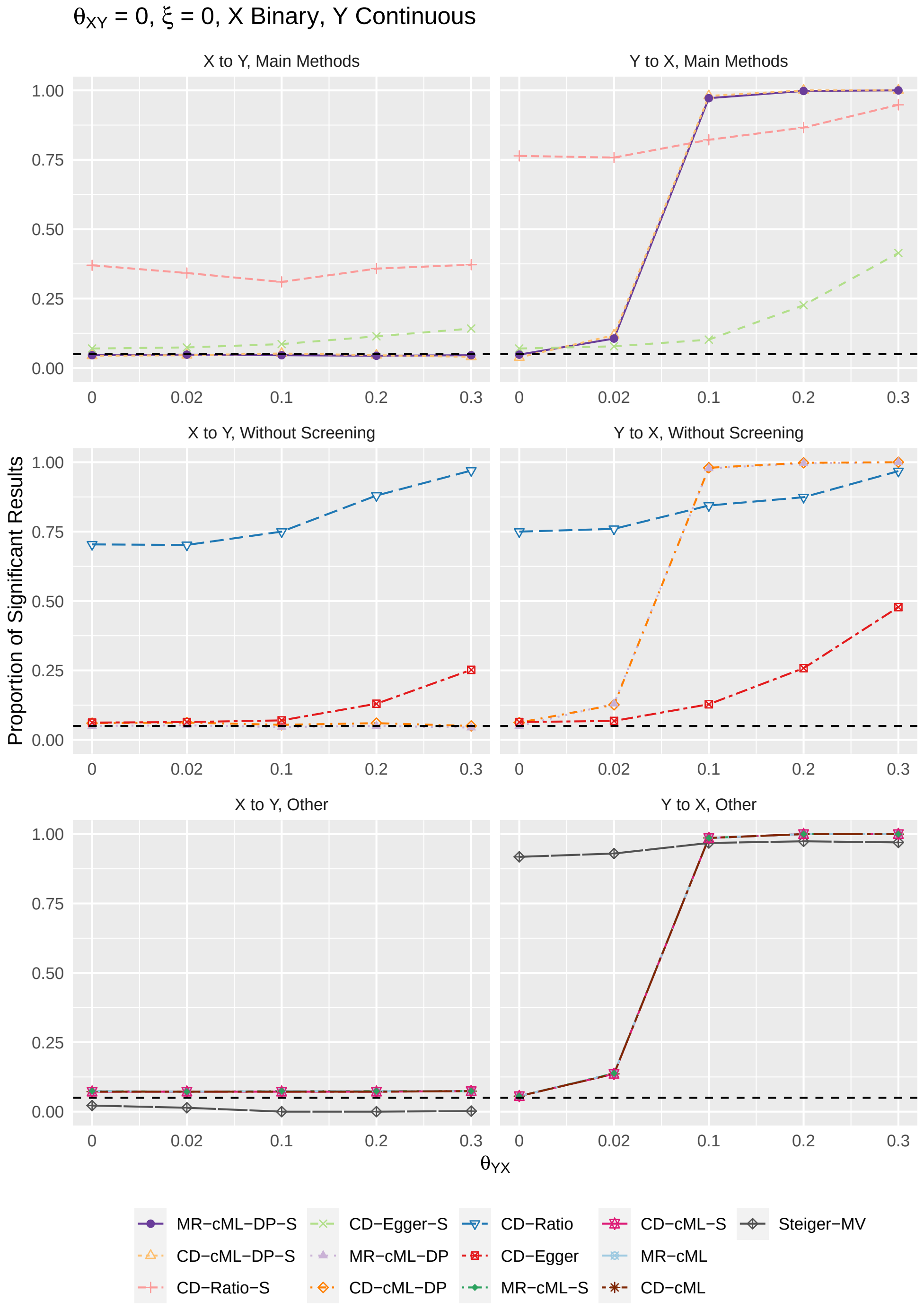

S12 Fig: When $X$ is binary, $Y$ is continuous, $\theta_{XY} = 0$ and $\xi \sim$ Unif(-0.2,0.2), the proportions of significant simulation results obtained by the methods for direction $X \to Y$ (left column) and $Y \to X$ (right column). The first row shows results for four main methods: MR-cML-DP-S, CD-cML-DP-S, CD-Ratio-S, and CD-Egger-S; the second row shows results for four methods without screening: MR-cML-DP, CD-cML-DP, CD-Ratio, and CD-Egger; the third row shows results for other five methods.

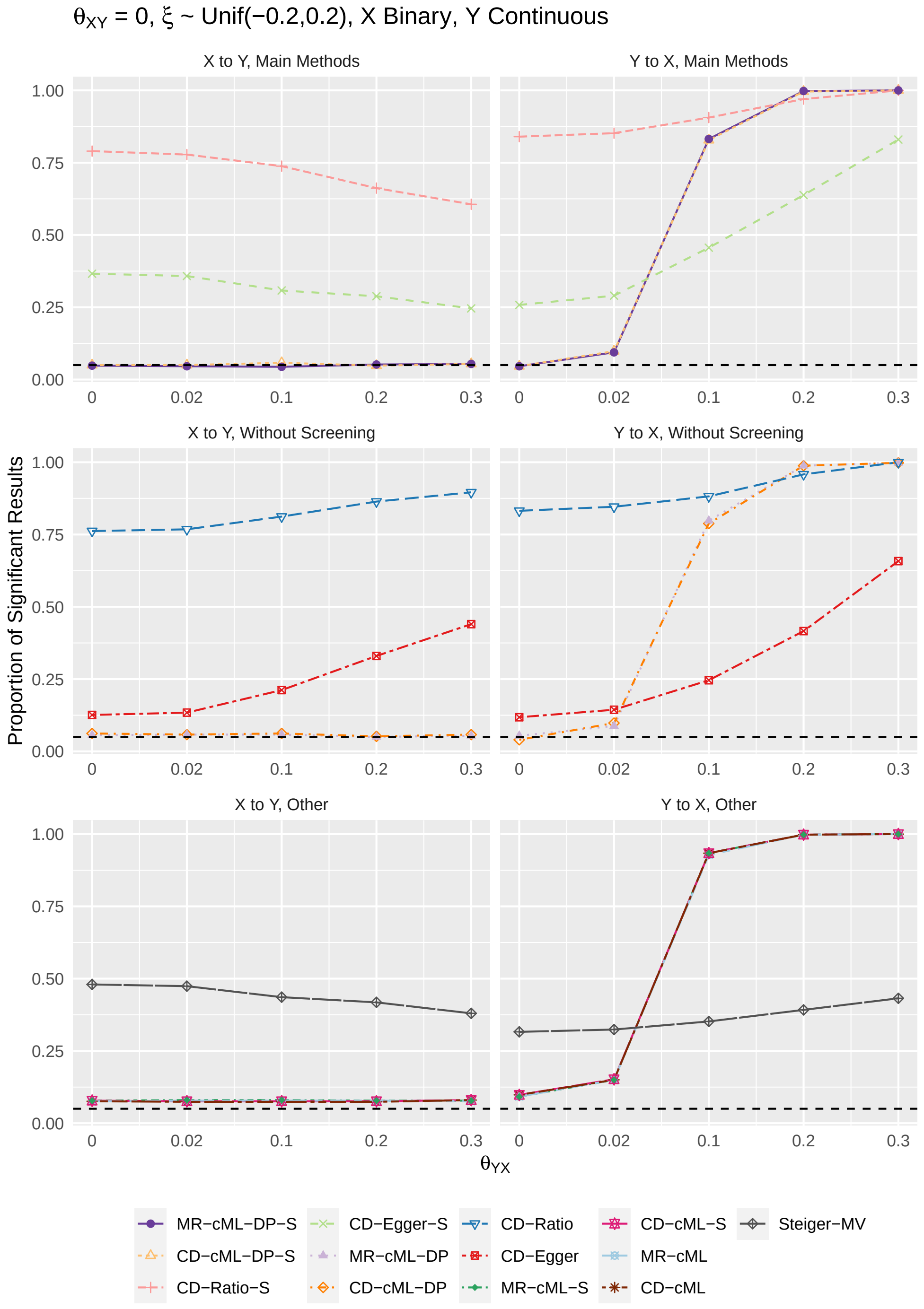

S13 Fig: When $X$ is binary, $Y$ is continuous, $\theta_{XY} = 0.02$ and $\xi = 0$, the proportions of significant simulation results obtained by the methods for direction $X \to Y$ (left column) and $Y \to X$ (right column). The first row shows results for four main methods: MR-cML-DP-S, CD-cML-DP-S, CD-Ratio-S, and CD-Egger-S; the second row shows results for four methods without screening: MR-cML-DP, CD-cML-DP, CD-Ratio, and CD-Egger; the third row shows results for other five methods.

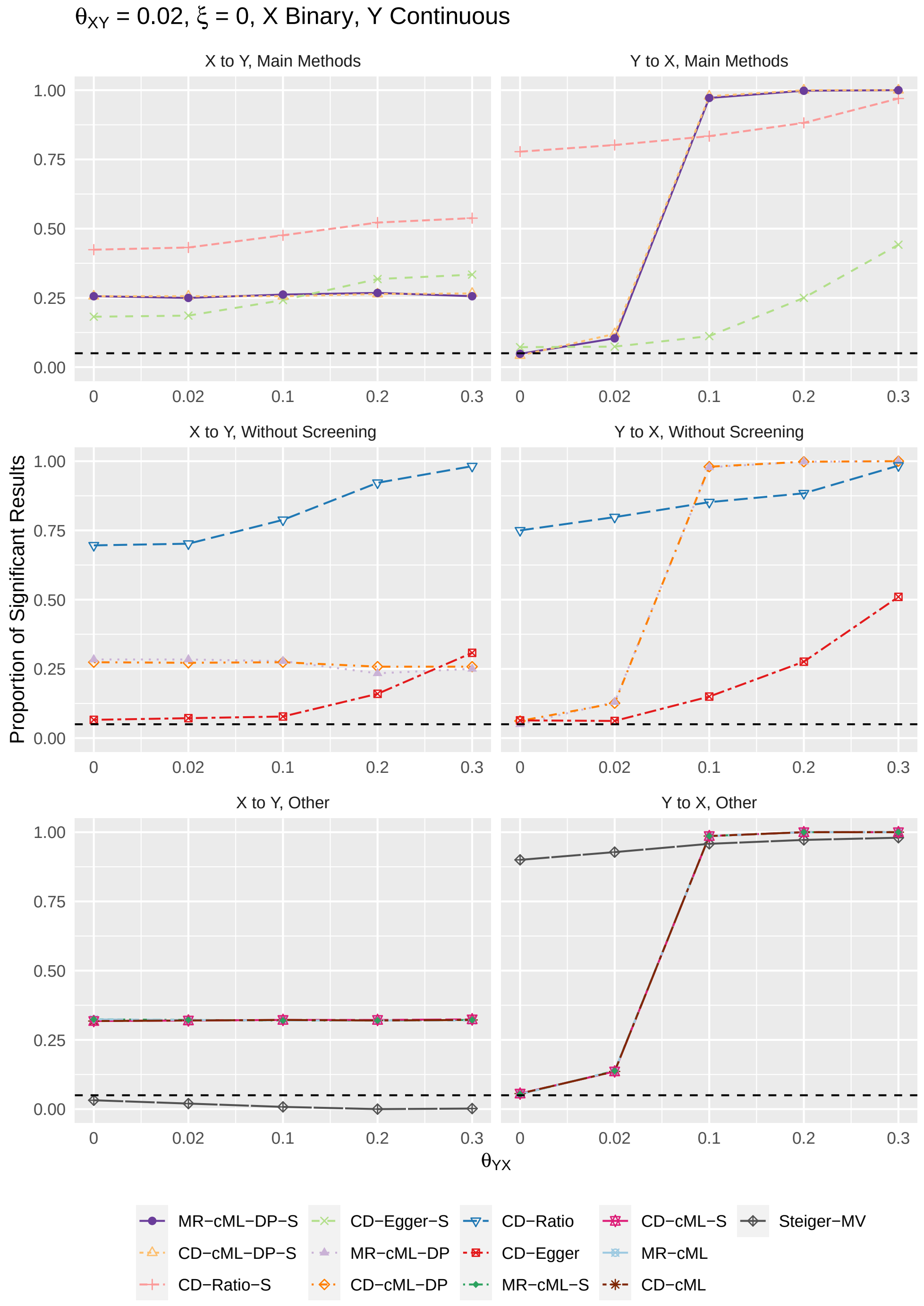

S14 Fig: When $X$ is binary, $Y$ is continuous, $\theta_{XY} = 0.02$ and $\xi \sim$ Unif(-0.2,0.2), the proportions of significant simulation results obtained by the methods for direction $X \to Y$ (left column) and $Y \to X$ (right column). The first row shows results for four main methods: MR-cML-DP-S, CD-cML-DP-S, CD-Ratio-S, and CD-Egger-S; the second row shows results for four methods without screening: MR-cML-DP, CD-cML-DP, CD-Ratio, and CD-Egger; the third row shows results for other five methods.

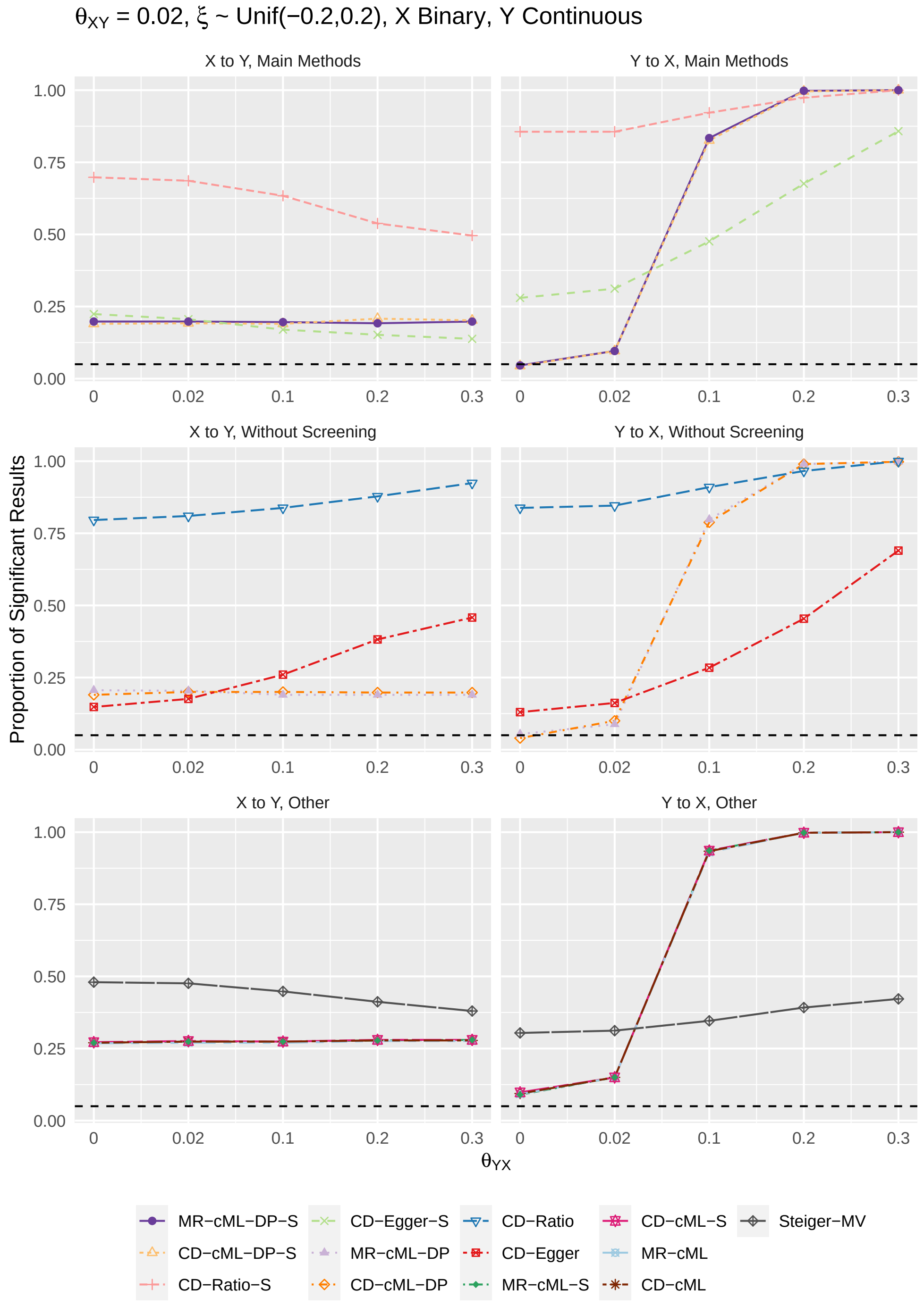

S15 Fig: When $X$ is binary, $Y$ is continuous, $\theta_{XY} = 0.1$ and $\xi = 0$, the proportions of significant simulation results obtained by the methods for direction $X \to Y$ (left column) and $Y \to X$ (right column). The first row shows results for four main methods: MR-cML-DP-S, CD-cML-DP-S, CD-Ratio-S, and CD-Egger-S; the second row shows results for four methods without screening: MR-cML-DP, CD-cML-DP, CD-Ratio, and CD-Egger; the third row shows results for other five methods.

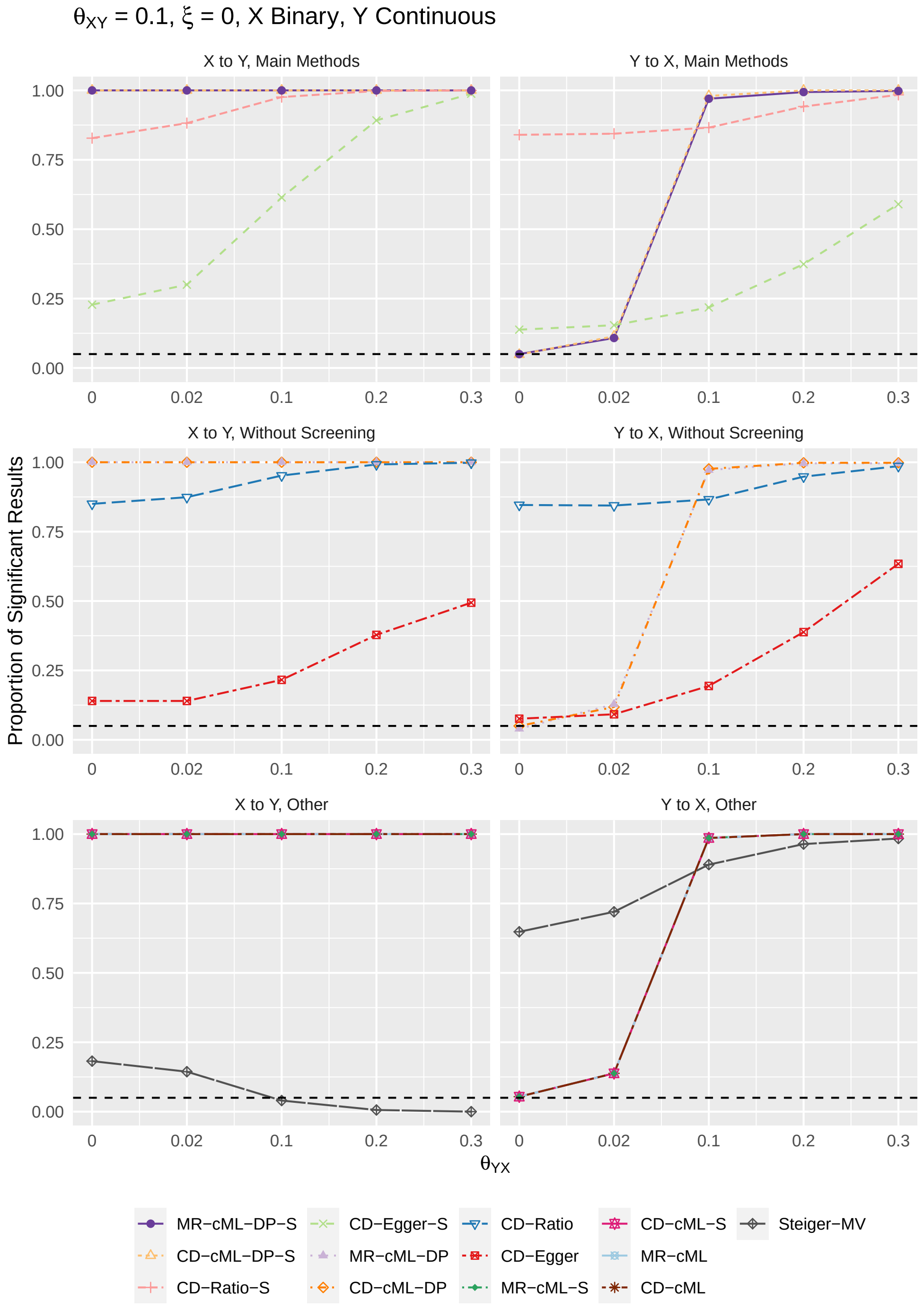

S16 Fig: When $X$ is binary, $Y$ is continuous, $\theta_{XY} = 0.1$ and $\xi \sim$ Unif(-0.2,0.2), the proportions of significant simulation results obtained by the methods for direction $X \to Y$ (left column) and $Y \to X$ (right column). The first row shows results for four main methods: MR-cML-DP-S, CD-cML-DP-S, CD-Ratio-S, and CD-Egger-S; the second row shows results for four methods without screening: MR-cML-DP, CD-cML-DP, CD-Ratio, and CD-Egger; the third row shows results for other five methods.

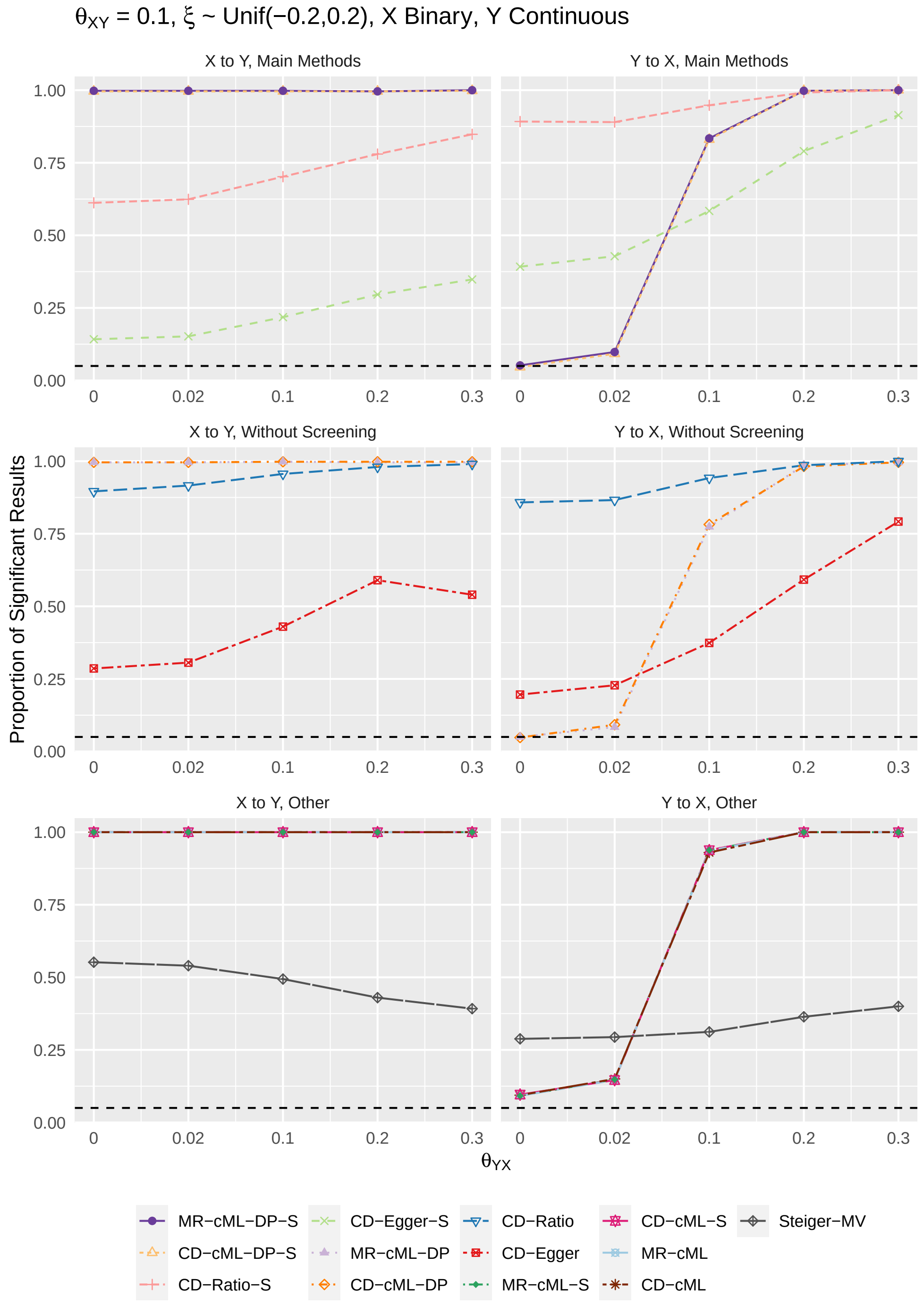

S17 Fig: When $X$ is binary, $Y$ is continuous, $\theta_{XY} = 0.2$ and $\xi = 0$, the proportions of significant simulation results obtained by the methods for direction $X \to Y$ (left column) and $Y \to X$ (right column). The first row shows results for four main methods: MR-cML-DP-S, CD-cML-DP-S, CD-Ratio-S, and CD-Egger-S; the second row shows results for four methods without screening: MR-cML-DP, CD-cML-DP, CD-Ratio, and CD-Egger; the third row shows results for other five methods.

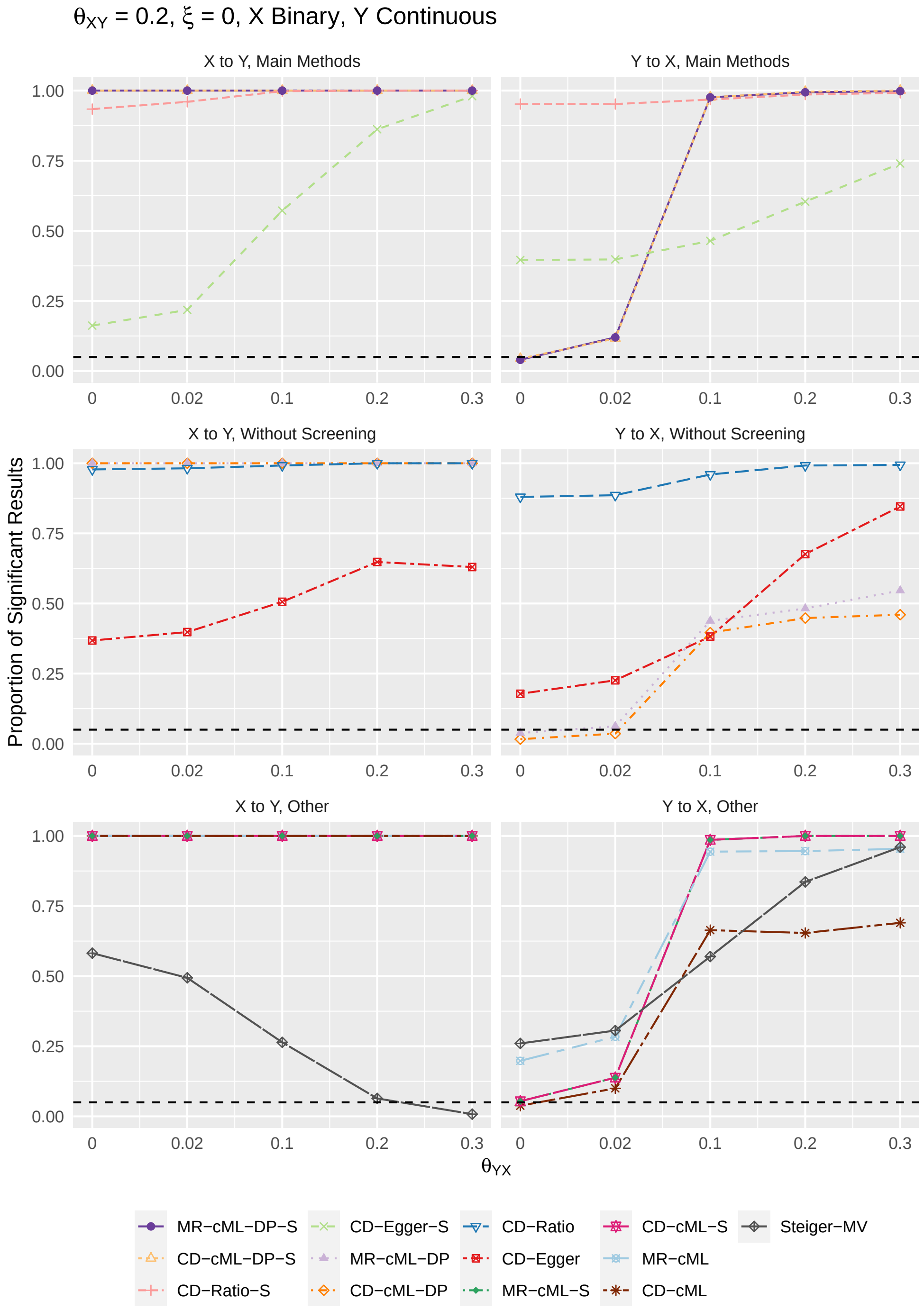

S18 Fig: When $X$ is binary, $Y$ is continuous, $\theta_{XY} = 0.2$ and $\xi \sim$ Unif(-0.2,0.2), the proportions of significant simulation results obtained by the methods for direction $X \to Y$ (left column) and $Y \to X$ (right column). The first row shows results for four main methods: MR-cML-DP-S, CD-cML-DP-S, CD-Ratio-S, and CD-Egger-S; the second row shows results for four methods without screening: MR-cML-DP, CD-cML-DP, CD-Ratio, and CD-Egger; the third row shows results for other five methods.

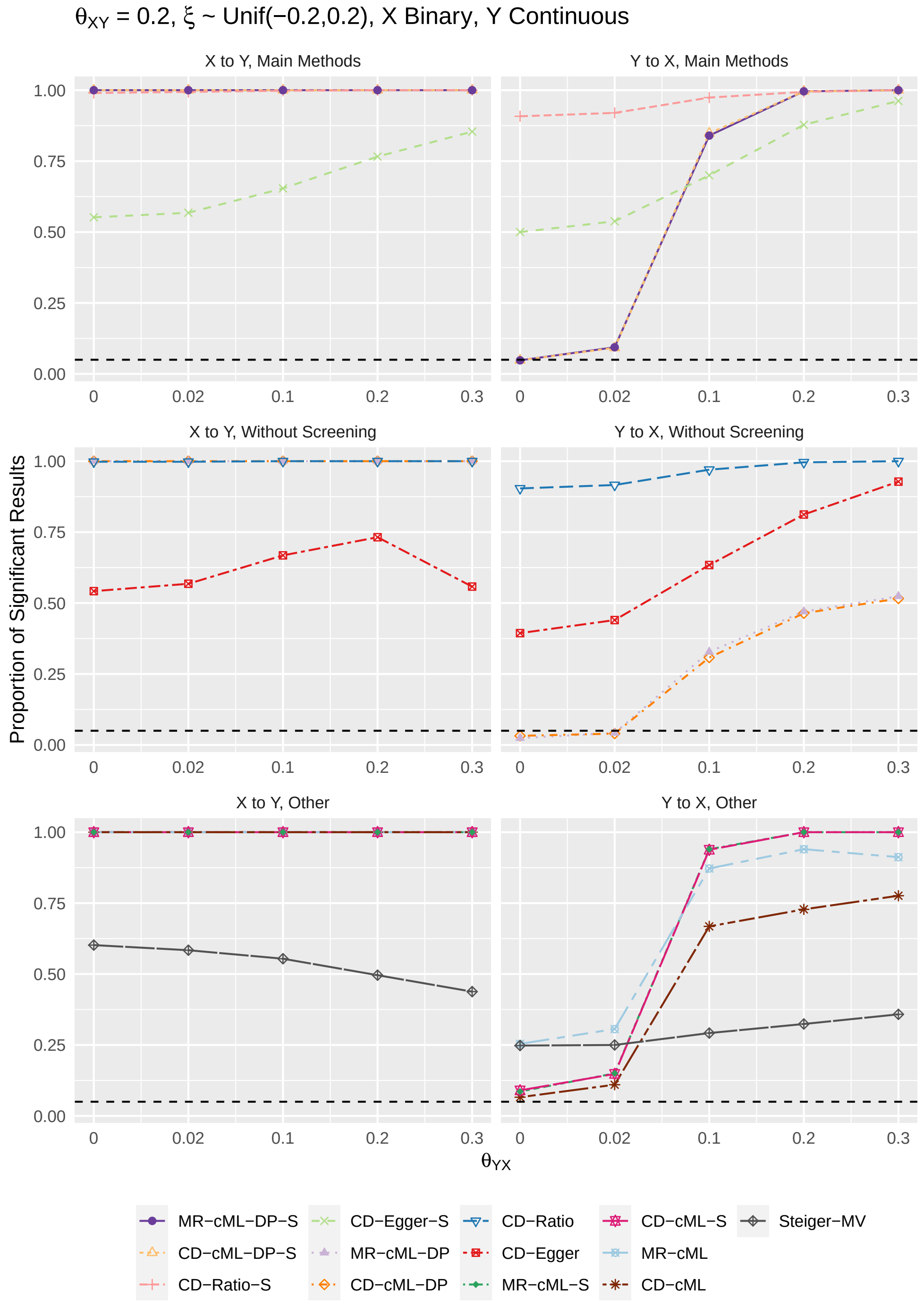

S19 Fig: When $X$ is binary, $Y$ is continuous, $\theta_{XY} = 0.3$ and $\xi = 0$, the proportions of significant simulation results obtained by the methods for direction $X \to Y$ (left column) and $Y \to X$ (right column). The first row shows results for four main methods: MR-cML-DP-S, CD-cML-DP-S, CD-Ratio-S, and CD-Egger-S; the second row shows results for four methods without screening: MR-cML-DP, CD-cML-DP, CD-Ratio, and CD-Egger; the third row shows results for other five methods.

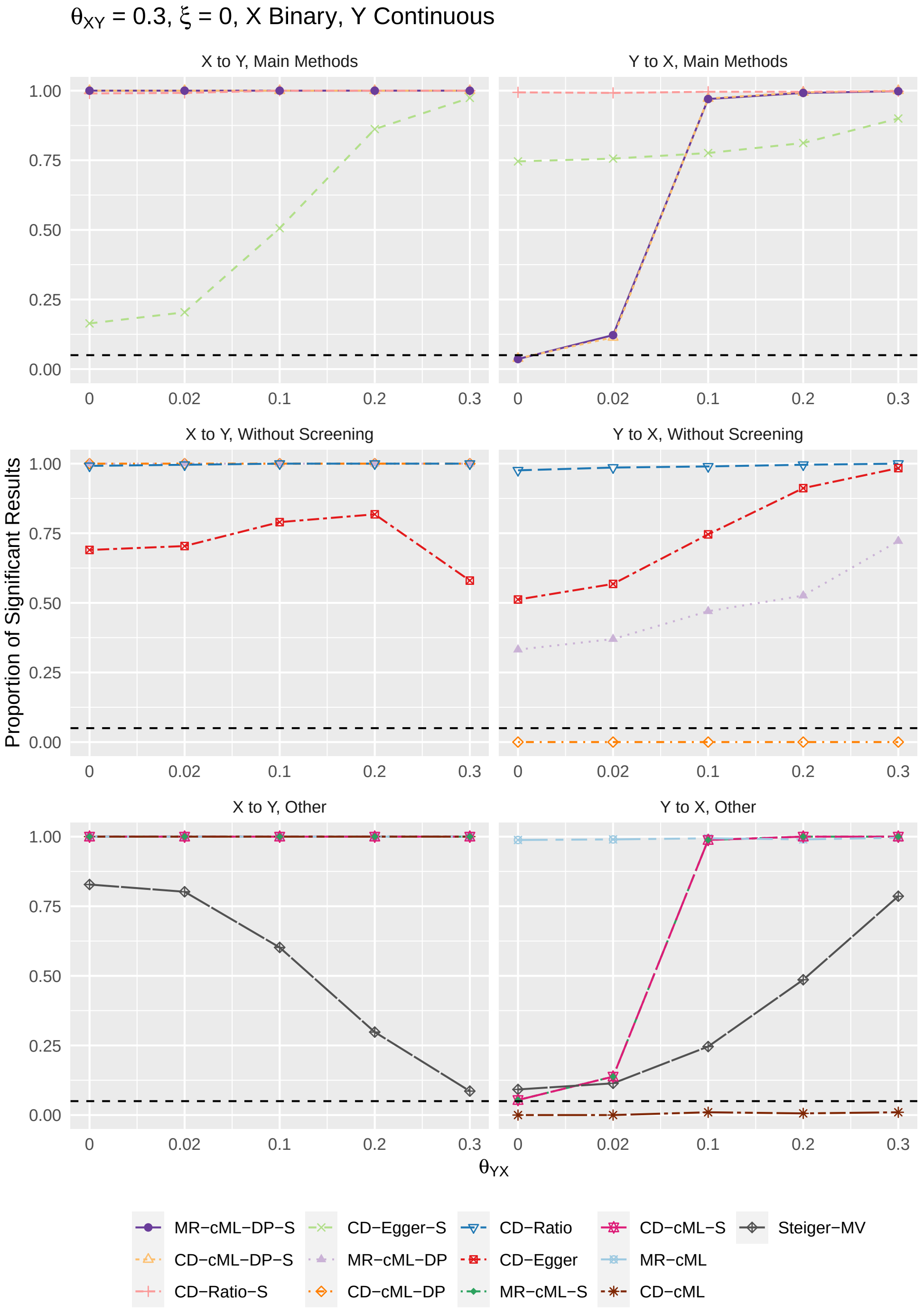

S20 Fig: When $X$ is binary, $Y$ is continuous, $\theta_{XY} = 0.3$ and $\xi \sim$ Unif(-0.2,0.2), the proportions of significant simulation results obtained by the methods for direction $X \to Y$ (left column) and $Y \to X$ (right column). The first row shows results for four main methods: MR-cML-DP-S, CD-cML-DP-S, CD-Ratio-S, and CD-Egger-S; the second row shows results for four methods without screening: MR-cML-DP, CD-cML-DP, CD-Ratio, and CD-Egger; the third row shows results for other five methods.

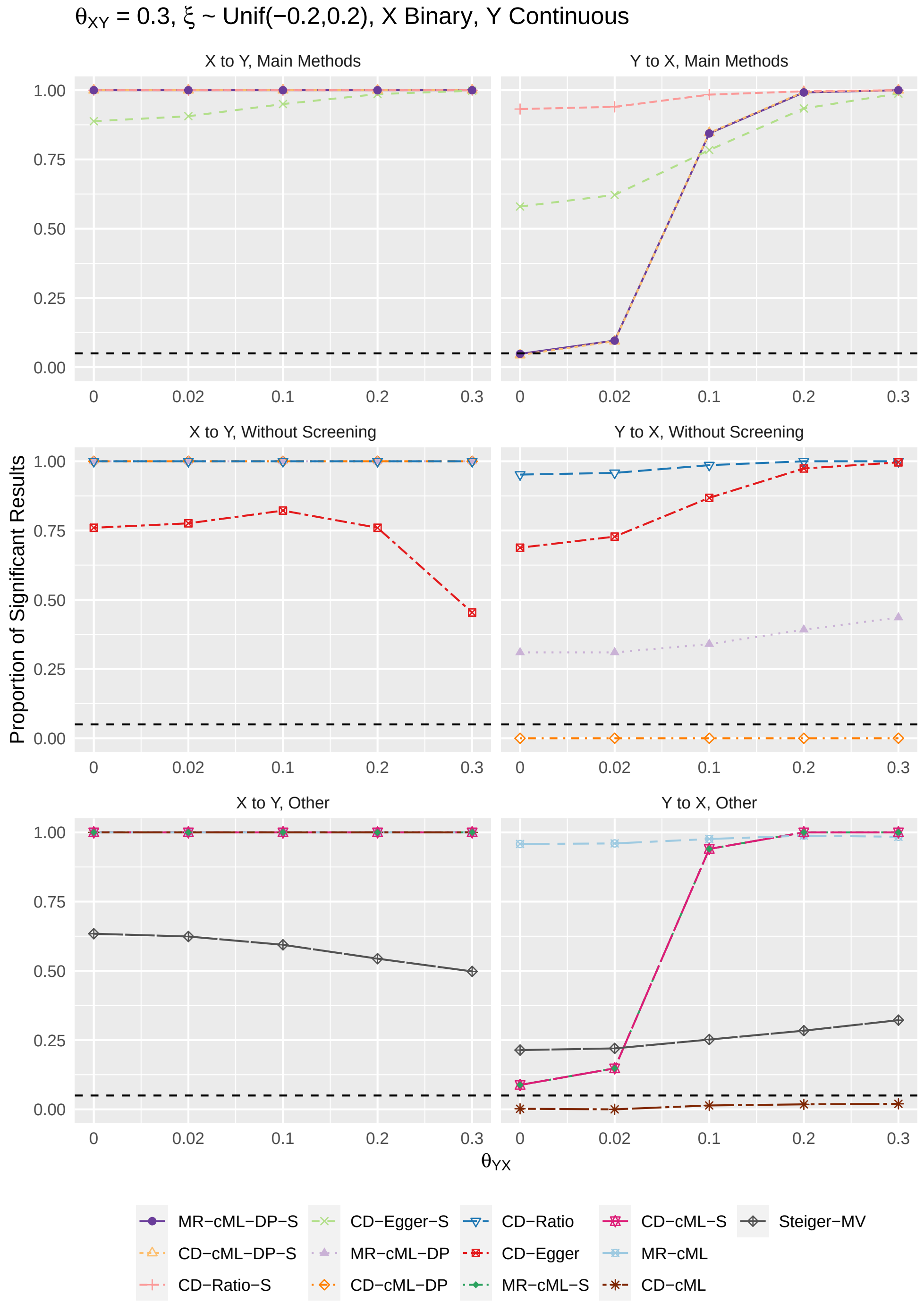

S21 Fig: When $X$ is continuous, $Y$ is binary, $\theta_{XY} = 0$ and $\xi = 0$, the proportions of significant simulation results obtained by the methods for direction $X \to Y$ (left column) and $Y \to X$ (right column). The first row shows results for four main methods: MR-cML-DP-S, CD-cML-DP-S, CD-Ratio-S, and CD-Egger-S; the second row shows results for four methods without screening: MR-cML-DP, CD-cML-DP, CD-Ratio, and CD-Egger; the third row shows results for other five methods.

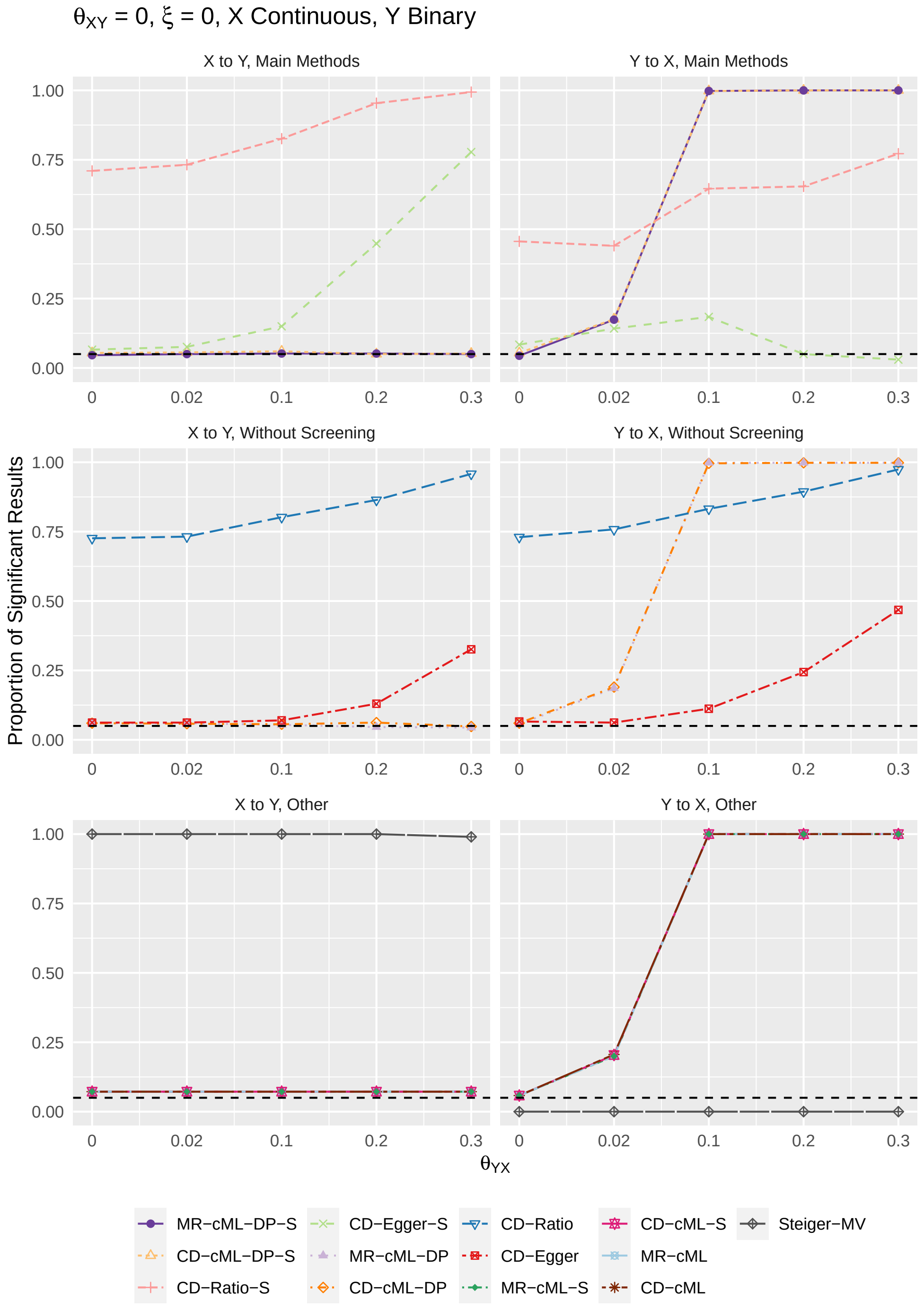

S22 Fig: When $X$ is continuous, $Y$ is binary, $\theta_{XY} = 0$ and $\xi \sim$ Unif(-0.2,0.2), the proportions of significant simulation results obtained by the methods for direction $X \to Y$ (left column) and $Y \to X$ (right column). The first row shows results for four main methods: MR-cML-DP-S, CD-cML-DP-S, CD-Ratio-S, and CD-Egger-S; the second row shows results for four methods without screening: MR-cML-DP, CD-cML-DP, CD-Ratio, and CD-Egger; the third row shows results for other five methods.

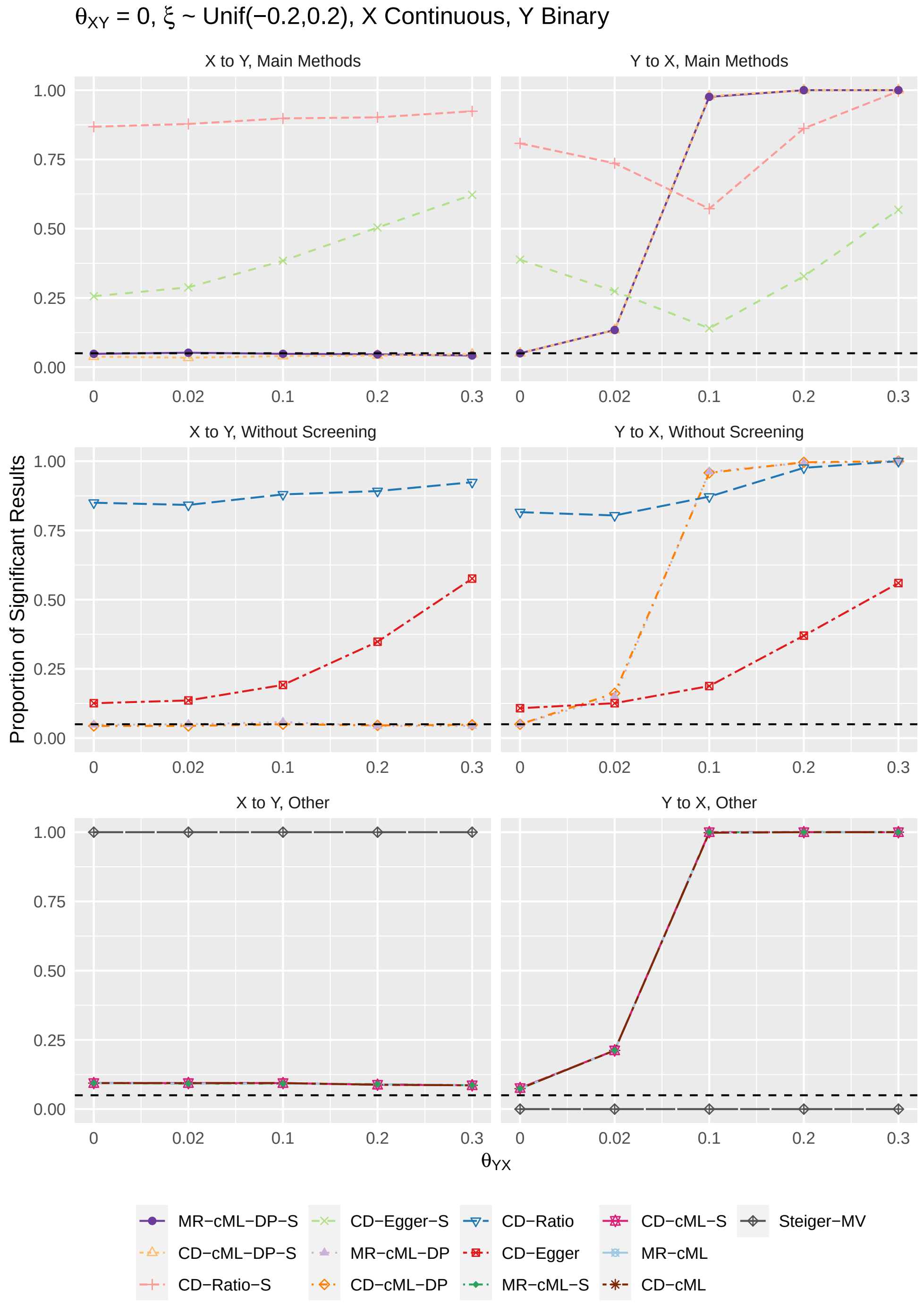

S23 Fig: When $X$ is continuous, $Y$ is binary, $\theta_{XY} = 0.02$ and $\xi = 0$, the proportions of significant simulation results obtained by the methods for direction $X \to Y$ (left column) and $Y \to X$ (right column). The first row shows results for four main methods: MR-cML-DP-S, CD-cML-DP-S, CD-Ratio-S, and CD-Egger-S; the second row shows results for four methods without screening: MR-cML-DP, CD-cML-DP, CD-Ratio, and CD-Egger; the third row shows results for other five methods.

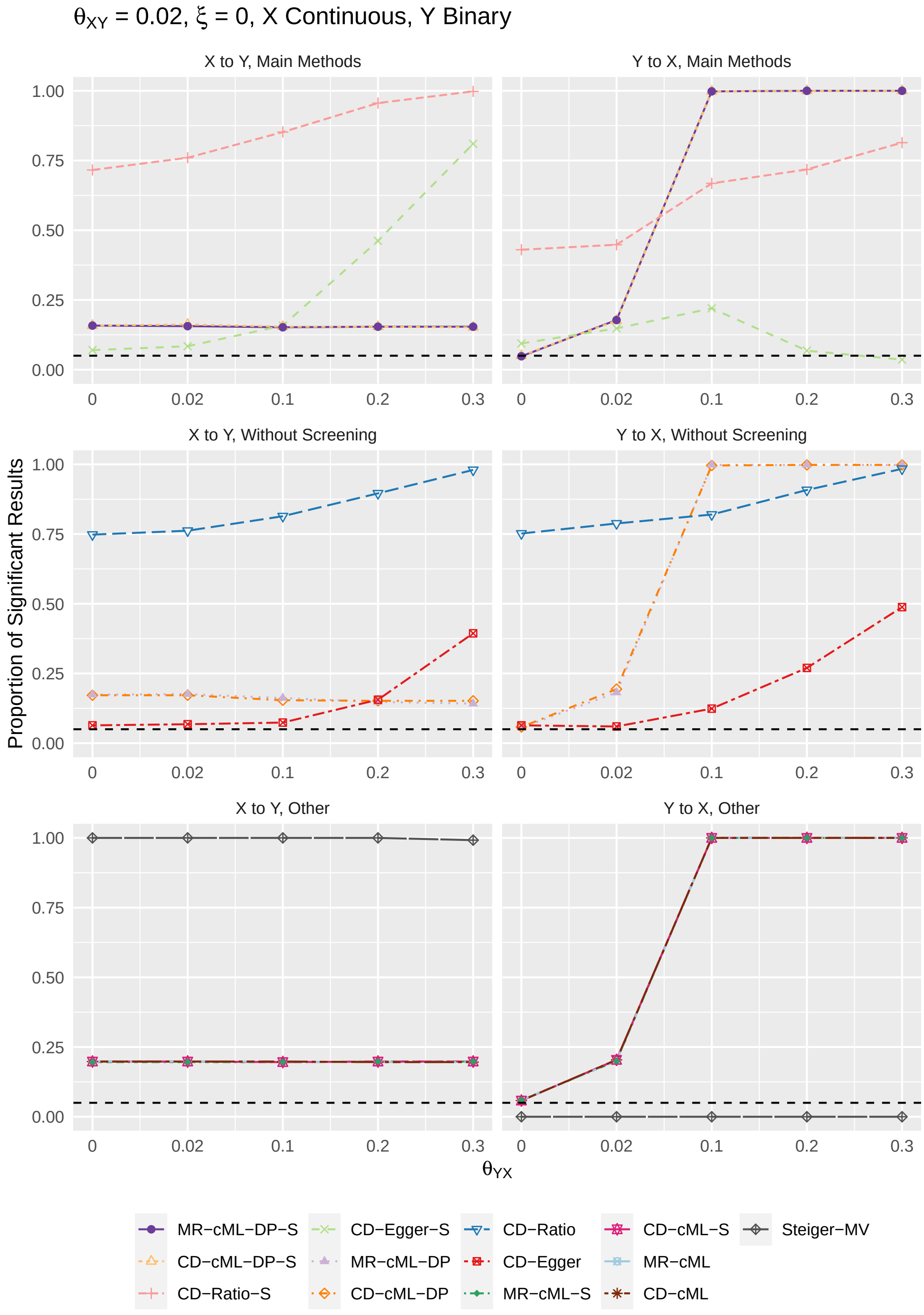

S24 Fig: When $X$ is continuous, $Y$ is binary, $\theta_{XY} = 0.02$ and $\xi \sim$ Unif(-0.2,0.2), the proportions of significant simulation results obtained by the methods for direction $X \to Y$ (left column) and $Y \to X$ (right column). The first row shows results for four main methods: MR-cML-DP-S, CD-cML-DP-S, CD-Ratio-S, and CD-Egger-S; the second row shows results for four methods without screening: MR-cML-DP, CD-cML-DP, CD-Ratio, and CD-Egger; the third row shows results for other five methods.

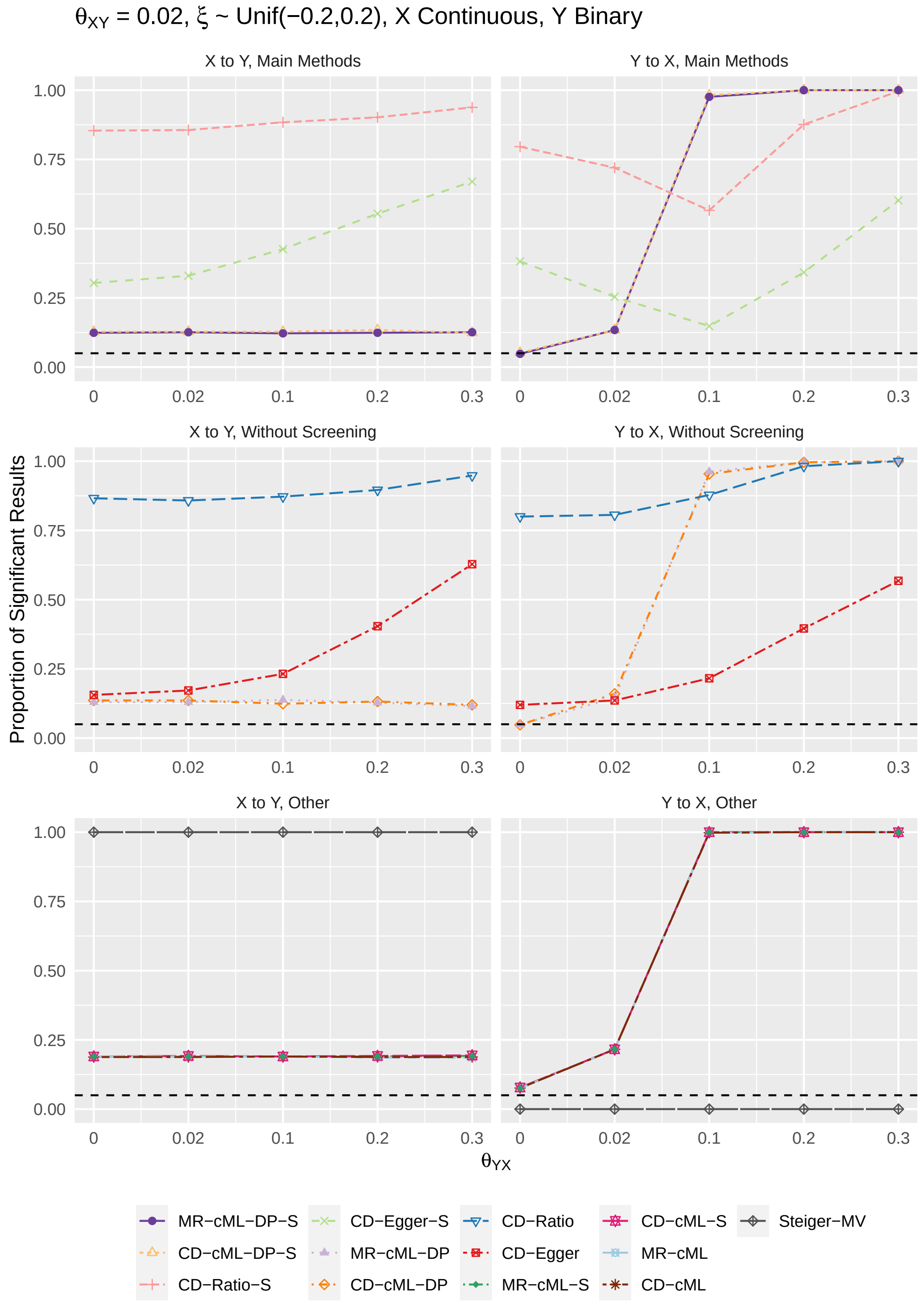

S25 Fig: When $X$ is continuous, $Y$ is binary, $\theta_{XY} = 0.1$ and $\xi = 0$, the proportions of significant simulation results obtained by the methods for direction $X \to Y$ (left column) and $Y \to X$ (right column). The first row shows results for four main methods: MR-cML-DP-S, CD-cML-DP-S, CD-Ratio-S, and CD-Egger-S; the second row shows results for four methods without screening: MR-cML-DP, CD-cML-DP, CD-Ratio, and CD-Egger; the third row shows results for other five methods.

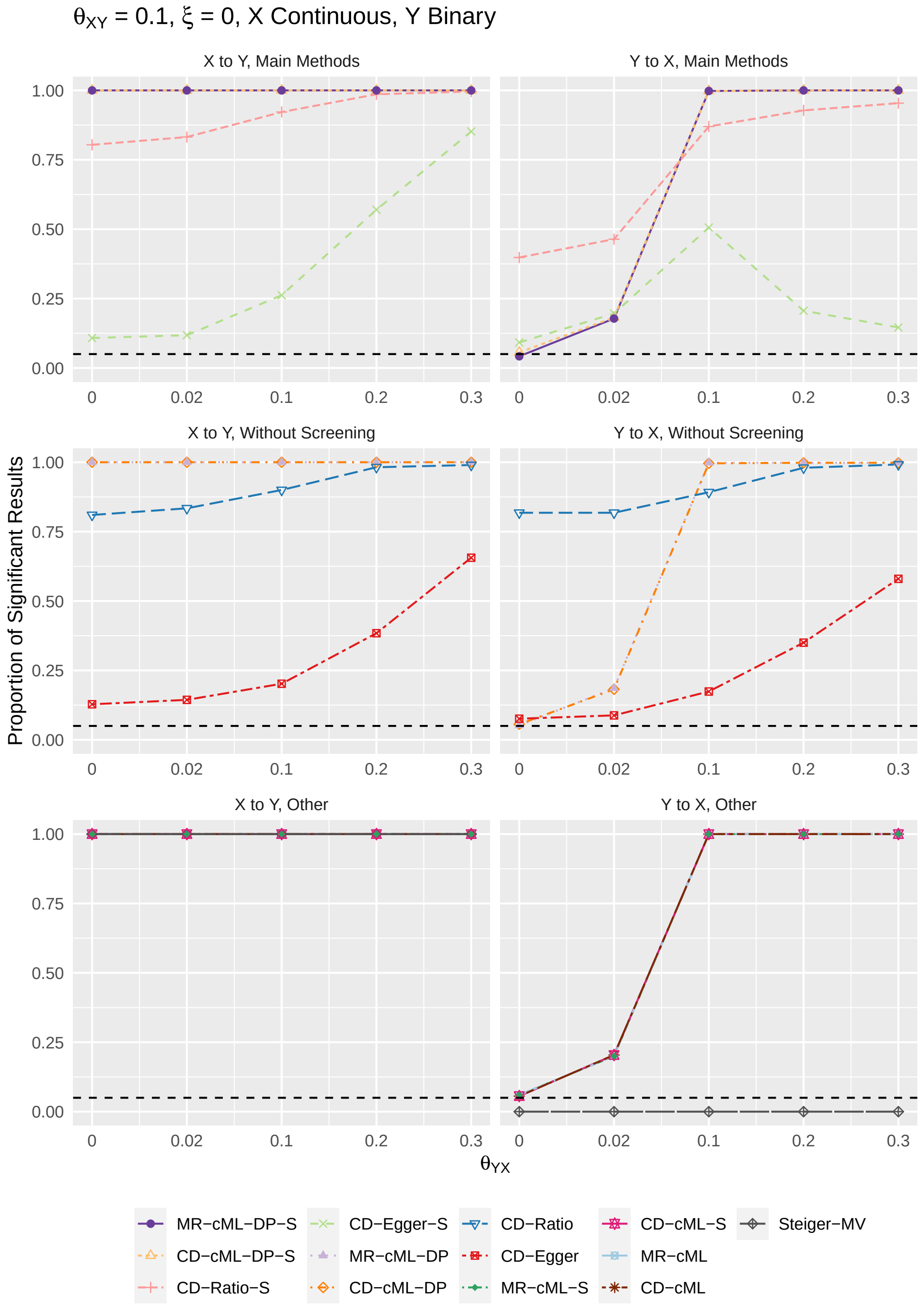

S26 Fig: When $X$ is continuous, $Y$ is binary, $\theta_{XY} = 0.1$ and $\xi \sim$ Unif(-0.2,0.2), the proportions of significant simulation results obtained by the methods for direction $X \rightarrow Y$ (left column) and $Y \rightarrow X$ (right column). The first row shows results for four main methods: MR-cML-DP-S, CD-cML-DP-S, CD-Ratio-S, and CD-Egger-S; the second row shows results for four methods without screening: MR-cML-DP, CD-cML-DP, CD-Ratio, and CD-Egger; the third row shows results for other five methods.

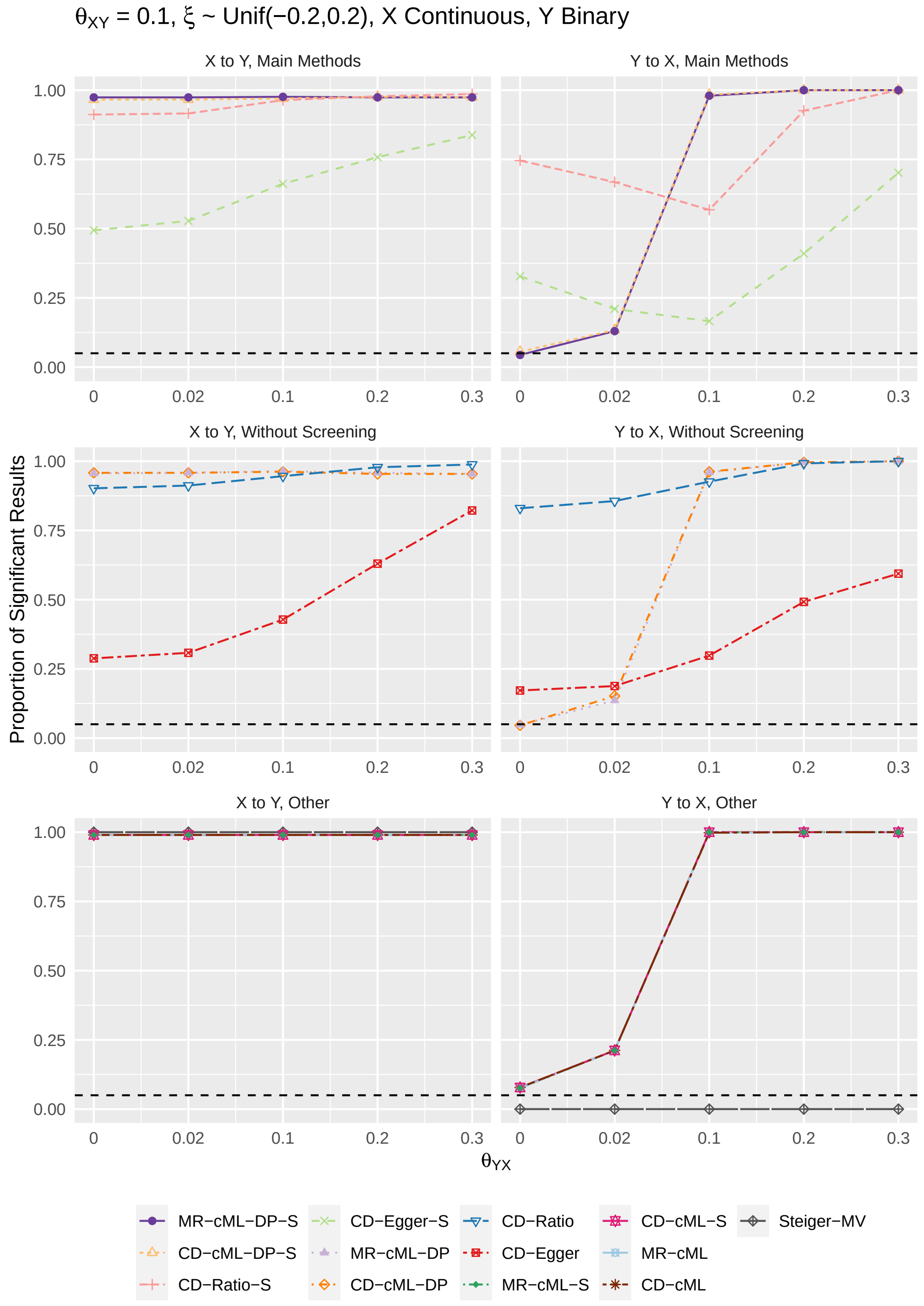

S27 Fig: When $X$ is continuous, $Y$ is binary, $\theta_{XY} = 0.2$ and $\xi = 0$, the proportions of significant simulation results obtained by the methods for direction $X \to Y$ (left column) and $Y \to X$ (right column). The first row shows results for four main methods: MR-cML-DP-S, CD-cML-DP-S, CD-Ratio-S, and CD-Egger-S; the second row shows results for four methods without screening: MR-cML-DP, CD-cML-DP, CD-Ratio, and CD-Egger; the third row shows results for other five methods.

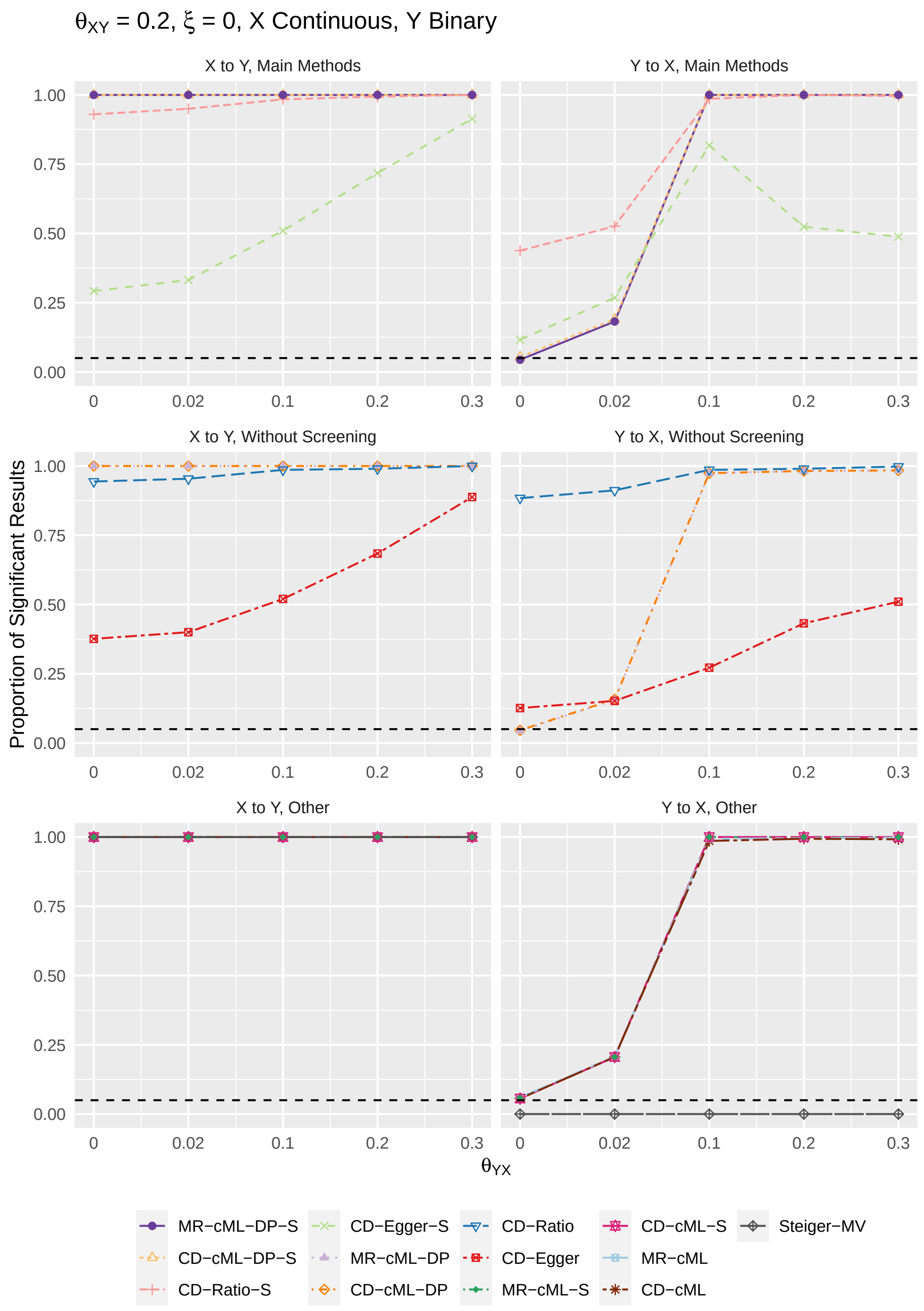

S28 Fig: When $X$ is continuous, $Y$ is binary, $\theta_{XY} = 0.2$ and $\xi \sim$ Unif(-0.2,0.2), the proportions of significant simulation results obtained by the methods for direction $X \to Y$ (left column) and $Y \to X$ (right column). The first row shows results for four main methods: MR-cML-DP-S, CD-cML-DP-S, CD-Ratio-S, and CD-Egger-S; the second row shows results for four methods without screening: MR-cML-DP, CD-cML-DP, CD-Ratio, and CD-Egger; the third row shows results for other five methods.

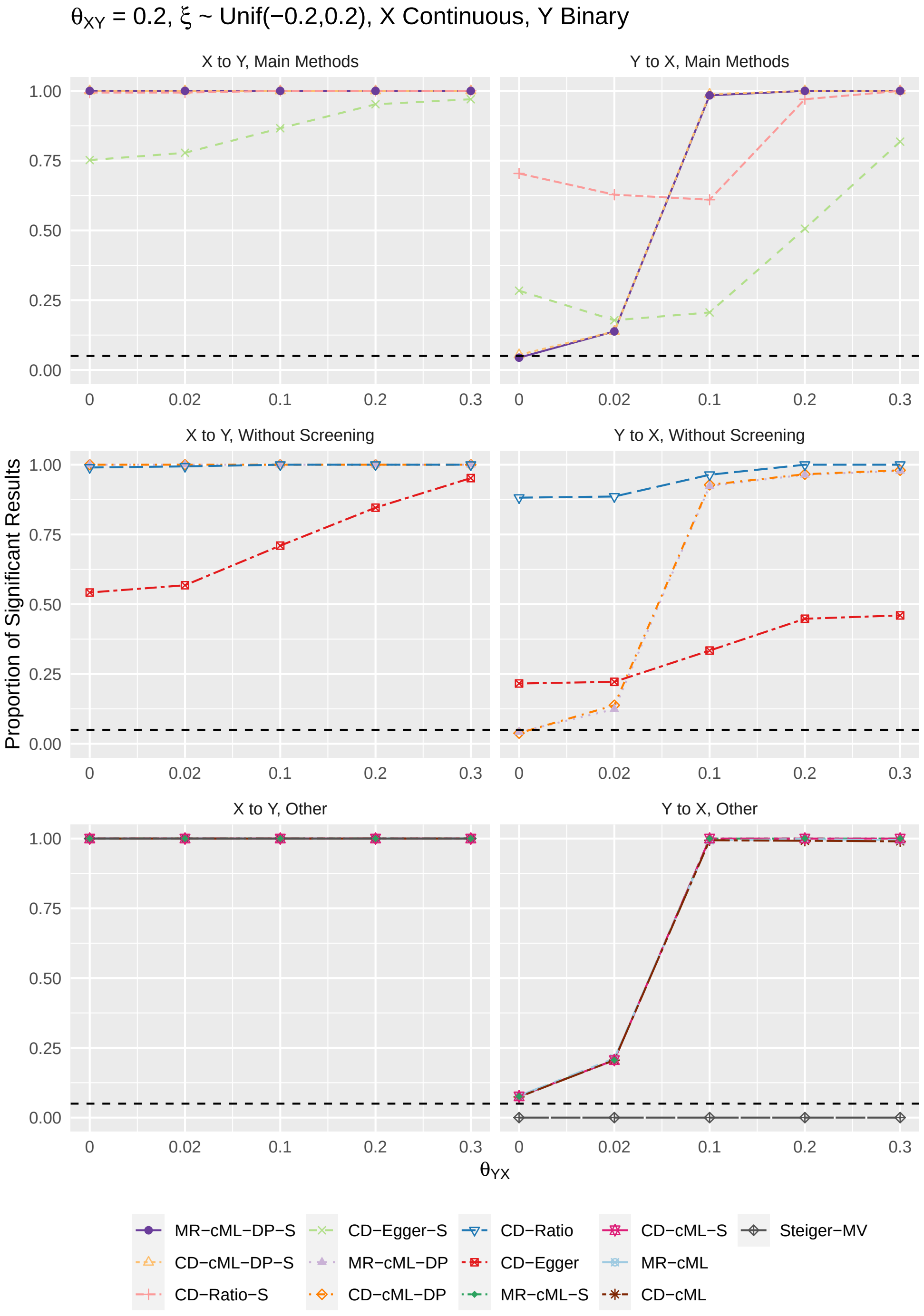

S29 Fig: When $X$ is continuous, $Y$ is binary, $\theta_{XY} = 0.3$ and $\xi = 0$, the proportions of significant simulation results obtained by the methods for direction $X \to Y$ (left column) and $Y \to X$ (right column). The first row shows results for four main methods: MR-cML-DP-S, CD-cML-DP-S, CD-Ratio-S, and CD-Egger-S; the second row shows results for four methods without screening: MR-cML-DP, CD-cML-DP, CD-Ratio, and CD-Egger; the third row shows results for other five methods.

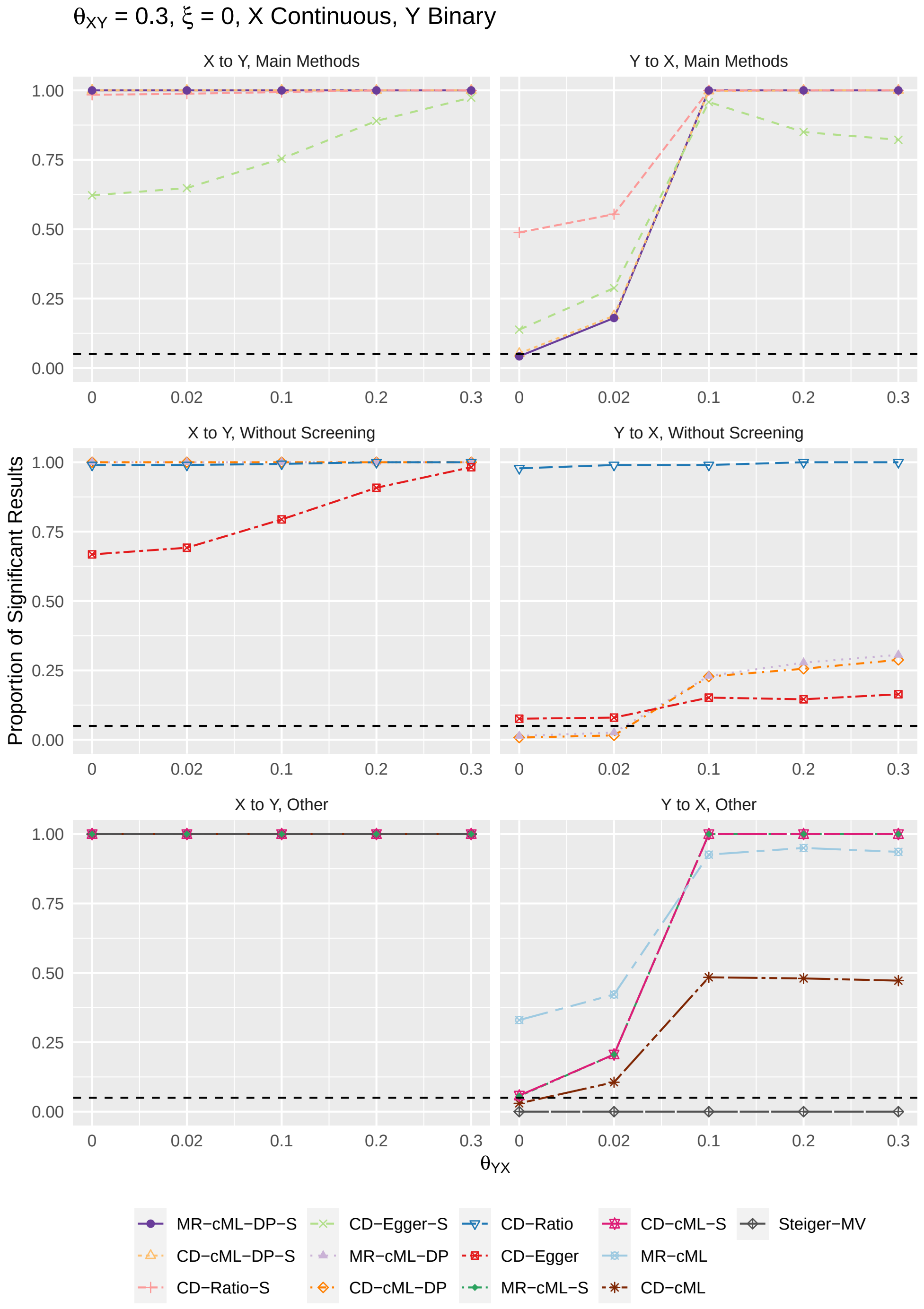

S30 Fig: When $X$ is continuous, $Y$ is binary, $\theta_{XY} = 0.3$ and $\xi \sim$ Unif(-0.2,0.2), the proportions of significant simulation results obtained by the methods for direction $X \to Y$ (left column) and $Y \to X$ (right column). The first row shows results for four main methods: MR-cML-DP-S, CD-cML-DP-S, CD-Ratio-S, and CD-Egger-S; the second row shows results for four methods without screening: MR-cML-DP, CD-cML-DP, CD-Ratio, and CD-Egger; the third row shows results for other five methods.

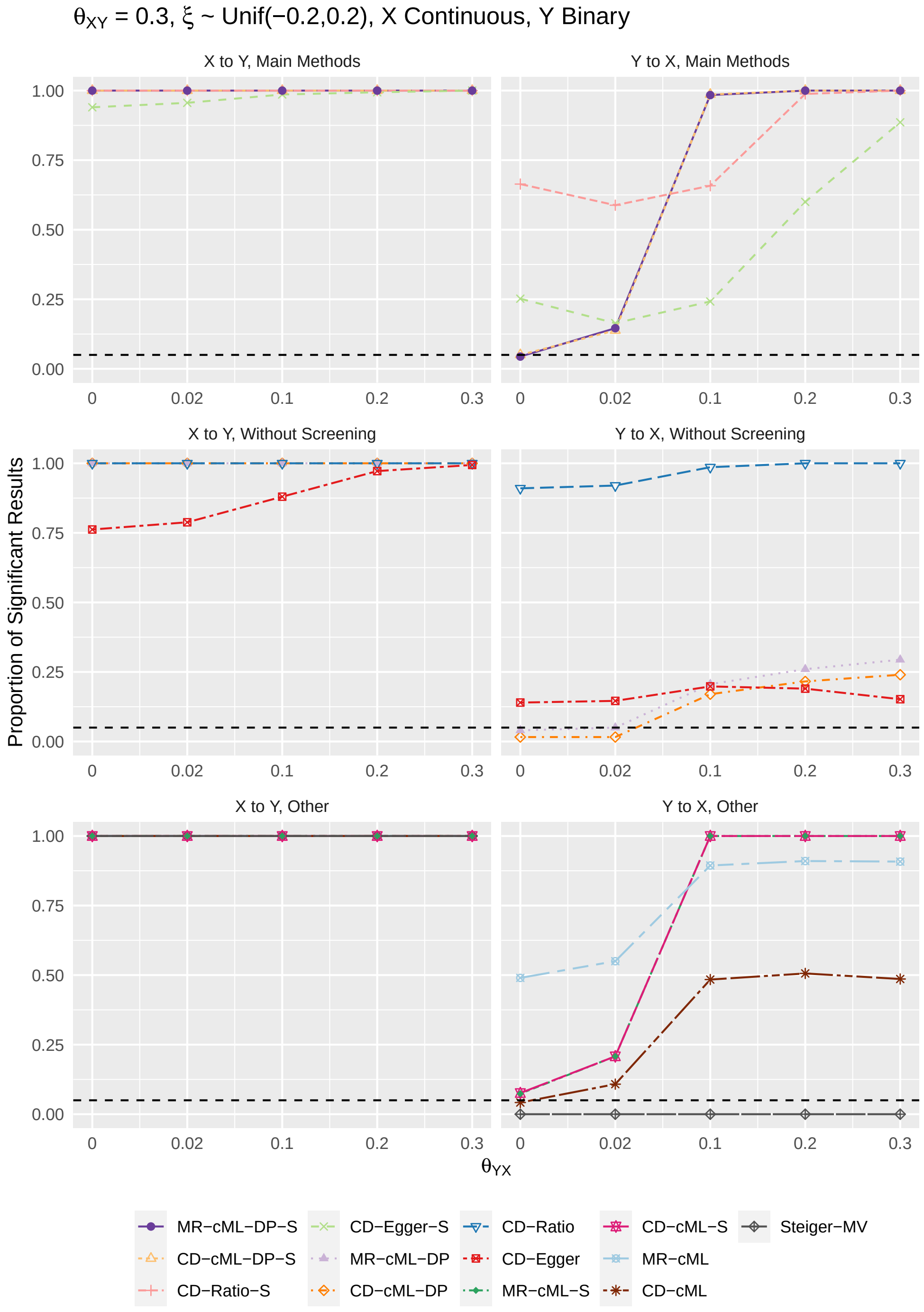

S31 Fig: When both $X$ and $Y$ are binary, $\theta_{XY} = 0$ and $\xi = 0$, the proportions of significant simulation results obtained by the methods for direction $X \to Y$ (left column) and $Y \to X$ (right column). The first row shows results for four main methods: MR-cML-DP-S, CD-cML-DP-S, CD-Ratio-S, and CD-Egger-S; the second row shows results for four methods without screening: MR-cML-DP, CD-cML-DP, CD-Ratio, and CD-Egger; the third row shows results for other five methods.

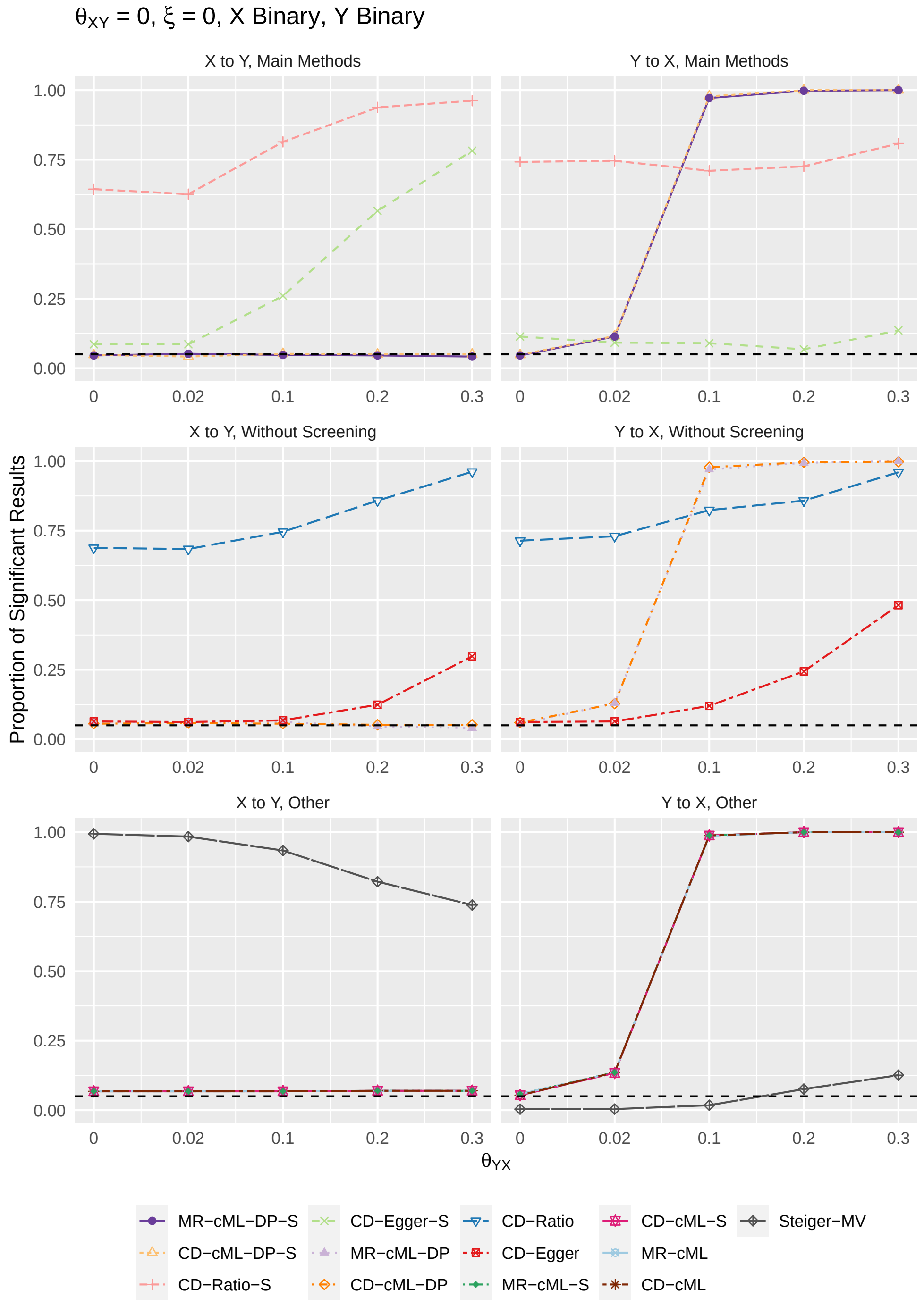

S32 Fig: When both $X$ and $Y$ are binary, $\theta_{XY} = 0$ and $\xi \sim$ Unif(-0.2,0.2), the proportions of significant simulation results obtained by the methods for direction $X \to Y$ (left column) and $Y \to X$ (right column). The first row shows results for four main methods: MR-cML-DP-S, CD-cML-DP-S, CD-Ratio-S, and CD-Egger-S; the second row shows results for four methods without screening: MR-cML-DP, CD-cML-DP, CD-Ratio, and CD-Egger; the third row shows results for other five methods.

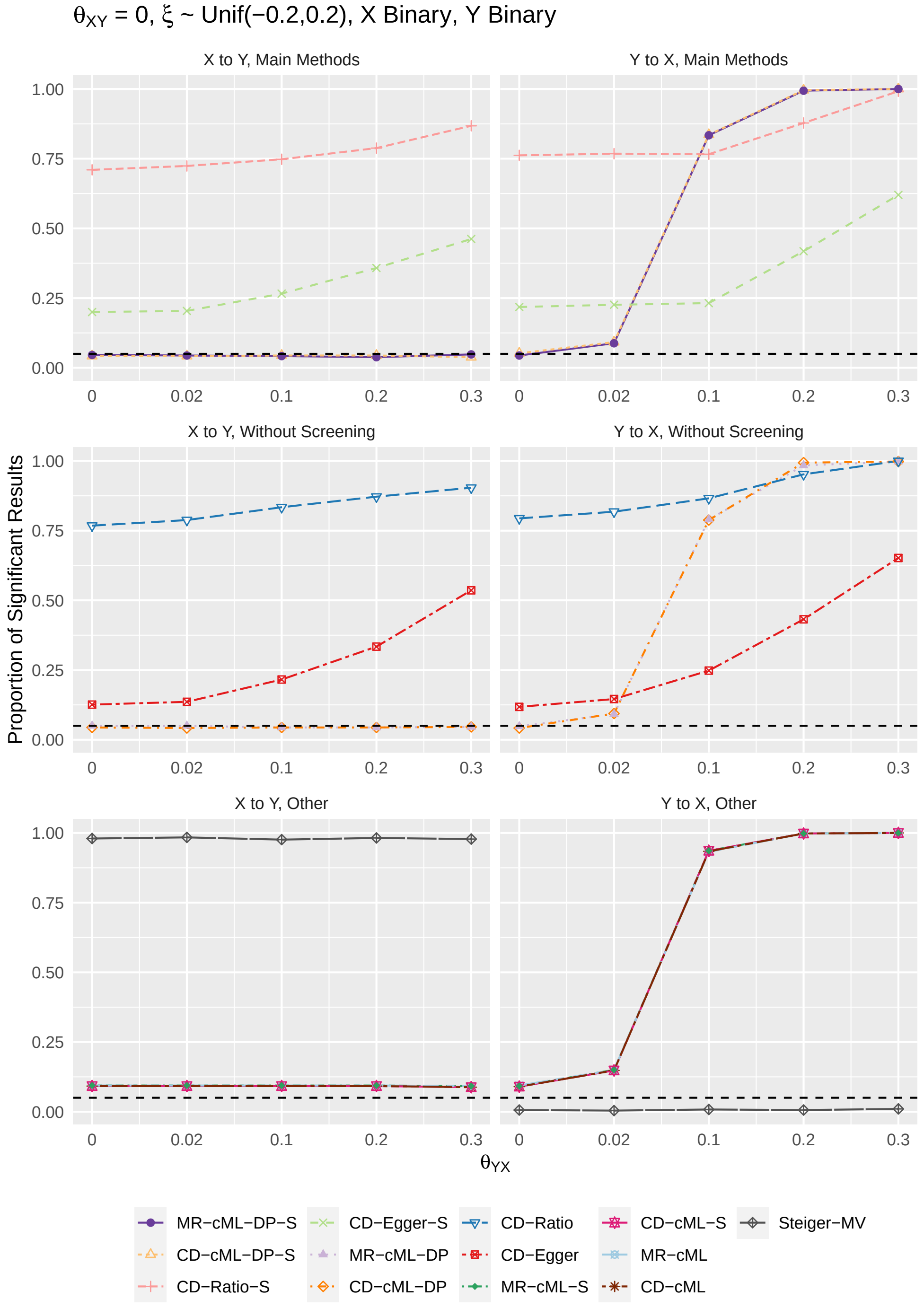

S33 Fig: When both $X$ and $Y$ are binary, $\theta_{XY} = 0.02$ and $\xi = 0$, the proportions of significant simulation results obtained by the methods for direction $X \to Y$ (left column) and $Y \to X$ (right column). The first row shows results for four main methods: MR-cML-DP-S, CD-cML-DP-S, CD-Ratio-S, and CD-Egger-S; the second row shows results for four methods without screening: MR-cML-DP, CD-cML-DP, CD-Ratio, and CD-Egger; the third row shows results for other five methods.

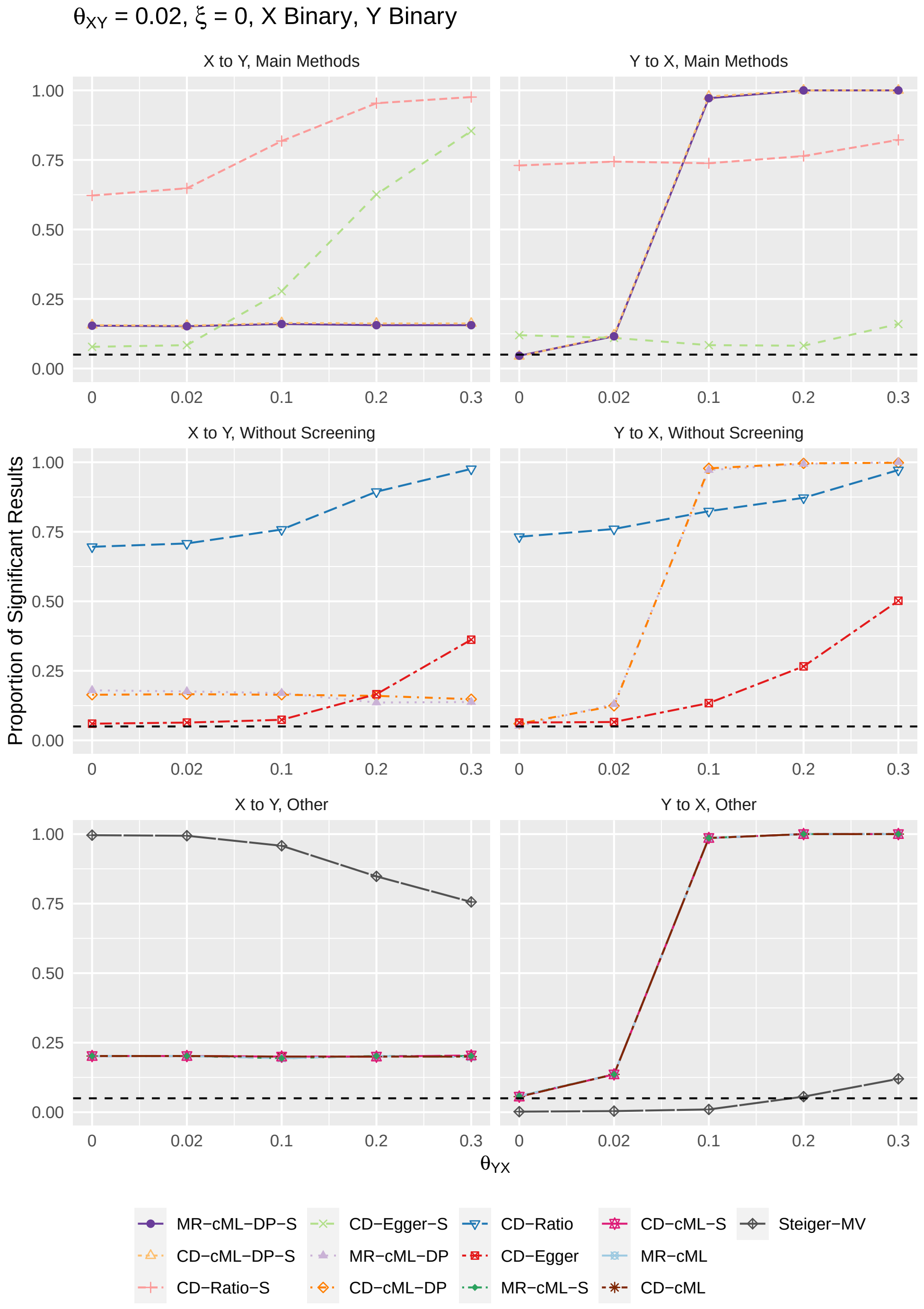

S34 Fig: When both $X$ and $Y$ are binary, $\theta_{XY} = 0.02$ and $\xi \sim$ Unif(-0.2,0.2), the proportions of significant simulation results obtained by the methods for direction $X \to Y$ (left column) and $Y \to X$ (right column). The first row shows results for four main methods: MR-cML-DP-S, CD-cML-DP-S, CD-Ratio-S, and CD-Egger-S; the second row shows results for four methods without screening: MR-cML-DP, CD-cML-DP, CD-Ratio, and CD-Egger; the third row shows results for other five methods.

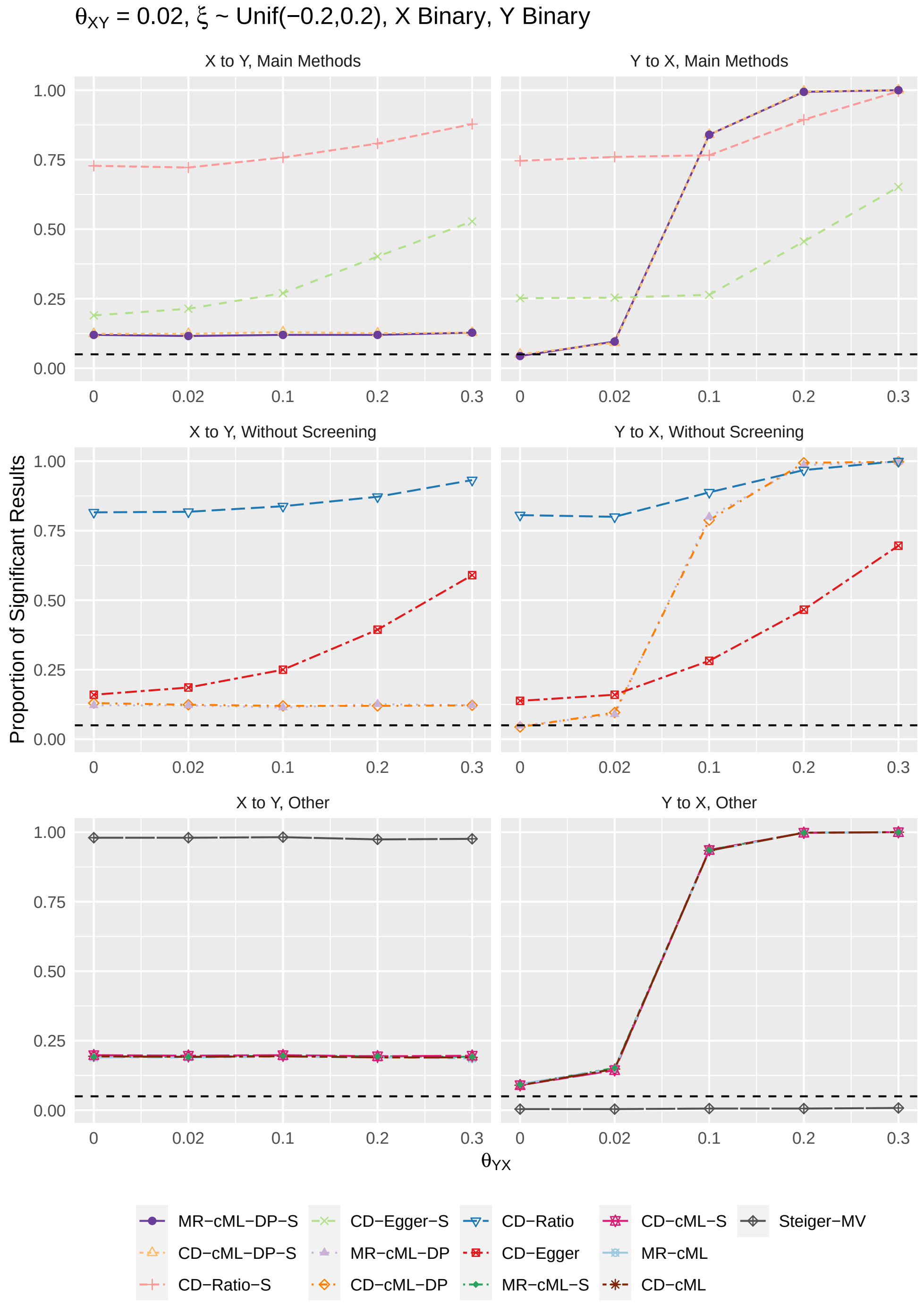

S35 Fig: When both $X$ and $Y$ are binary, $\theta_{XY} = 0.1$ and $\xi = 0$, the proportions of significant simulation results obtained by the methods for direction $X \to Y$ (left column) and $Y \to X$ (right column). The first row shows results for four main methods: MR-cML-DP-S, CD-cML-DP-S, CD-Ratio-S, and CD-Egger-S; the second row shows results for four methods without screening: MR-cML-DP, CD-cML-DP, CD-Ratio, and CD-Egger; the third row shows results for other five methods.

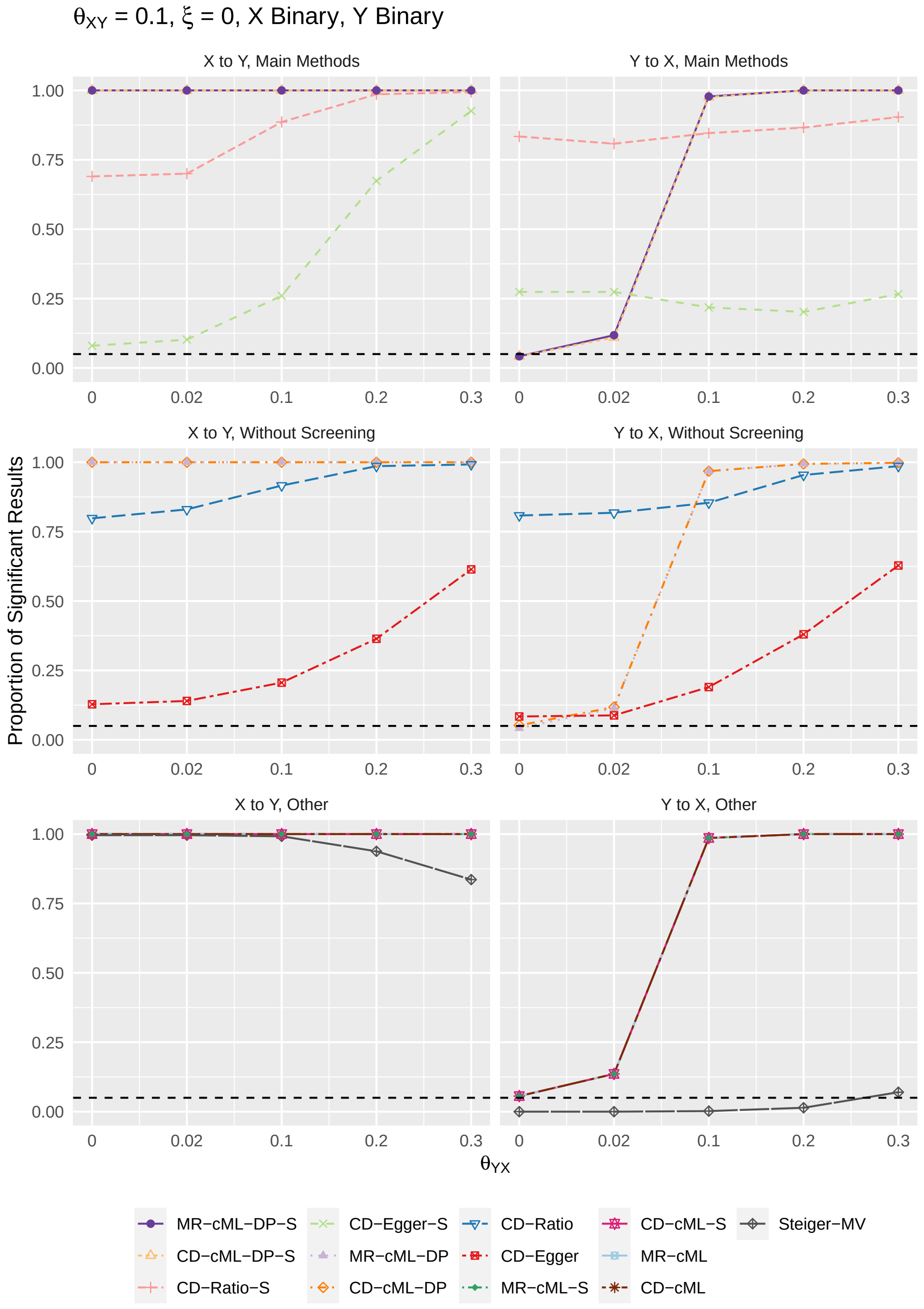

S36 Fig: When both $X$ and $Y$ are binary, $\theta_{XY} = 0.1$ and $\xi \sim$ Unif(-0.2,0.2), the proportions of significant simulation results obtained by the methods for direction $X \to Y$ (left column) and $Y \to X$ (right column). The first row shows results for four main methods: MR-cML-DP-S, CD-cML-DP-S, CD-Ratio-S, and CD-Egger-S; the second row shows results for four methods without screening: MR-cML-DP, CD-cML-DP, CD-Ratio, and CD-Egger; the third row shows results for other five methods.

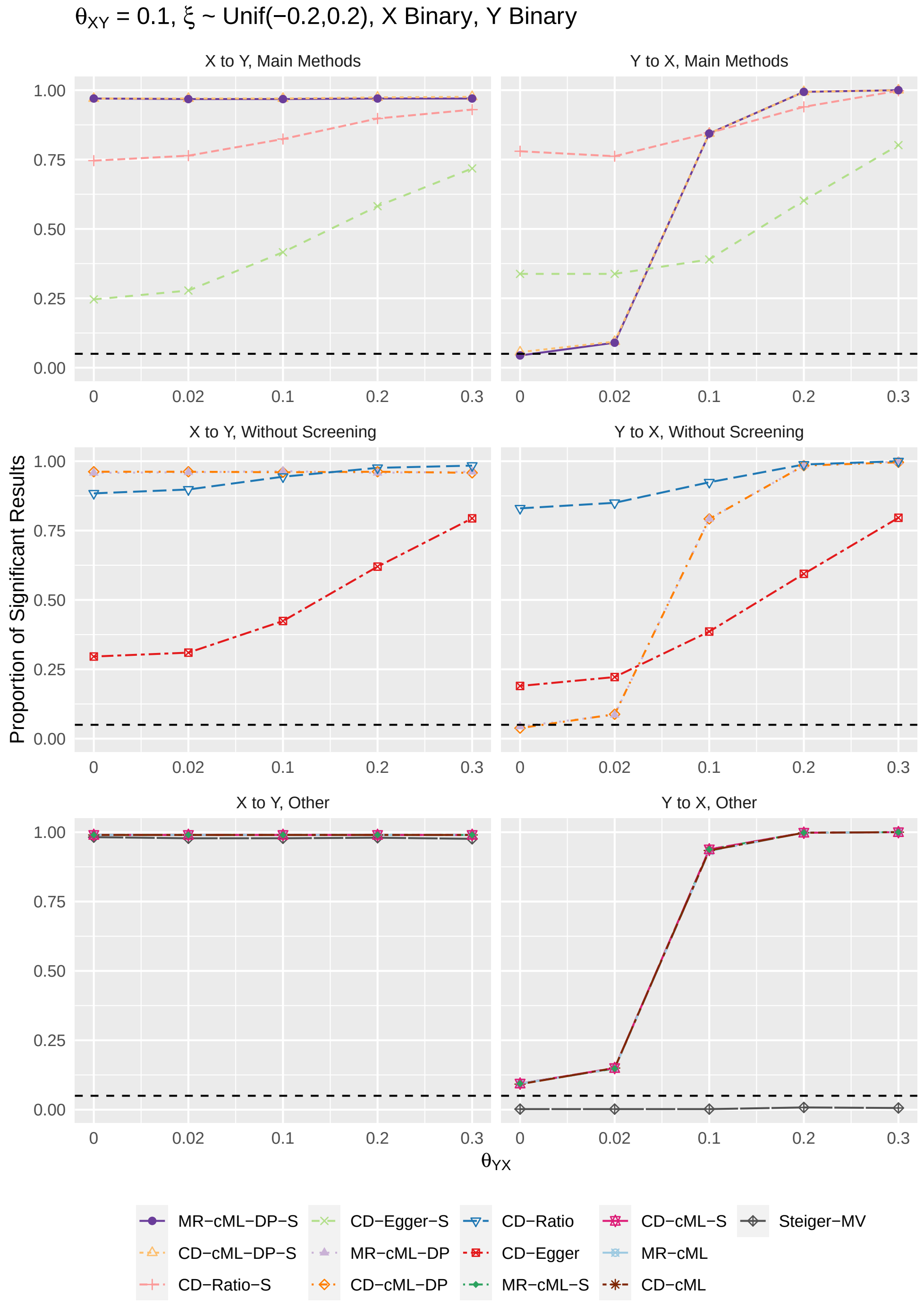

S37 Fig: When both $X$ and $Y$ are binary, $\theta_{XY} = 0.2$ and $\xi = 0$, the proportions of significant simulation results obtained by the methods for direction $X \to Y$ (left column) and $Y \to X$ (right column). The first row shows results for four main methods: MR-cML-DP-S, CD-cML-DP-S, CD-Ratio-S, and CD-Egger-S; the second row shows results for four methods without screening: MR-cML-DP, CD-cML-DP, CD-Ratio, and CD-Egger; the third row shows results for other five methods.

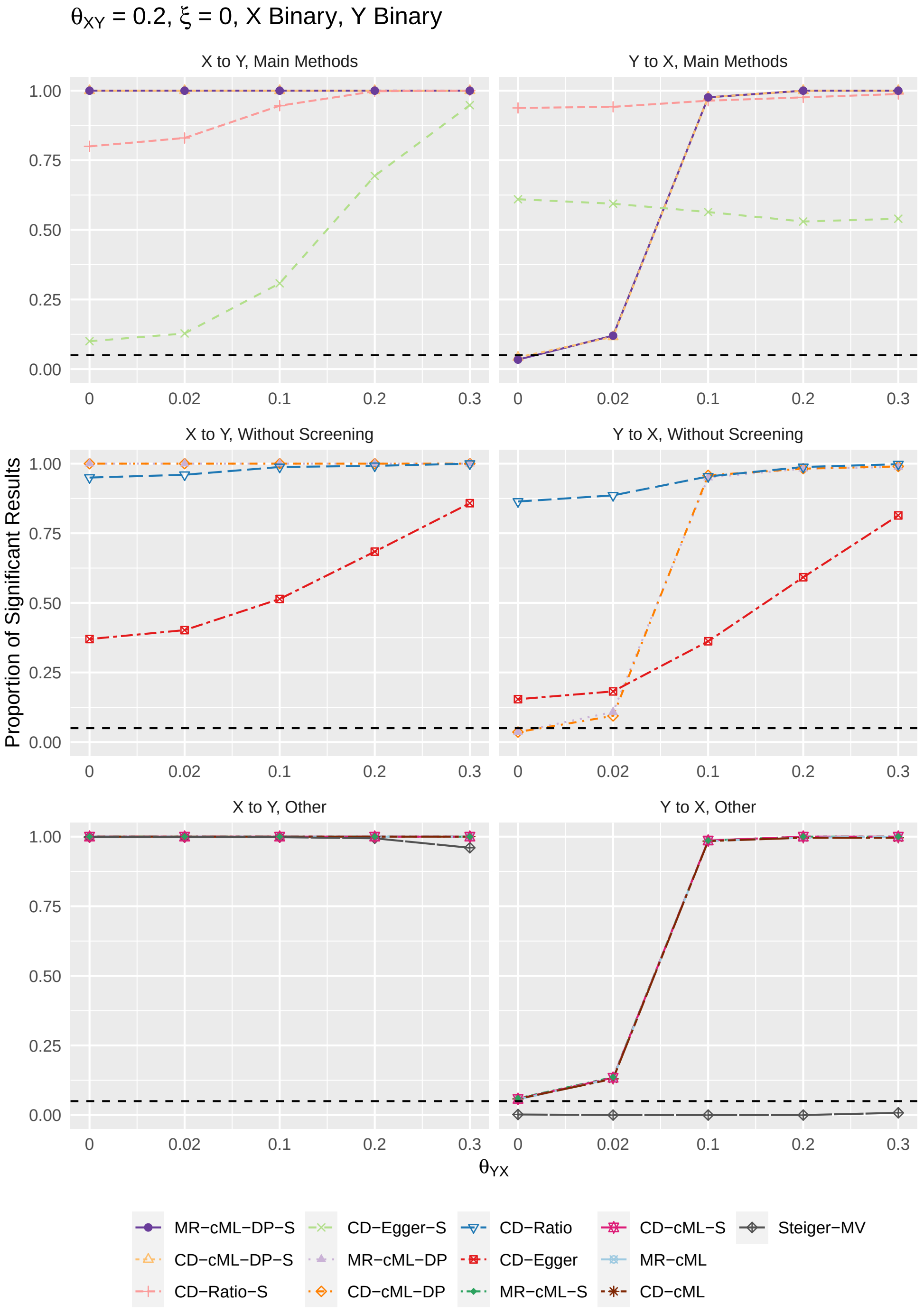

S38 Fig: When both $X$ and $Y$ are binary, $\theta_{XY} = 0.2$ and $\xi \sim$ Unif(-0.2,0.2), the proportions of significant simulation results obtained by the methods for direction $X \to Y$ (left column) and $Y \to X$ (right column). The first row shows results for four main methods: MR-cML-DP-S, CD-cML-DP-S, CD-Ratio-S, and CD-Egger-S; the second row shows results for four methods without screening: MR-cML-DP, CD-cML-DP, CD-Ratio, and CD-Egger; the third row shows results for other five methods.

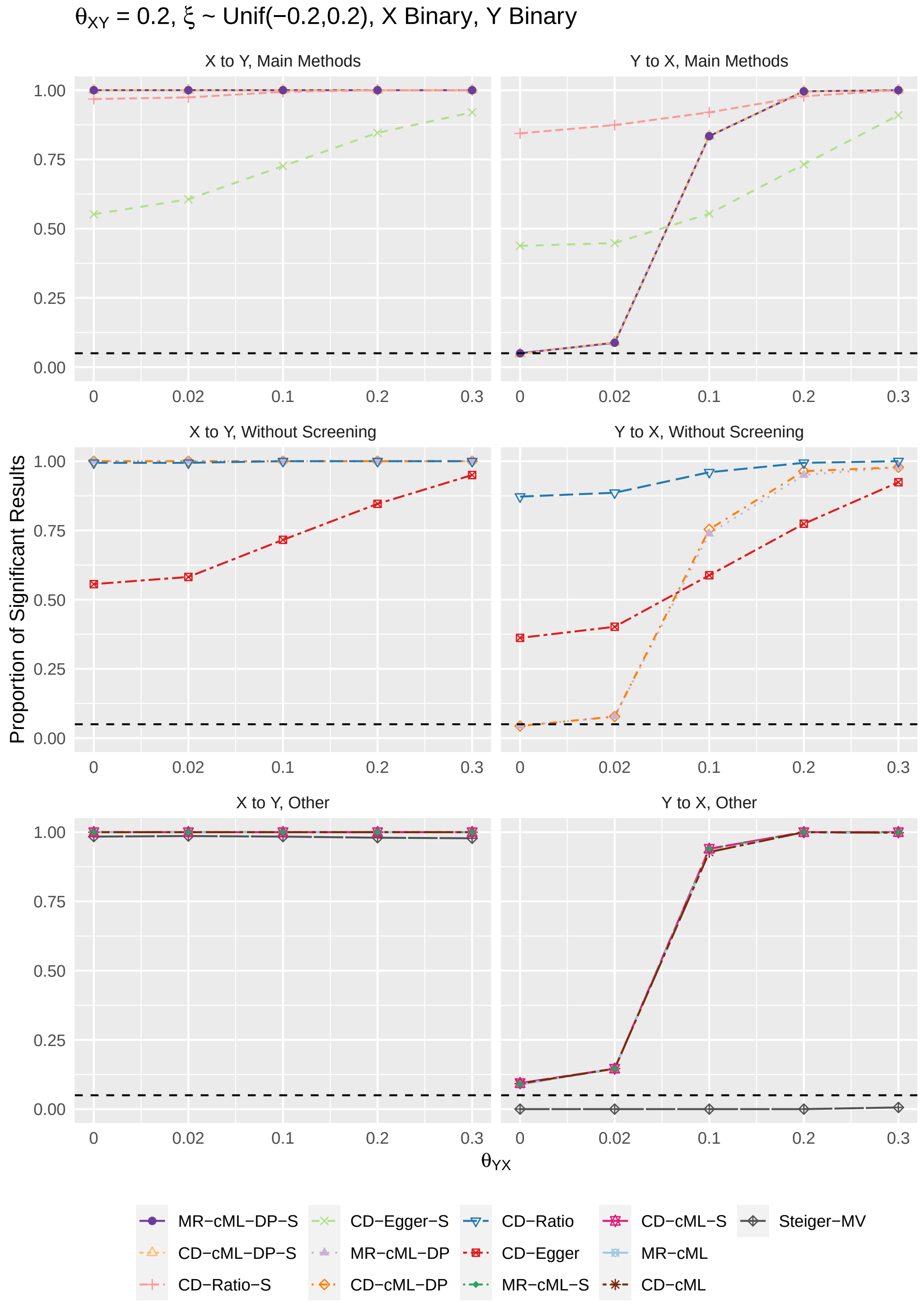

S39 Fig: When both $X$ and $Y$ are binary, $\theta_{XY} = 0.3$ and $\xi = 0$, the proportions of significant simulation results obtained by the methods for direction $X \to Y$ (left column) and $Y \to X$ (right column). The first row shows results for four main methods: MR-cML-DP-S, CD-cML-DP-S, CD-Ratio-S, and CD-Egger-S; the second row shows results for four methods without screening: MR-cML-DP, CD-cML-DP, CD-Ratio, and CD-Egger; the third row shows results for other five methods.

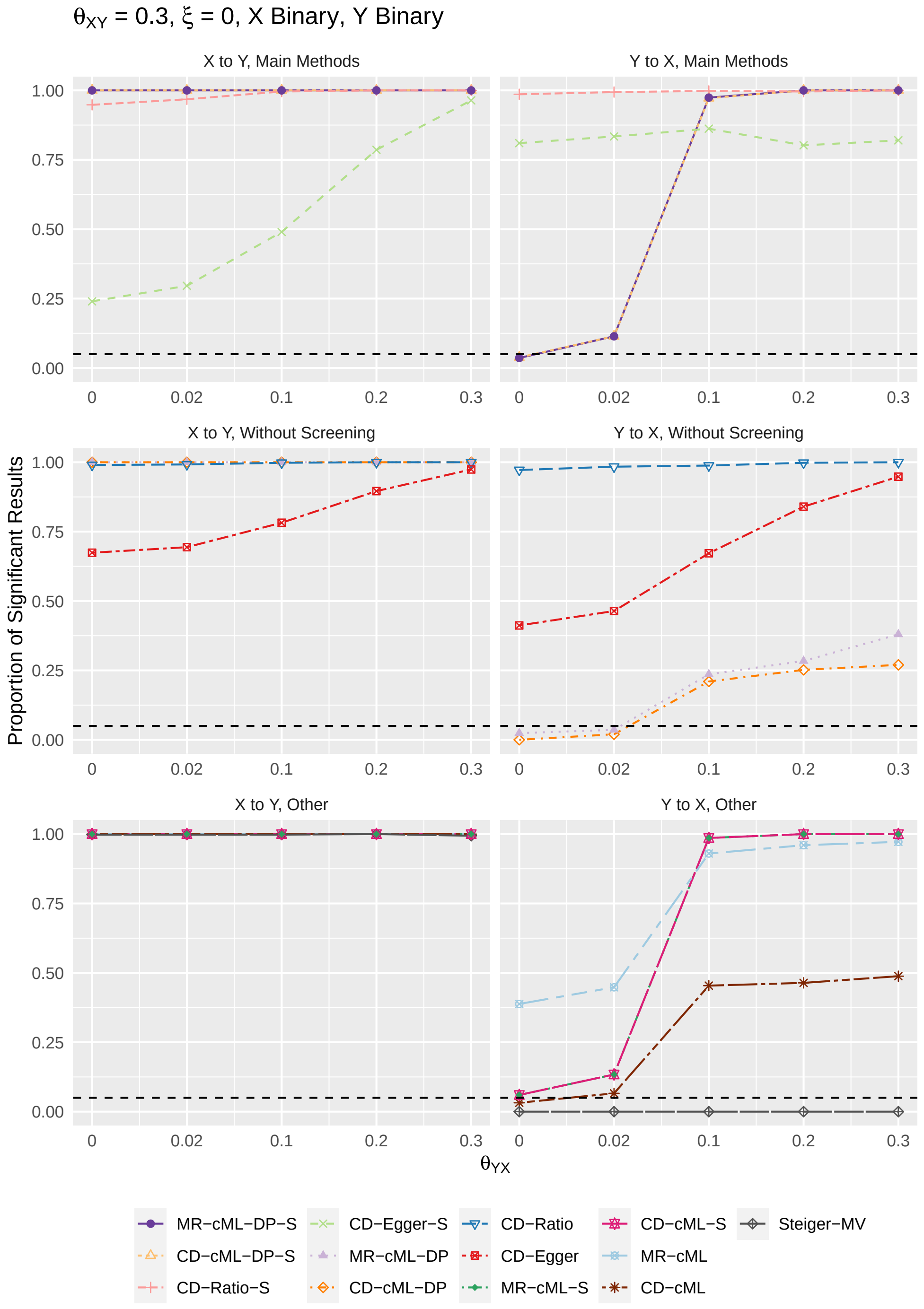

S40 Fig: When both $X$ and $Y$ are binary, $\theta_{XY} = 0.3$ and $\xi \sim$ Unif(-0.2,0.2), the proportions of significant simulation results obtained by the methods for direction $X \to Y$ (left column) and $Y \to X$ (right column). The first row shows results for four main methods: MR-cML-DP-S, CD-cML-DP-S, CD-Ratio-S, and CD-Egger-S; the second row shows results for four methods without screening: MR-cML-DP, CD-cML-DP, CD-Ratio, and CD-Egger; the third row shows results for other five methods.

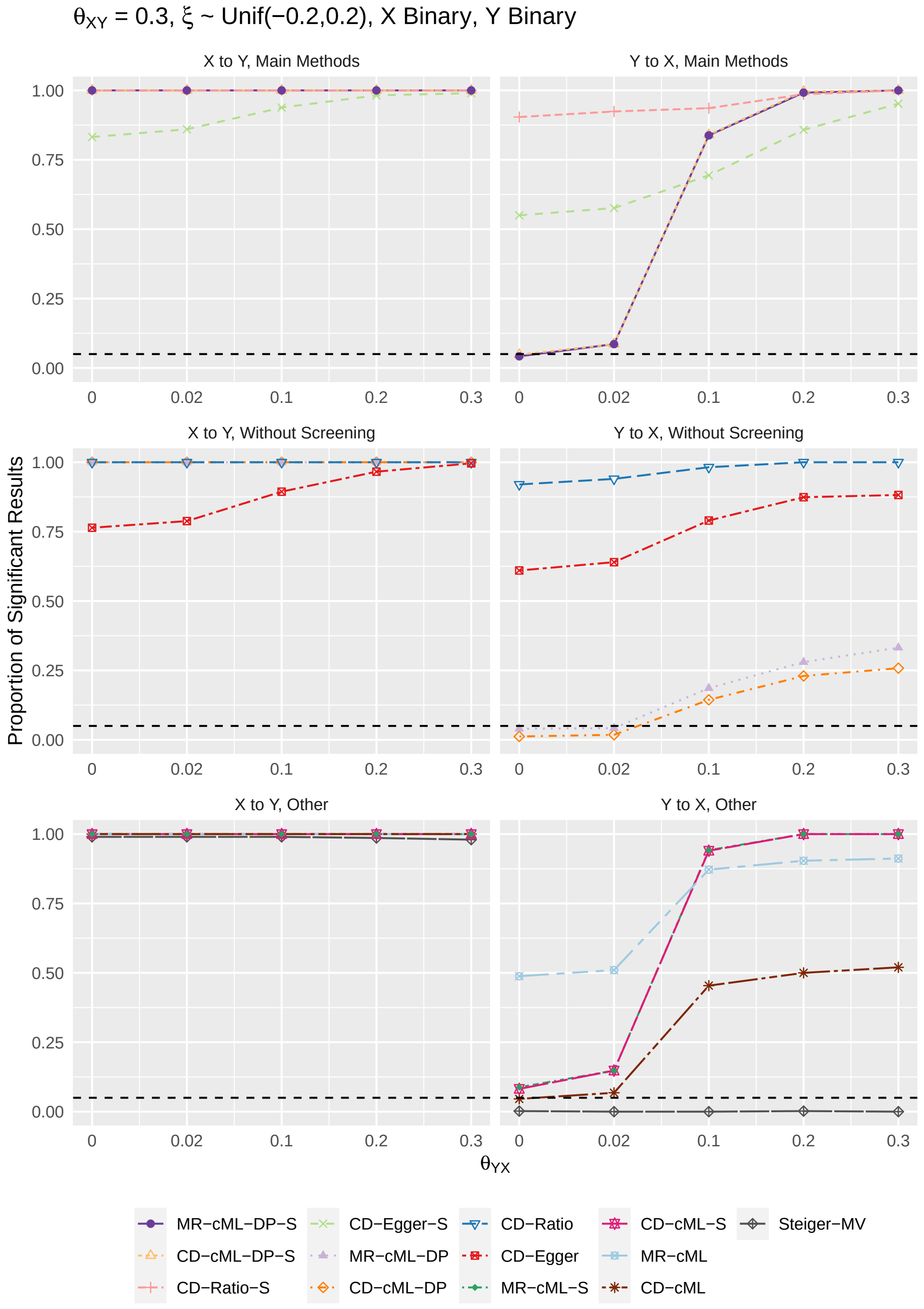

# S2 Full Real Data Results

## S2.1 48 Risk Factor-Disease Pairs

S1 Table: Inferring causal effects between first 6 risk factors and CAD. In each cell we show the Bonferroni adjusted 1-0.05/96 $\approx$ 0.9995 confidence intervals (CIs) of $\theta$ for the MR methods, and CIs of $K$ for the CD methods; for Steiger's method, we show the proportion of SNPs giving significant result. TRUE/FALSE in each cell indicates whether the result is significant or not, and the cells giving significant results are marked in red.

| Method \ Direction | TG to CAD | CAD to TG | LDL to CAD | CAD to LDL | HDL to CAD | CAD to HDL | Height to CAD | CAD to Height | BMI to CAD | CAD to BMI | BF to CAD | CAD to BF |
|---|---|---|---|---|---|---|---|---|---|---|---|---|
| MR-cML-DP-S | (0.122, 0.563), TRUE | (-0.049, 0.079), FALSE | (0.184, 0.67), TRUE | (-0.101, 0.03), FALSE | (-0.557, 0.035), FALSE | (-0.058, 0.055), FALSE | (-0.166, -0.039), TRUE | (-0.021, 0.069), FALSE | (0.139, 0.49), TRUE | (-0.084, 0.003), FALSE | (-0.676, 0.948), FALSE | (-0.104, 0.019), FALSE |
| MR-cML-S | (0.278, 0.461), TRUE | (-0.022, 0.047), FALSE | (0.287, 0.499), TRUE | (-0.076, 0.014), FALSE | (-0.346, -0.179), TRUE | (-0.039, 0.039), FALSE | (-0.151, -0.05), TRUE | (-0.007, 0.053), FALSE | (0.171, 0.426), TRUE | (-0.067, -0.009), TRUE | (-0.159, 0.577), FALSE | (-0.08, 0.004), FALSE |
| CD-cML-DP-S | (0.02, 0.121), TRUE | (-0.157, 0.268), FALSE | (0.053, 0.159), TRUE | (-0.387, 0.116), FALSE | (-0.113, 0.005), FALSE | (-0.193, 0.213), FALSE | (-0.048, -0.011), TRUE | (-0.067, 0.212), FALSE | (0.047, 0.16), TRUE | (-0.205, 0.001), FALSE | (-0.144, 0.223), FALSE | (-0.349, 0.063), FALSE |
| CD-cML-S | (0.055, 0.093), TRUE | (-0.091, 0.187), FALSE | (0.096, 0.135), TRUE | (-0.297, 0.054), FALSE | (-0.074, -0.038), TRUE | (-0.131, 0.152), FALSE | (-0.043, -0.014), TRUE | (-0.033, 0.175), FALSE | (0.057, 0.139), TRUE | (-0.175, -0.022), TRUE | (-0.036, 0.143), FALSE | (-0.269, 0.012), FALSE |
| CD-Ratio-S | (0.046, 0.078), TRUE | (-0.056, 0.211), FALSE | (0.079, 0.106), TRUE | (-0.016, 0.267), FALSE | (-0.052, -0.023), TRUE | (-0.158, 0.115), FALSE | (-0.038, -0.01), TRUE | (-0.068, 0.124), FALSE | (0.052, 0.131), TRUE | (-0.161, -0.014), TRUE | (-0.054, 0.093), FALSE | (-0.253, 0.022), FALSE |
| CD-Egger-S | (0.036, 0.106), TRUE | (-0.146, 0.34), FALSE | (0.061, 0.12), TRUE | (-0.151, 0.513), FALSE | (-0.093, 0.001), FALSE | (-0.282, 0.174), FALSE | (-0.048, -0.004), TRUE | (-0.205, 0.178), FALSE | (0.032, 0.164), TRUE | (-0.213, 0.047), FALSE | (-0.168, 0.144), FALSE | (-0.287, 0.084), FALSE |
| MR-cML-DP | (0.122, 0.563), TRUE | (-0.048, 0.081), FALSE | (0.175, 0.698), TRUE | (-0.098, 0.03), FALSE | (-0.557, 0.035), FALSE | (-0.055, 0.054), FALSE | (-0.166, -0.039), TRUE | (-0.024, 0.074), FALSE | (0.139, 0.49), TRUE | (-0.084, 0.003), FALSE | (-0.676, 0.948), FALSE | (-0.104, 0.019), FALSE |
| MR-cML | (0.278, 0.461), TRUE | (-0.022, 0.047), FALSE | (0.225, 0.584), TRUE | (-0.076, 0.014), FALSE | (-0.346, -0.179), TRUE | (-0.039, 0.039), FALSE | (-0.151, -0.05), TRUE | (-0.007, 0.053), FALSE | (0.171, 0.426), TRUE | (-0.067, -0.009), TRUE | (-0.159, 0.577), FALSE | (-0.08, 0.004), FALSE |
| CD-cML-DP | (0.02, 0.121), TRUE | (-0.158, 0.281), FALSE | (0.053, 0.162), TRUE | (-0.362, 0.105), FALSE | (-0.113, 0.005), FALSE | (-0.176, 0.201), FALSE | (-0.048, -0.011), TRUE | (-0.075, 0.222), FALSE | (0.047, 0.16), TRUE | (-0.205, 0.001), FALSE | (-0.144, 0.223), FALSE | (-0.349, 0.063), FALSE |
| CD-cML | (0.055, 0.093), TRUE | (-0.091, 0.187), FALSE | (0.097, 0.135), TRUE | (-0.297, 0.054), FALSE | (-0.074, -0.038), TRUE | (-0.131, 0.152), FALSE | (-0.043, -0.014), TRUE | (-0.033, 0.175), FALSE | (0.057, 0.139), TRUE | (-0.175, -0.022), TRUE | (-0.036, 0.143), FALSE | (-0.269, 0.012), FALSE |
| CD-Ratio | (0.046, 0.078), TRUE | (-0.016, 0.25), FALSE | (0.081, 0.108), TRUE | (0.049, 0.327), TRUE | (-0.052, -0.023), TRUE | (-0.303, -0.04), TRUE | (-0.038, -0.01), TRUE | (-0.079, 0.112), FALSE | (0.052, 0.131), TRUE | (-0.161, -0.014), TRUE | (-0.054, 0.093), FALSE | (-0.253, 0.022), FALSE |
| CD-Egger | (0.036, 0.106), TRUE | (-0.156, 0.861), FALSE | (0.053, 0.152), TRUE | (-0.106, 2.214), FALSE | (-0.093, 0.001), FALSE | (-1.042, 0.144), FALSE | (-0.048, -0.004), TRUE | (-0.206, 0.201), FALSE | (0.032, 0.164), TRUE | (-0.213, 0.047), FALSE | (-0.168, 0.144), FALSE | (-0.287, 0.084), FALSE |
| LHC-MR | (0.052, 0.202), TRUE | (0.197, 0.726), TRUE | (0.089, 0.23), TRUE | (-0.333, 0.565), FALSE | (-0.184, -0.077), TRUE | (-0.643, -0.098), TRUE | (-0.094, -0.012), TRUE | (-0.498, 0.766), FALSE | (0.103, 0.258), TRUE | (-0.841, 0.621), FALSE | (0.106, 0.278), TRUE | (-0.29, 0.538), FALSE |
| Steiger | 0.505, TRUE | 0.184, FALSE | 0.608, TRUE | 0.128, FALSE | 0.617, TRUE | 0.141, FALSE | 0.914, TRUE | 0.073, FALSE | 0.492, TRUE | 0.407, FALSE | 0.111, FALSE | 0.25, FALSE |

S2 Table: Inferring causal effects between second 6 risk factors and CAD. In each cell we show the Bonferroni adjusted 1-0.05/96 $\approx$ 0.9995 confidence intervals (CIs) of $\theta$ for the MR methods, and CIs of $K$ for the CD methods; for Steiger's method, we show the proportion of SNPs giving significant result. TRUE/FALSE in each cell indicates whether the result is significant or not, and the cells giving significant results are marked in red.

| Method \ Direction | BW to CAD | CAD to BW | DBP to CAD | CAD to DBP | SBP to CAD | CAD to SBP | FG to CAD | CAD to FG | Smoke to CAD | CAD to Smoke | Alcohol to CAD | CAD to Alcohol |
|---|---|---|---|---|---|---|---|---|---|---|---|---|
| MR-cML-DP-S | (-0.37, 0.152), FALSE | (-0.063, 0.027), FALSE | (0.055, 0.087), TRUE | (-0.978, 1.072), FALSE | (0.037, 0.053), TRUE | (-1.501, 4.369), FALSE | (0.126, 0.557), TRUE | (-0.028, 0.05), FALSE | (-0.166, 0.378), FALSE | (-0.072, 0.037), FALSE | (-0.212, 0.78), FALSE | (-0.014, 0.023), FALSE |
| MR-cML-S | (-0.263, 0.027), FALSE | (-0.05, 0.009), FALSE | (0.058, 0.075), TRUE | (-0.248, 0.147), FALSE | (0.04, 0.05), TRUE | (-0.345, 4.369), FALSE | (0.139, 0.547), TRUE | (-0.019, 0.04), FALSE | (-0.092, 0.311), FALSE | (-0.058, 0.026), FALSE | (0.017, 0.558), TRUE | (-0.01, 0.019), FALSE |
| CD-cML-DP-S | (-0.102, 0.043), FALSE | (-0.23, 0.098), FALSE | (0.16, 0.252), TRUE | (-0.328, 0.361), FALSE | (0.183, 0.265), TRUE | (-0.302, 0.862), FALSE | (0.019, 0.087), TRUE | (-0.187, 0.306), FALSE | (-0.089, 0.204), FALSE | (-0.132, 0.069), FALSE | (-0.06, 0.219), FALSE | (-0.053, 0.086), FALSE |
| CD-cML-S | (-0.072, 0.008), FALSE | (-0.182, 0.034), FALSE | (0.168, 0.217), TRUE | (-0.083, 0.056), FALSE | (0.198, 0.248), TRUE | (-0.273, 0.973), FALSE | (0.021, 0.085), TRUE | (-0.133, 0.243), FALSE | (-0.049, 0.168), FALSE | (-0.105, 0.05), FALSE | (0.004, 0.156), TRUE | (-0.038, 0.069), FALSE |
| CD-Ratio-S | (-0.066, 0.009), FALSE | (-0.181, 0.024), FALSE | (0.15, 0.195), TRUE | (-0.022, 0.088), FALSE | (0.171, 0.217), TRUE | (0.069, 0.176), TRUE | (0.012, 0.075), TRUE | (-0.135, 0.237), FALSE | (-0.027, 0.079), FALSE | (-0.094, 0.055), FALSE | (-0.018, 0.122), FALSE | (-0.03, 0.072), FALSE |
| CD-Egger-S | (-0.118, 0.038), FALSE | (-0.245, 0.057), FALSE | (0.142, 0.226), TRUE | (-0.183, 0.25), FALSE | (0.167, 0.251), TRUE | (-0.042, 0.327), FALSE | (-0.01, 0.108), FALSE | (-0.188, 0.291), FALSE | (-0.044, 0.114), FALSE | (-0.136, 0.12), FALSE | (-0.098, 0.187), FALSE | (-0.052, 0.094), FALSE |
| MR-cML-DP | (-0.37, 0.152), FALSE | (-0.065, 0.031), FALSE | (0.054, 0.088), TRUE | (-2.028, 2.379), FALSE | (0.036, 0.053), TRUE | (-2.254, 5.004), FALSE | (0.126, 0.557), TRUE | (-0.028, 0.05), FALSE | (-0.166, 0.378), FALSE | (-0.072, 0.037), FALSE | (-0.212, 0.78), FALSE | (-0.014, 0.023), FALSE |
| MR-cML | (-0.263, 0.027), FALSE | (-0.05, 0.009), FALSE | (0.058, 0.075), TRUE | (-0.248, 0.148), FALSE | (0.04, 0.05), TRUE | (1.914, 3.044), TRUE | (0.139, 0.547), TRUE | (-0.019, 0.04), FALSE | (-0.092, 0.311), FALSE | (-0.058, 0.026), FALSE | (0.017, 0.558), TRUE | (-0.01, 0.019), FALSE |
| CD-cML-DP | (-0.102, 0.043), FALSE | (-0.233, 0.113), FALSE | (0.159, 0.254), TRUE | (-0.635, 0.728), FALSE | (0.184, 0.263), TRUE | (-0.462, 1.001), FALSE | (0.019, 0.087), TRUE | (-0.187, 0.306), FALSE | (-0.089, 0.204), FALSE | (-0.132, 0.069), FALSE | (-0.06, 0.219), FALSE | (-0.053, 0.086), FALSE |
| CD-cML | (-0.072, 0.008), FALSE | (-0.182, 0.034), FALSE | (0.168, 0.217), TRUE | (-0.083, 0.056), FALSE | (0.198, 0.248), TRUE | (0.35, 0.62), TRUE | (0.021, 0.085), TRUE | (-0.133, 0.243), FALSE | (-0.049, 0.168), FALSE | (-0.105, 0.05), FALSE | (0.004, 0.156), TRUE | (-0.038, 0.069), FALSE |
| CD-Ratio | (-0.066, 0.009), FALSE | (-0.196, 0.008), FALSE | (0.151, 0.196), TRUE | (0.03, 0.137), TRUE | (0.173, 0.218), TRUE | (0.12, 0.225), TRUE | (0.012, 0.075), TRUE | (-0.135, 0.237), FALSE | (-0.027, 0.079), FALSE | (-0.094, 0.055), FALSE | (-0.018, 0.122), FALSE | (-0.03, 0.072), FALSE |
| CD-Egger | (-0.118, 0.038), FALSE | (-0.28, 0.049), FALSE | (0.142, 0.231), TRUE | (-0.189, 0.696), FALSE | (0.163, 0.258), TRUE | (0.038, 0.656), TRUE | (-0.01, 0.108), FALSE | (-0.188, 0.291), FALSE | (-0.044, 0.114), FALSE | (-0.136, 0.12), FALSE | (-0.098, 0.187), FALSE | (-0.052, 0.094), FALSE |
| LHC-MR | (-0.275, 0.065), FALSE | (-0.933, 0.353), FALSE | (0.171, 0.307), TRUE | (-1.115, 0.292), FALSE | (0.146, 0.385), TRUE | (-2.152, 1.499), FALSE | (0.029, 0.168), TRUE | (-0.715, -0.025), TRUE | (0.069, 0.3), TRUE | (0.057, 0.469), TRUE | (-0.261, 0.095), FALSE | (-0.219, 0.296), FALSE |
| Steiger | 0.407, TRUE | 0.296, FALSE | 0.833, TRUE | 0.055, FALSE | 0.809, TRUE | 0.071, FALSE | 0.167, FALSE | 0.097, FALSE | 0.209, FALSE | 0.64, TRUE | 0.27, FALSE | 0.69, TRUE |

S3 Table: Inferring causal effects between first 6 risk factors and Stroke. In each cell we show the Bonferroni adjusted 1-0.05/96 ≈ 0.9995 confidence intervals (CIs) of $\theta$ for the MR methods, and CIs of $K$ for the CD methods; for Steiger's method, we show the proportion of SNPs giving significant result. TRUE/FALSE in each cell indicates whether the result is significant or not, and the cells giving significant results are marked in red.

| Method \ Direction | TG to Stroke | Stroke to TG | LDL to Stroke | Stroke to LDL | HDL to Stroke | Stroke to HDL | Height to Stroke | Stroke to Height | BMI to Stroke | Stroke to BMI | BF to Stroke | Stroke to BF |
|---|---|---|---|---|---|---|---|---|---|---|---|---|
| MR-cML-DP-S | (-0.109, 0.116), FALSE | (-0.029, 0.179), FALSE | (0.008, 0.2), TRUE | (-0.188, 0.14), FALSE | (-0.154, 0.029), FALSE | (-0.107, 0.111), FALSE | (-0.091, 0.062), FALSE | (-0.233, 0.137), FALSE | (-0.014, 0.299), FALSE | (-0.112, 0.11), FALSE | (-0.216, 0.45), FALSE | (-0.098, 0.104), FALSE |
| MR-cML-S | (-0.088, 0.088), FALSE | (-0.009, 0.155), FALSE | (0.044, 0.178), TRUE | (-0.141, 0.088), FALSE | (-0.138, 0.02), FALSE | (-0.087, 0.095), FALSE | (-0.069, 0.045), FALSE | (-0.161, 0.034), FALSE | (0.01, 0.293), TRUE | (-0.085, 0.085), FALSE | (-0.189, 0.399), FALSE | (-0.095, 0.093), FALSE |
| CD-cML-DP-S | (-0.022, 0.023), FALSE | (-0.15, 0.67), FALSE | (0.003, 0.045), TRUE | (-0.598, 0.431), FALSE | (-0.032, 0.005), FALSE | (-0.355, 0.444), FALSE | (-0.026, 0.018), FALSE | (-0.862, 0.499), FALSE | (-0.007, 0.094), FALSE | (-0.252, 0.266), FALSE | (-0.055, 0.115), FALSE | (-0.336, 0.367), FALSE |
| CD-cML-S | (-0.018, 0.017), FALSE | (-0.082, 0.601), FALSE | (0.01, 0.039), TRUE | (-0.456, 0.292), FALSE | (-0.028, 0.004), FALSE | (-0.293, 0.399), FALSE | (-0.019, 0.013), FALSE | (-0.597, 0.134), FALSE | (0.001, 0.091), TRUE | (-0.205, 0.22), FALSE | (-0.047, 0.102), FALSE | (-0.328, 0.332), FALSE |
| CD-Ratio-S | (-0.017, 0.018), FALSE | (-0.062, 0.562), FALSE | (0.008, 0.037), TRUE | (-0.359, 0.289), FALSE | (-0.03, 0.002), FALSE | (-0.295, 0.392), FALSE | (-0.024, 0.007), FALSE | (-0.387, 0.243), FALSE | (-0.002, 0.085), FALSE | (-0.243, 0.152), FALSE | (-0.048, 0.101), FALSE | (-0.325, 0.329), FALSE |
| CD-Egger-S | (-0.023, 0.025), FALSE | (-0.123, 0.677), FALSE | (0, 0.046), FALSE | (-0.476, 0.473), FALSE | (-0.039, 0.006), FALSE | (-0.357, 0.475), FALSE | (-0.03, 0.012), FALSE | (-0.765, 0.48), FALSE | (-0.021, 0.104), FALSE | (-0.387, 0.238), FALSE | (-0.051, 0.101), FALSE | (-0.316, 0.33), FALSE |
| MR-cML-DP | (-0.109, 0.116), FALSE | (-0.029, 0.179), FALSE | (0.008, 0.2), TRUE | (-0.188, 0.137), FALSE | (-0.154, 0.029), FALSE | (-0.117, 0.135), FALSE | (-0.091, 0.062), FALSE | (-0.212, 0.12), FALSE | (-0.014, 0.299), FALSE | (-0.112, 0.11), FALSE | (-0.216, 0.45), FALSE | (-0.098, 0.104), FALSE |
| MR-cML | (-0.088, 0.088), FALSE | (-0.009, 0.155), FALSE | (0.044, 0.178), TRUE | (-0.141, 0.088), FALSE | (-0.138, 0.02), FALSE | (-0.087, 0.095), FALSE | (-0.069, 0.045), FALSE | (-0.161, 0.034), FALSE | (0.01, 0.293), TRUE | (-0.085, 0.085), FALSE | (-0.189, 0.399), FALSE | (-0.095, 0.093), FALSE |
| CD-cML-DP | (-0.022, 0.023), FALSE | (-0.15, 0.67), FALSE | (0.003, 0.045), TRUE | (-0.576, 0.409), FALSE | (-0.032, 0.005), FALSE | (-0.393, 0.53), FALSE | (-0.026, 0.018), FALSE | (-0.787, 0.451), FALSE | (-0.007, 0.094), FALSE | (-0.252, 0.266), FALSE | (-0.055, 0.115), FALSE | (-0.336, 0.367), FALSE |
| CD-cML | (-0.018, 0.017), FALSE | (-0.082, 0.601), FALSE | (0.01, 0.039), TRUE | (-0.456, 0.292), FALSE | (-0.028, 0.004), FALSE | (-0.293, 0.399), FALSE | (-0.019, 0.013), FALSE | (-0.597, 0.134), FALSE | (0.001, 0.091), TRUE | (-0.205, 0.22), FALSE | (-0.047, 0.102), FALSE | (-0.328, 0.332), FALSE |
| CD-Ratio | (-0.017, 0.018), FALSE | (-0.062, 0.562), FALSE | (0.008, 0.037), TRUE | (-0.441, 0.194), FALSE | (-0.03, 0.002), FALSE | (-0.389, 0.283), FALSE | (-0.024, 0.007), FALSE | (-0.438, 0.189), FALSE | (-0.002, 0.085), FALSE | (-0.243, 0.152), FALSE | (-0.048, 0.101), FALSE | (-0.325, 0.329), FALSE |
| CD-Egger | (-0.023, 0.025), FALSE | (-0.123, 0.677), FALSE | (0, 0.046), FALSE | (-0.902, 0.45), FALSE | (-0.039, 0.006), FALSE | (-0.941, 0.553), FALSE | (-0.03, 0.012), FALSE | (-2.199, 0.934), FALSE | (-0.021, 0.104), FALSE | (-0.387, 0.238), FALSE | (-0.051, 0.101), FALSE | (-0.316, 0.33), FALSE |
| LHC-MR | (-0.009, 0.089), FALSE | (0.142, 0.999), TRUE | (0.02, 0.148), TRUE | (-0.666, 0.008), FALSE | (-0.089, 0.005), FALSE | (-0.948, -0.175), TRUE | (-0.011, 0.056), FALSE | (-2.448, 1.135), FALSE | (-0.059, 0.144), FALSE | (-0.073, 1.304), FALSE | (-0.035, 0.333), FALSE | (-0.574, 0.24), FALSE |
| Steiger | 0.786, TRUE | 0.086, FALSE | 0.846, TRUE | 0.066, FALSE | 0.879, TRUE | 0.061, FALSE | 0.984, TRUE | 0.011, FALSE | 0.829, TRUE | 0.132, FALSE | 0.4, TRUE | 0.32, FALSE |

S4 Table: Inferring causal effects between second 6 risk factors and Stroke. In each cell we show the Bonferroni adjusted 1-0.05/96 ≈ 0.9995 confidence intervals (CIs) of $\theta$ for the MR methods, and CIs of $K$ for the CD methods; for Steiger's method, we show the proportion of SNPs giving significant result. TRUE/FALSE in each cell indicates whether the result is significant or not, and the cells giving significant results are marked in red.

| Method \ Direction | BW to Stroke | Stroke to BW | DBP to Stroke | Stroke to DBP | SBP to Stroke | Stroke to SBP | FG to Stroke | Stroke to FG | Smoke to Stroke | Stroke to Smoke | Alcohol to Stroke | Stroke to Alcohol |
|---|---|---|---|---|---|---|---|---|---|---|---|---|
| MR-cML-DP-S | (-0.287, 0.25), FALSE | (-0.163, 0.088), FALSE | (0.042, 0.069), TRUE | (-1.311, -0.019), TRUE | (0.03, 0.045), TRUE | (-1.638, 5.285), FALSE | (-0.279, 0.66), FALSE | (-0.049, 0.114), FALSE | (-0.103, 0.239), FALSE | (-0.108, 0.149), FALSE | (-0.063, 0.514), FALSE | (-0.038, 0.033), FALSE |
| MR-cML-S | (-0.208, 0.146), FALSE | (-0.103, 0.05), FALSE | (0.045, 0.064), TRUE | (-1.236, -0.104), TRUE | (0.031, 0.042), TRUE | (-0.119, 4.224), FALSE | (-0.083, 0.422), FALSE | (-0.034, 0.105), FALSE | (-0.045, 0.197), FALSE | (-0.086, 0.105), FALSE | (-0.029, 0.474), FALSE | (-0.034, 0.03), FALSE |
| CD-cML-DP-S | (-0.074, 0.062), FALSE | (-0.594, 0.328), FALSE | (0.116, 0.187), TRUE | (-0.489, -0.007), TRUE | (0.14, 0.212), TRUE | (-0.404, 1.127), FALSE | (-0.045, 0.099), FALSE | (-0.343, 0.741), FALSE | (-0.049, 0.113), FALSE | (-0.187, 0.293), FALSE | (-0.025, 0.164), FALSE | (-0.147, 0.124), FALSE |
| CD-cML-S | (-0.054, 0.036), FALSE | (-0.371, 0.188), FALSE | (0.127, 0.177), TRUE | (-0.46, -0.038), TRUE | (0.147, 0.198), TRUE | (-0.099, 0.906), FALSE | (-0.017, 0.075), FALSE | (-0.24, 0.684), FALSE | (-0.022, 0.094), FALSE | (-0.153, 0.217), FALSE | (-0.011, 0.145), FALSE | (-0.133, 0.114), FALSE |
| CD-Ratio-S | (-0.042, 0.039), FALSE | (-0.387, 0.105), FALSE | (0.114, 0.162), TRUE | (-0.208, 0.152), FALSE | (0.138, 0.187), TRUE | (-0.019, 0.356), FALSE | (-0.014, 0.054), FALSE | (-0.258, 0.654), FALSE | (-0.026, 0.087), FALSE | (-0.149, 0.21), FALSE | (-0.015, 0.137), FALSE | (-0.133, 0.114), FALSE |
| CD-Egger-S | (-0.063, 0.062), FALSE | (-0.581, 0.187), FALSE | (0.108, 0.18), TRUE | (-0.465, 0.708), FALSE | (0.132, 0.206), TRUE | (-0.314, 0.704), FALSE | (-0.026, 0.088), FALSE | (-0.285, 0.714), FALSE | (-0.052, 0.125), FALSE | (-0.235, 0.262), FALSE | (-0.056, 0.17), FALSE | (-0.13, 0.115), FALSE |
| MR-cML-DP | (-0.287, 0.25), FALSE | (-0.163, 0.088), FALSE | (0.042, 0.069), TRUE | (-1.371, 0.112), FALSE | (0.029, 0.044), TRUE | (4.192, 11.969), TRUE | (-0.279, 0.66), FALSE | (-0.049, 0.114), FALSE | (-0.103, 0.239), FALSE | (-0.108, 0.149), FALSE | (-0.063, 0.514), FALSE | (-0.038, 0.033), FALSE |
| MR-cML | (-0.208, 0.146), FALSE | (-0.103, 0.05), FALSE | (0.045, 0.064), TRUE | (-1.236, -0.103), TRUE | (0.031, 0.042), TRUE | (5.885, 10.424), TRUE | (-0.083, 0.422), FALSE | (-0.034, 0.105), FALSE | (-0.045, 0.197), FALSE | (-0.086, 0.105), FALSE | (-0.029, 0.474), FALSE | (-0.034, 0.03), FALSE |
| CD-cML-DP | (-0.074, 0.062), FALSE | (-0.594, 0.328), FALSE | (0.116, 0.187), TRUE | (-0.509, 0.041), FALSE | (0.14, 0.211), TRUE | (0.816, 2.51), FALSE | (-0.045, 0.099), FALSE | (-0.343, 0.741), FALSE | (-0.049, 0.113), FALSE | (-0.187, 0.293), FALSE | (-0.025, 0.164), FALSE | (-0.147, 0.124), FALSE |
| CD-cML | (-0.054, 0.036), FALSE | (-0.371, 0.188), FALSE | (0.127, 0.177), TRUE | (-0.46, -0.038), TRUE | (0.147, 0.198), TRUE | (1.235, 2.126), FALSE | (-0.017, 0.075), FALSE | (-0.24, 0.684), FALSE | (-0.022, 0.094), FALSE | (-0.153, 0.217), FALSE | (-0.011, 0.145), FALSE | (-0.133, 0.114), FALSE |
| CD-Ratio | (-0.042, 0.039), FALSE | (-0.387, 0.105), FALSE | (0.114, 0.162), TRUE | (-0.099, 0.253), FALSE | (0.139, 0.187), TRUE | (0.212, 0.557), TRUE | (-0.014, 0.054), FALSE | (-0.258, 0.654), FALSE | (-0.026, 0.087), FALSE | (-0.149, 0.21), FALSE | (-0.015, 0.137), FALSE | (-0.133, 0.114), FALSE |
| CD-Egger | (-0.063, 0.062), FALSE | (-0.581, 0.187), FALSE | (0.108, 0.18), TRUE | (-0.358, 2.127), FALSE | (0.133, 0.208), TRUE | (0.006, 1.689), FALSE | (-0.026, 0.088), FALSE | (-0.285, 0.714), FALSE | (-0.052, 0.125), FALSE | (-0.235, 0.262), FALSE | (-0.056, 0.17), FALSE | (-0.13, 0.115), FALSE |
| LHC-MR | (-0.196, 0.008), FALSE | (-0.041, 0.663), FALSE | (0.127, 0.213), TRUE | (0.043, 0.529), TRUE | (0.071, 0.291), TRUE | (-6.321, 6.806), FALSE | (0.029, 0.344), TRUE | (0.06, 0.775), TRUE | (-0.078, 0.191), FALSE | (-0.027, 0.943), FALSE | (-0.138, 0.267), FALSE | (-0.44, 0.161), FALSE |
| Steiger | 0.73, TRUE | 0.143, FALSE | 0.909, TRUE | 0.013, FALSE | 0.907, TRUE | 0.011, FALSE | 0.462, FALSE | 0.038, FALSE | 0.553, TRUE | 0.368, FALSE | 0.633, TRUE | 0.347, FALSE |

S5 Table: Inferring causal effects between first 6 risk factors and T2D. In each cell we show the Bonferroni adjusted 1-0.05/96 $\approx$ 0.9995 confidence intervals (CIs) of $\theta$ for the MR methods, and CIs of $K$ for the CD methods; for Steiger's method, we show the proportion of SNPs giving significant result. TRUE/FALSE in each cell indicates whether the result is significant or not, and the cells giving significant results are marked in red.

| Method \ Direction | TG to T2D | T2D to TG | LDL to T2D | T2D to LDL | HDL to T2D | T2D to HDL | Height to T2D | T2D to Height | BMI to T2D | T2D to BMI | BF to T2D | T2D to BF |
|---|---|---|---|---|---|---|---|---|---|---|---|---|
| MR-cML-DP-S | (-0.382, 0.483), FALSE | (-0.05, 0.15), FALSE | (-0.36, 0.082), FALSE | (-0.021, 0.045), FALSE | (-0.579, 0.165), FALSE | (-0.084, 0.049), FALSE | (-0.237, 0.145), FALSE | (-0.034, 0.057), FALSE | (0.426, 1.302), TRUE | (-0.105, -0.022), TRUE | (-0.17, 3.255), FALSE | (-0.094, 0.021), FALSE |
| MR-cML-S | (-0.172, 0.282), FALSE | (0.005, 0.11), TRUE | (-0.316, 0.029), FALSE | (-0.015, 0.042), FALSE | (-0.411, 0.002), FALSE | (-0.038, 0.018), FALSE | (-0.184, 0.09), FALSE | (-0.019, 0.043), FALSE | (0.484, 1.177), TRUE | (-0.094, -0.042), TRUE | (0.404, 2.836), TRUE | (-0.073, 0.004), FALSE |
| CD-cML-DP-S | (-0.229, 0.275), FALSE | (-0.062, 0.22), FALSE | (-0.217, 0.046), FALSE | (-0.029, 0.068), FALSE | (-0.327, 0.102), FALSE | (-0.118, 0.065), FALSE | (-0.189, 0.11), FALSE | (-0.04, 0.069), FALSE | (0.379, 1.223), FALSE | (-0.114, -0.02), TRUE | (-0.192, 2.647), FALSE | (-0.121, 0.024), FALSE |
| CD-cML-S | (-0.1, 0.152), FALSE | (-0.007, 0.162), FALSE | (-0.195, 0.013), FALSE | (-0.021, 0.064), FALSE | (-0.224, 0.011), FALSE | (-0.063, 0.027), FALSE | (-0.149, 0.071), FALSE | (-0.024, 0.052), FALSE | (0.435, 1.087), FALSE | (-0.098, -0.044), TRUE | (0.319, 2.27), FALSE | (-0.096, 0.001), FALSE |
| CD-Ratio-S | (-0.102, 0.127), FALSE | (0.009, 0.099), TRUE | (-0.167, 0.032), FALSE | (-0.021, 0.063), FALSE | (-0.198, 0.005), FALSE | (-0.076, 0.01), FALSE | (-0.131, 0.072), FALSE | (-0.025, 0.035), FALSE | (0.409, 1.043), FALSE | (-0.078, -0.03), TRUE | (-0.249, 1.168), FALSE | (-0.085, 0.001), FALSE |
| CD-Egger-S | (-0.166, 0.235), FALSE | (-0.041, 0.184), FALSE | (-0.203, 0.061), FALSE | (-0.02, 0.066), FALSE | (-0.258, 0.046), FALSE | (-0.172, 0.053), FALSE | (-0.179, 0.114), FALSE | (-0.025, 0.047), FALSE | (0.385, 1.182), FALSE | (-0.213, 0.148), FALSE | (-1.304, 2.297), FALSE | (-0.189, 0.116), FALSE |
| MR-cML-DP | (-0.382, 0.483), FALSE | (-0.05, 0.15), FALSE | (-0.36, 0.082), FALSE | (-0.021, 0.045), FALSE | (-0.569, 0.165), FALSE | (-0.084, 0.049), FALSE | (-0.237, 0.145), FALSE | (-0.034, 0.057), FALSE | (0.493, 1.358), TRUE | (-0.105, -0.022), TRUE | (0.395, 3.477), TRUE | (-0.094, 0.021), FALSE |
| MR-cML | (-0.172, 0.282), FALSE | (0.005, 0.11), TRUE | (-0.316, 0.029), FALSE | (-0.015, 0.042), FALSE | (-0.411, 0.002), FALSE | (-0.038, 0.018), FALSE | (-0.184, 0.09), FALSE | (-0.019, 0.043), FALSE | (0.586, 1.222), TRUE | (-0.094, -0.042), TRUE | (1.077, 2.983), TRUE | (-0.073, 0.004), FALSE |
| CD-cML-DP | (-0.229, 0.275), FALSE | (-0.062, 0.22), FALSE | (-0.217, 0.046), FALSE | (-0.029, 0.068), FALSE | (-0.326, 0.109), FALSE | (-0.118, 0.065), FALSE | (-0.189, 0.11), FALSE | (-0.04, 0.069), FALSE | (0.447, 1.274), FALSE | (-0.114, -0.02), TRUE | (0.384, 2.679), FALSE | (-0.121, 0.024), FALSE |
| CD-cML | (-0.1, 0.152), FALSE | (-0.007, 0.162), FALSE | (-0.195, 0.013), FALSE | (-0.021, 0.064), FALSE | (-0.224, 0.011), FALSE | (-0.063, 0.027), FALSE | (-0.149, 0.071), FALSE | (-0.024, 0.052), FALSE | (0.534, 1.131), FALSE | (-0.098, -0.044), TRUE | (0.843, 2.328), FALSE | (-0.096, 0.001), FALSE |
| CD-Ratio | (-0.102, 0.127), FALSE | (0.009, 0.099), TRUE | (-0.167, 0.032), FALSE | (-0.021, 0.063), FALSE | (-0.207, -0.005), TRUE | (-0.076, 0.01), FALSE | (-0.131, 0.072), FALSE | (-0.025, 0.035), FALSE | (0.483, 1.066), FALSE | (-0.078, -0.03), TRUE | (0.249, 1.457), FALSE | (-0.085, 0.001), FALSE |
| CD-Egger | (-0.166, 0.235), FALSE | (-0.041, 0.184), FALSE | (-0.203, 0.061), FALSE | (-0.02, 0.066), FALSE | (-0.291, 0.034), FALSE | (-0.172, 0.053), FALSE | (-0.179, 0.114), FALSE | (-0.025, 0.047), FALSE | (-0.061, 1.396), FALSE | (-0.213, 0.148), FALSE | (-0.456, 2.198), FALSE | (-0.189, 0.116), FALSE |
| LHC-MR | (-0.054, 1.011), FALSE | (0.065, 0.295), TRUE | (-0.12, 0.708), FALSE | (-0.185, 0.205), FALSE | (-0.917, -0.131), TRUE | (-0.171, 0.004), FALSE | (-0.609, 0.484), FALSE | (-0.261, 0.049), FALSE | (0.998, 1.002), TRUE | (-0.228, -0.001), TRUE | (0.92, 1.079), TRUE | (-1.112, 0.935), FALSE |
| Steiger | 0.266, FALSE | 0.172, FALSE | 0.321, FALSE | 0.111, FALSE | 0.348, FALSE | 0.13, FALSE | 0.061, FALSE | 0.034, FALSE | 0, FALSE | 0.171, FALSE | 0, FALSE | 0.6, TRUE |

S6 Table: Inferring causal effects between second 6 risk factors and T2D. In each cell we show the Bonferroni adjusted 1-0.05/96 $\approx$ 0.9995 confidence intervals (CIs) of $\theta$ for the MR methods, and CIs of $K$ for the CD methods; for Steiger's method, we show the proportion of SNPs giving significant result. TRUE/FALSE in each cell indicates whether the result is significant or not, and the cells giving significant results are marked in red.

| Method \ Direction | BW to T2D | T2D to BW | DBP to T2D | T2D to DBP | SBP to T2D | T2D to SBP | FG to T2D | T2D to FG | Smoke to T2D | T2D to Smoke | Alcohol to T2D | T2D to Alcohol |
|---|---|---|---|---|---|---|---|---|---|---|---|---|
| MR-cML-DP-S | (-0.98, 0.226), FALSE | (-0.022, 0.054), FALSE | (-0.009, 0.056), FALSE | (-0.703, 0.412), FALSE | (0.005, 0.039), TRUE | (-0.531, 1.585), FALSE | (0.927, 3.134), TRUE | (0.032, 0.117), TRUE | (-0.568, 0.614), FALSE | (-0.062, 0.029), FALSE | (-0.852, 1.662), FALSE | (-0.045, 0.011), FALSE |
| MR-cML-S | (-0.831, 0.01), FALSE | (-0.015, 0.044), FALSE | (0, 0.044), TRUE | (-0.522, 0.041), FALSE | (0.009, 0.034), TRUE | (0.32, 1.067), TRUE | (1.167, 2.882), TRUE | (0.042, 0.106), TRUE | (-0.315, 0.244), FALSE | (-0.058, 0.023), FALSE | (-0.564, 1.372), FALSE | (-0.029, 0.001), FALSE |
| CD-cML-DP-S | (-0.749, 0.19), FALSE | (-0.028, 0.072), FALSE | (-0.06, 0.447), FALSE | (-0.09, 0.054), FALSE | (0.058, 0.539), TRUE | (-0.03, 0.109), FALSE | (0.411, 1.336), FALSE | (0.079, 0.261), TRUE | (-0.781, 0.842), FALSE | (-0.039, 0.02), FALSE | (-0.66, 1.192), FALSE | (-0.061, 0.017), FALSE |
| CD-cML-S | (-0.622, 0.012), FALSE | (-0.019, 0.059), FALSE | (0.006, 0.356), TRUE | (-0.065, 0.005), FALSE | (0.116, 0.466), TRUE | (0.024, 0.073), TRUE | (0.514, 1.218), FALSE | (0.099, 0.238), TRUE | (-0.481, 0.402), FALSE | (-0.036, 0.016), FALSE | (-0.438, 0.959), FALSE | (-0.037, 0.002), FALSE |
| CD-Ratio-S | (-0.562, 0.018), FALSE | (-0.044, 0.028), FALSE | (0.018, 0.352), TRUE | (-0.01, 0.024), FALSE | (0.13, 0.47), TRUE | (0.024, 0.062), TRUE | (0.215, 0.671), TRUE | (0.107, 0.235), TRUE | (-0.44, 0.383), FALSE | (-0.036, 0.015), FALSE | (-0.455, 0.831), FALSE | (-0.037, -0.001), TRUE |
| CD-Egger-S | (-0.724, 0.201), FALSE | (-0.177, 0.123), FALSE | (-0.035, 0.433), FALSE | (-0.038, 0.057), FALSE | (0.103, 0.539), TRUE | (0, 0.105), FALSE | (-0.055, 1.224), FALSE | (0.086, 0.276), TRUE | (-0.516, 0.501), FALSE | (-0.035, 0.016), FALSE | (-0.527, 1.036), FALSE | (-0.049, 0.006), FALSE |
| MR-cML-DP | (-1.031, 0.245), FALSE | (-0.022, 0.054), FALSE | (-0.008, 0.056), FALSE | (-0.703, 0.412), FALSE | (0.004, 0.039), TRUE | (-0.531, 1.585), FALSE | (0.847, 3.322), TRUE | (0.037, 0.11), TRUE | (-0.568, 0.614), FALSE | (-0.062, 0.029), FALSE | (-0.852, 1.662), FALSE | (-0.045, 0.011), FALSE |
| MR-cML | (-0.831, 0.01), FALSE | (-0.015, 0.044), FALSE | (0, 0.044), TRUE | (-0.522, 0.041), FALSE | (0.009, 0.034), TRUE | (0.32, 1.067), TRUE | (1.175, 2.874), TRUE | (0.042, 0.106), TRUE | (-0.315, 0.244), FALSE | (-0.058, 0.023), FALSE | (-0.564, 1.372), FALSE | (-0.029, 0.001), FALSE |
| CD-cML-DP | (-0.798, 0.21), FALSE | (-0.028, 0.072), FALSE | (-0.061, 0.452), FALSE | (-0.09, 0.054), FALSE | (0.061, 0.534), TRUE | (-0.03, 0.109), FALSE | (0.411, 1.427), FALSE | (0.088, 0.251), TRUE | (-0.781, 0.842), FALSE | (-0.039, 0.02), FALSE | (-0.66, 1.192), FALSE | (-0.061, 0.017), FALSE |
| CD-cML | (-0.622, 0.012), FALSE | (-0.019, 0.059), FALSE | (0.006, 0.356), TRUE | (-0.065, 0.005), FALSE | (0.116, 0.466), TRUE | (0.024, 0.073), TRUE | (0.603, 1.324), FALSE | (0.099, 0.238), TRUE | (-0.481, 0.402), FALSE | (-0.036, 0.016), FALSE | (-0.438, 0.959), FALSE | (-0.037, 0.002), FALSE |
| CD-Ratio | (-0.697, -0.13), TRUE | (-0.044, 0.028), FALSE | (0.022, 0.356), TRUE | (-0.01, 0.024), FALSE | (0.148, 0.487), TRUE | (0.024, 0.062), TRUE | (0.268, 0.719), TRUE | (0.115, 0.243), TRUE | (-0.44, 0.383), FALSE | (-0.036, 0.015), FALSE | (-0.455, 0.831), FALSE | (-0.037, -0.001), TRUE |
| CD-Egger | (-1.104, 0.172), FALSE | (-0.177, 0.123), FALSE | (-0.034, 0.462), FALSE | (-0.038, 0.057), FALSE | (0.09, 0.633), TRUE | (0, 0.105), FALSE | (-0.505, 1.715), FALSE | (-0.033, 0.442), FALSE | (-0.516, 0.501), FALSE | (-0.035, 0.016), FALSE | (-0.527, 1.036), FALSE | (-0.049, 0.006), FALSE |
| LHC-MR | (-1.622, 1.118), FALSE | (-0.49, 0.068), FALSE | (-0.11, 0.789), FALSE | (-0.021, 0.248), FALSE | (-0.016, 1.007), FALSE | (-0.081, 0.216), FALSE | (-0.227, 0.985), FALSE | (0.239, 0.5), TRUE | (-0.683, 1.675), FALSE | (-0.048, 0.118), FALSE | (-0.291, 1.227), FALSE | (-0.12, 0.029), FALSE |
| Steiger | 0.038, FALSE | 0.245, FALSE | 0.005, FALSE | 0.067, FALSE | 0.005, FALSE | 0.048, FALSE | 0.091, FALSE | 0.5, TRUE | 0.032, FALSE | 0.355, FALSE | 0, FALSE | 0.359, FALSE |

S7 Table: Inferring causal effects between first 6 risk factors and Asthma. In each cell we show the Bonferroni adjusted 1-0.05/96 $\approx$ 0.9995 confidence intervals (CIs) of $\theta$ for the MR methods, and CIs of $K$ for the CD methods; for Steiger's method, we show the proportion of SNPs giving significant result. TRUE/FALSE in each cell indicates whether the result is significant or not, and the cells giving significant results are marked in red.

| Method \ Direction | TG to Asthma | Asthma to TG | LDL to Asthma | Asthma to LDL | HDL to Asthma | Asthma to HDL | Height to Asthma | Asthma to Height | BMI to Asthma | Asthma to BMI | BF to Asthma | Asthma to BF |
|---|---|---|---|---|---|---|---|---|---|---|---|---|
| MR-cML-DP-S | (-0.305, 0.185), FALSE | (-0.056, 0.077), FALSE | (-0.152, 0.143), FALSE | (-0.064, 0.035), FALSE | (-0.204, 0.164), FALSE | (-0.036, 0.059), FALSE | (-0.088, 0.131), FALSE | (-0.038, 0.023), FALSE | (-0.14, 0.41), FALSE | (-0.034, 0.028), FALSE | (-0.392, 0.645), FALSE | (-0.03, 0.061), FALSE |
| MR-cML-S | (-0.222, 0.094), FALSE | (-0.037, 0.055), FALSE | (-0.13, 0.106), FALSE | (-0.055, 0.031), FALSE | (-0.154, 0.114), FALSE | (-0.033, 0.055), FALSE | (-0.058, 0.122), FALSE | (-0.04, 0.023), FALSE | (-0.092, 0.348), FALSE | (-0.033, 0.026), FALSE | (-0.38, 0.618), FALSE | (-0.028, 0.058), FALSE |
| CD-cML-DP-S | (-0.068, 0.041), FALSE | (-0.173, 0.24), FALSE | (-0.037, 0.036), FALSE | (-0.196, 0.113), FALSE | (-0.049, 0.039), FALSE | (-0.112, 0.183), FALSE | (-0.029, 0.043), FALSE | (-0.116, 0.073), FALSE | (-0.049, 0.153), FALSE | (-0.078, 0.065), FALSE | (-0.113, 0.19), FALSE | (-0.091, 0.177), FALSE |
| CD-cML-S | (-0.05, 0.021), FALSE | (-0.117, 0.174), FALSE | (-0.032, 0.027), FALSE | (-0.165, 0.102), FALSE | (-0.036, 0.026), FALSE | (-0.1, 0.17), FALSE | (-0.019, 0.04), FALSE | (-0.121, 0.072), FALSE | (-0.032, 0.131), FALSE | (-0.076, 0.06), FALSE | (-0.108, 0.182), FALSE | (-0.084, 0.17), FALSE |
| CD-Ratio-S | (-0.053, 0.012), FALSE | (-0.113, 0.146), FALSE | (-0.025, 0.032), FALSE | (-0.186, 0.053), FALSE | (-0.029, 0.03), FALSE | (-0.181, 0.068), FALSE | (-0.026, 0.031), FALSE | (-0.118, 0.072), FALSE | (-0.033, 0.129), FALSE | (-0.077, 0.06), FALSE | (-0.11, 0.179), FALSE | (-0.084, 0.169), FALSE |
| CD-Egger-S | (-0.07, 0.024), FALSE | (-0.177, 0.201), FALSE | (-0.04, 0.042), FALSE | (-0.231, 0.081), FALSE | (-0.04, 0.043), FALSE | (-0.334, 0.182), FALSE | (-0.035, 0.043), FALSE | (-0.114, 0.111), FALSE | (-0.04, 0.137), FALSE | (-0.076, 0.062), FALSE | (-0.13, 0.167), FALSE | (-0.085, 0.169), FALSE |
| MR-cML-DP | (-0.305, 0.185), FALSE | (-0.056, 0.077), FALSE | (-0.152, 0.143), FALSE | (-0.064, 0.035), FALSE | (-0.189, 0.15), FALSE | (-0.036, 0.059), FALSE | (-0.088, 0.131), FALSE | (-0.043, 0.023), FALSE | (-0.14, 0.41), FALSE | (-0.034, 0.028), FALSE | (-0.392, 0.645), FALSE | (-0.03, 0.061), FALSE |
| MR-cML | (-0.222, 0.094), FALSE | (-0.037, 0.055), FALSE | (-0.13, 0.106), FALSE | (-0.055, 0.031), FALSE | (-0.154, 0.114), FALSE | (-0.033, 0.055), FALSE | (-0.058, 0.122), FALSE | (-0.04, 0.023), FALSE | (-0.092, 0.348), FALSE | (-0.033, 0.026), FALSE | (-0.38, 0.618), FALSE | (-0.028, 0.058), FALSE |
| CD-cML-DP | (-0.068, 0.041), FALSE | (-0.173, 0.24), FALSE | (-0.037, 0.036), FALSE | (-0.196, 0.113), FALSE | (-0.045, 0.036), FALSE | (-0.112, 0.183), FALSE | (-0.029, 0.043), FALSE | (-0.132, 0.073), FALSE | (-0.049, 0.153), FALSE | (-0.078, 0.065), FALSE | (-0.113, 0.19), FALSE | (-0.091, 0.177), FALSE |
| CD-cML | (-0.05, 0.021), FALSE | (-0.117, 0.174), FALSE | (-0.032, 0.027), FALSE | (-0.165, 0.102), FALSE | (-0.036, 0.026), FALSE | (-0.1, 0.17), FALSE | (-0.019, 0.04), FALSE | (-0.121, 0.072), FALSE | (-0.032, 0.131), FALSE | (-0.076, 0.06), FALSE | (-0.108, 0.182), FALSE | (-0.084, 0.17), FALSE |
| CD-Ratio | (-0.053, 0.012), FALSE | (-0.113, 0.146), FALSE | (-0.025, 0.032), FALSE | (-0.186, 0.053), FALSE | (-0.031, 0.029), FALSE | (-0.181, 0.068), FALSE | (-0.026, 0.031), FALSE | (-0.132, 0.056), FALSE | (-0.033, 0.129), FALSE | (-0.077, 0.06), FALSE | (-0.11, 0.179), FALSE | (-0.084, 0.169), FALSE |
| CD-Egger | (-0.07, 0.024), FALSE | (-0.177, 0.201), FALSE | (-0.04, 0.042), FALSE | (-0.231, 0.081), FALSE | (-0.068, 0.053), FALSE | (-0.334, 0.182), FALSE | (-0.035, 0.043), FALSE | (-0.458, 0.311), FALSE | (-0.04, 0.137), FALSE | (-0.076, 0.062), FALSE | (-0.13, 0.167), FALSE | (-0.085, 0.169), FALSE |
| LHC-MR | (-0.298, 0.059), FALSE | (-0.663, 0.317), FALSE | (-0.18, 0.061), FALSE | (-0.175, 0.312), FALSE | (-0.042, 0.278), FALSE | (-0.836, 0.137), FALSE | (-0.152, 0.107), FALSE | (-0.792, 0.09), FALSE | (-0.087, 0.167), FALSE | (-0.076, 0.133), FALSE | (-0.278, 0.073), FALSE | (-0.524, 0.4), FALSE |
| Steiger | 0.8, TRUE | 0.2, FALSE | 0.807, TRUE | 0.182, FALSE | 0.845, TRUE | 0.155, FALSE | 0.903, TRUE | 0.024, FALSE | 0.714, TRUE | 0.208, FALSE | 0.346, FALSE | 0.654, TRUE |

S8 Table: Inferring causal effects between second 6 risk factors and Asthma. In each cell we show the Bonferroni adjusted 1-0.05/96 $\approx$ 0.9995 confidence intervals (CIs) of $\theta$ for the MR methods, and CIs of $K$ for the CD methods; for Steiger's method, we show the proportion of SNPs giving significant result. TRUE/FALSE in each cell indicates whether the result is significant or not, and the cells giving significant results are marked in red.

| Method \ Direction | BW to Asthma | Asthma to BW | DBP to Asthma | Asthma to DBP | SBP to Asthma | Asthma to SBP | FG to Asthma | Asthma to FG | Smoke to Asthma | Asthma to Smoke | Alcohol to Asthma | Asthma to Alcohol |
|---|---|---|---|---|---|---|---|---|---|---|---|---|
| MR-cML-DP-S | (-0.233, 0.479), FALSE | (-0.043, 0.023), FALSE | (-0.023, 0.018), FALSE | (-0.443, 0.222), FALSE | (-0.01, 0.012), FALSE | (-0.646, 0.331), FALSE | (-0.722, 0.371), FALSE | (-0.022, 0.05), FALSE | (-0.16, 0.201), FALSE | (-0.052, 0.044), FALSE | (-0.864, 0.609), FALSE | (-0.027, 0.017), FALSE |
| MR-cML-S | (-0.14, 0.393), FALSE | (-0.041, 0.021), FALSE | (-0.018, 0.012), FALSE | (-0.355, 0.069), FALSE | (-0.008, 0.009), FALSE | (-0.536, 0.159), FALSE | (-0.558, 0.184), FALSE | (-0.016, 0.045), FALSE | (-0.167, 0.222), FALSE | (-0.047, 0.041), FALSE | (-0.75, 0.525), FALSE | (-0.022, 0.011), FALSE |
| CD-cML-DP-S | (-0.068, 0.147), FALSE | (-0.141, 0.076), FALSE | (-0.075, 0.057), FALSE | (-0.132, 0.067), FALSE | (-0.058, 0.066), FALSE | (-0.116, 0.059), FALSE | (-0.126, 0.065), FALSE | (-0.128, 0.289), FALSE | (-0.097, 0.123), FALSE | (-0.084, 0.073), FALSE | (-0.257, 0.192), FALSE | (-0.088, 0.056), FALSE |
| CD-cML-S | (-0.04, 0.121), FALSE | (-0.133, 0.066), FALSE | (-0.057, 0.039), FALSE | (-0.106, 0.022), FALSE | (-0.046, 0.052), FALSE | (-0.095, 0.028), FALSE | (-0.098, 0.032), FALSE | (-0.096, 0.261), FALSE | (-0.102, 0.136), FALSE | (-0.077, 0.067), FALSE | (-0.223, 0.168), FALSE | (-0.07, 0.036), FALSE |
| CD-Ratio-S | (-0.048, 0.11), FALSE | (-0.131, 0.067), FALSE | (-0.056, 0.038), FALSE | (-0.079, 0.03), FALSE | (-0.044, 0.051), FALSE | (-0.053, 0.053), FALSE | (-0.084, 0.042), FALSE | (-0.1, 0.24), FALSE | (-0.101, 0.136), FALSE | (-0.076, 0.067), FALSE | (-0.223, 0.167), FALSE | (-0.062, 0.038), FALSE |
| CD-Egger-S | (-0.077, 0.134), FALSE | (-0.131, 0.065), FALSE | (-0.068, 0.052), FALSE | (-0.126, 0.065), FALSE | (-0.053, 0.065), FALSE | (-0.096, 0.11), FALSE | (-0.174, 0.098), FALSE | (-0.142, 0.265), FALSE | (-0.112, 0.131), FALSE | (-0.077, 0.068), FALSE | (-0.26, 0.197), FALSE | (-0.076, 0.06), FALSE |
| MR-cML-DP | (-0.233, 0.479), FALSE | (-0.043, 0.023), FALSE | (-0.023, 0.018), FALSE | (-0.443, 0.222), FALSE | (-0.01, 0.012), FALSE | (-0.646, 0.331), FALSE | (-0.722, 0.371), FALSE | (-0.022, 0.05), FALSE | (-0.16, 0.201), FALSE | (-0.052, 0.044), FALSE | (-0.864, 0.609), FALSE | (-0.027, 0.017), FALSE |
| MR-cML | (-0.14, 0.393), FALSE | (-0.041, 0.021), FALSE | (-0.018, 0.012), FALSE | (-0.355, 0.069), FALSE | (-0.008, 0.009), FALSE | (-0.536, 0.159), FALSE | (-0.558, 0.184), FALSE | (-0.016, 0.045), FALSE | (-0.167, 0.222), FALSE | (-0.047, 0.041), FALSE | (-0.75, 0.525), FALSE | (-0.022, 0.011), FALSE |
| CD-cML-DP | (-0.068, 0.147), FALSE | (-0.141, 0.076), FALSE | (-0.075, 0.057), FALSE | (-0.132, 0.067), FALSE | (-0.058, 0.066), FALSE | (-0.116, 0.059), FALSE | (-0.126, 0.065), FALSE | (-0.128, 0.289), FALSE | (-0.097, 0.123), FALSE | (-0.084, 0.073), FALSE | (-0.257, 0.192), FALSE | (-0.088, 0.056), FALSE |
| CD-cML | (-0.04, 0.121), FALSE | (-0.133, 0.066), FALSE | (-0.057, 0.039), FALSE | (-0.106, 0.022), FALSE | (-0.046, 0.052), FALSE | (-0.095, 0.028), FALSE | (-0.098, 0.032), FALSE | (-0.096, 0.261), FALSE | (-0.102, 0.136), FALSE | (-0.077, 0.067), FALSE | (-0.223, 0.168), FALSE | (-0.07, 0.036), FALSE |
| CD-Ratio | (-0.048, 0.11), FALSE | (-0.131, 0.067), FALSE | (-0.056, 0.038), FALSE | (-0.079, 0.03), FALSE | (-0.044, 0.051), FALSE | (-0.053, 0.053), FALSE | (-0.084, 0.042), FALSE | (-0.1, 0.24), FALSE | (-0.101, 0.136), FALSE | (-0.076, 0.067), FALSE | (-0.223, 0.167), FALSE | (-0.062, 0.038), FALSE |
| CD-Egger | (-0.077, 0.134), FALSE | (-0.131, 0.065), FALSE | (-0.068, 0.052), FALSE | (-0.126, 0.065), FALSE | (-0.053, 0.065), FALSE | (-0.096, 0.11), FALSE | (-0.174, 0.098), FALSE | (-0.142, 0.265), FALSE | (-0.112, 0.131), FALSE | (-0.077, 0.068), FALSE | (-0.26, 0.197), FALSE | (-0.076, 0.06), FALSE |
| LHC-MR | (-0.292, 0.175), FALSE | (-0.494, 0.211), FALSE | (-0.128, 0.148), FALSE | (-0.144, 0.388), FALSE | (-0.093, 0.174), FALSE | (-0.042, 0.223), FALSE | (-2.037, 2.356), FALSE | (-1.804, 1.328), FALSE | (-0.197, 0.139), FALSE | (-0.136, 0.172), FALSE | (-0.279, 0.079), FALSE | (-0.162, 0.063), FALSE |
| Steiger | 0.712, TRUE | 0.271, FALSE | 0.5, TRUE | 0.024, FALSE | 0.513, TRUE | 0.031, FALSE | 0.414, FALSE | 0.517, TRUE | 0.471, FALSE | 0.5, TRUE | 0.357, FALSE | 0.405, TRUE |

## S2.2 Pairs of 4 Diseases

S41 Fig: Causal relationship between pairs of 4 diseases.

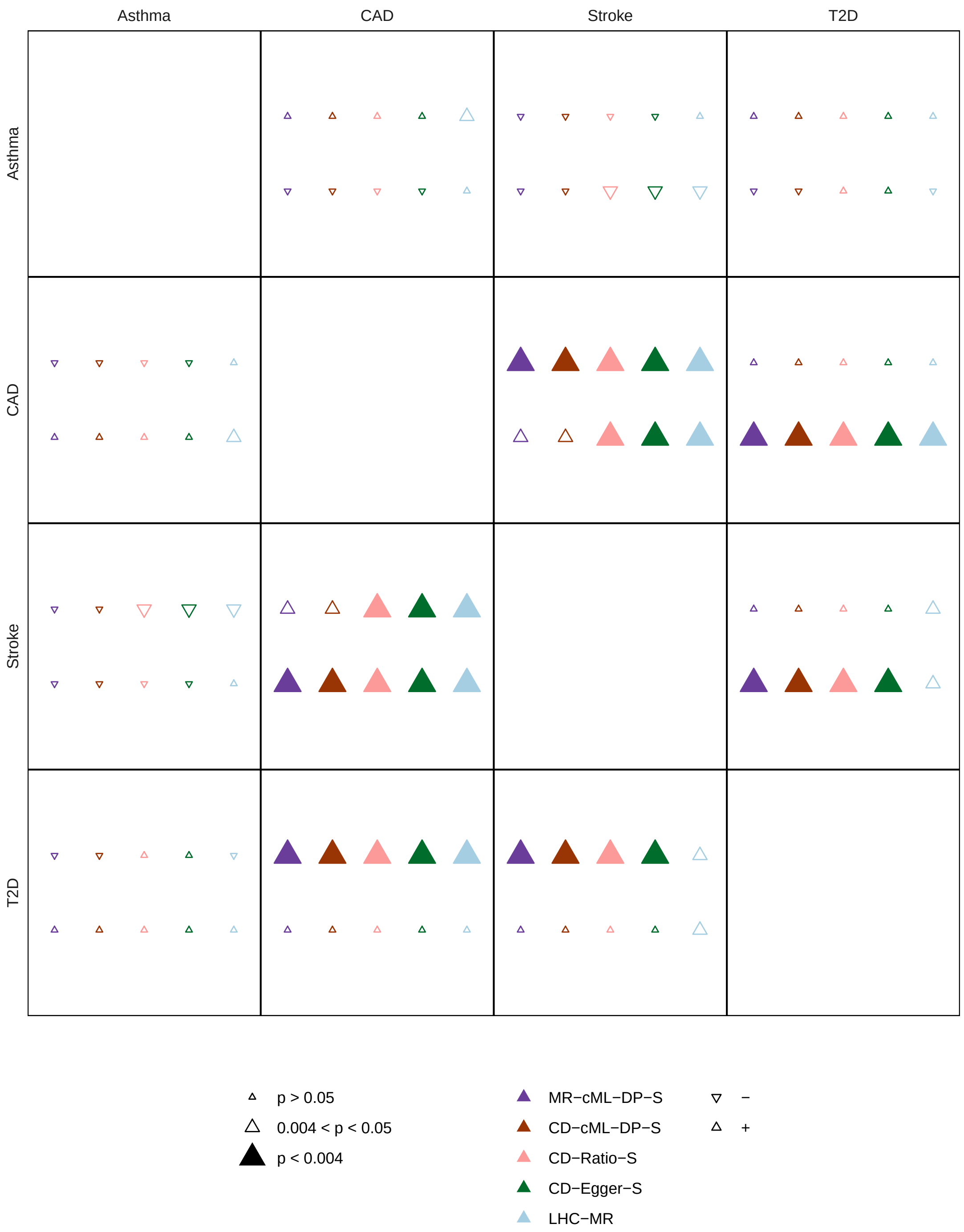

S9 Table: Inferring causal effects between pairs of 4 diseases. In each cell we show the Bonferroni adjusted 1-0.05/12 $\approx$ 0.996 confidence intervals (CIs) of $\theta$ for the MR methods, and CIs of $K$ for the CD methods; for Steiger's method, we show the proportion of SNPs giving significant result. TRUE/FALSE in each cell indicates whether the result is significant or not, and the cells giving significant results are marked in red.

| Method \ Direction | CAD to Stroke | Stroke to CAD | CAD to T2D | T2D to CAD | CAD to Asthma | Asthma to CAD | Stroke to T2D | T2D to Stroke | Stroke to Asthma | Asthma to Stroke | T2D to Asthma | Asthma to T2D |
|---|---|---|---|---|---|---|---|---|---|---|---|---|
| MR-cML-DP-S | (0.102, 0.318), TRUE | (-0.032, 0.414), FALSE | (-0.221, 0.233), FALSE | (0.009, 0.134), TRUE | (-0.179, 0.17), FALSE | (-0.043, 0.099), FALSE | (-0.313, 0.523), FALSE | (0.011, 0.144), TRUE | (-0.442, 0.132), FALSE | (-0.077, 0.044), FALSE | (-0.126, 0.095), FALSE | (-0.209, 0.233), FALSE |
| MR-cML-S | (0.135, 0.25), TRUE | (0.05, 0.309), TRUE | (-0.094, 0.165), FALSE | (0.019, 0.111), TRUE | (-0.125, 0.13), FALSE | (-0.019, 0.084), FALSE | (-0.223, 0.473), FALSE | (0.023, 0.124), TRUE | (-0.397, 0.068), FALSE | (-0.069, 0.04), FALSE | (-0.096, 0.072), FALSE | (-0.117, 0.145), FALSE |
| CD-cML-DP-S | (0.099, 0.29), TRUE | (-0.022, 0.432), FALSE | (-0.592, 0.623), FALSE | (0.003, 0.048), TRUE | (-0.196, 0.175), FALSE | (-0.039, 0.087), FALSE | (-0.878, 1.387), FALSE | (0.003, 0.048), TRUE | (-0.511, 0.174), FALSE | (-0.065, 0.035), FALSE | (-0.049, 0.04), FALSE | (-0.496, 0.565), FALSE |
| CD-cML-S | (0.131, 0.232), TRUE | (0.057, 0.323), TRUE | (-0.278, 0.449), FALSE | (0.007, 0.039), TRUE | (-0.139, 0.095), FALSE | (-0.017, 0.074), FALSE | (-0.674, 1.276), FALSE | (0.007, 0.041), TRUE | (-0.449, 0.097), FALSE | (-0.058, 0.032), FALSE | (-0.037, 0.03), FALSE | (-0.281, 0.347), FALSE |
| CD-Ratio-S | (0.124, 0.219), TRUE | (0.088, 0.322), TRUE | (-0.278, 0.385), FALSE | (0.009, 0.037), TRUE | (-0.141, 0.06), FALSE | (-0.025, 0.058), FALSE | (-0.44, 1.175), FALSE | (0.006, 0.039), TRUE | (-0.401, 0.043), FALSE | (-0.058, 0.032), FALSE | (-0.03, 0.03), FALSE | (-0.277, 0.305), FALSE |
| CD-Egger-S | (0.108, 0.271), TRUE | (0.002, 0.525), TRUE | (-0.47, 0.636), FALSE | (0.005, 0.044), TRUE | (-0.176, 0.111), FALSE | (-0.071, 0.142), FALSE | (-0.784, 1.365), FALSE | (0.006, 0.042), TRUE | (-0.535, 0.078), FALSE | (-0.058, 0.034), FALSE | (-0.043, 0.046), FALSE | (-0.355, 0.382), FALSE |
| MR-cML-DP | (0.098, 0.327), TRUE | (-0.021, 0.417), FALSE | (-0.221, 0.233), FALSE | (0.009, 0.134), TRUE | (-0.178, 0.165), FALSE | (-0.043, 0.099), FALSE | (-0.313, 0.523), FALSE | (0.011, 0.144), TRUE | (-0.442, 0.132), FALSE | (-0.077, 0.044), FALSE | (-0.126, 0.095), FALSE | (-0.209, 0.233), FALSE |
| MR-cML | (0.135, 0.25), TRUE | (0.05, 0.309), TRUE | (-0.094, 0.165), FALSE | (0.019, 0.111), TRUE | (-0.125, 0.13), FALSE | (-0.019, 0.084), FALSE | (-0.223, 0.473), FALSE | (0.023, 0.124), TRUE | (-0.397, 0.068), FALSE | (-0.069, 0.04), FALSE | (-0.096, 0.072), FALSE | (-0.117, 0.145), FALSE |
| CD-cML-DP | (0.093, 0.303), TRUE | (-0.028, 0.456), FALSE | (-0.592, 0.623), FALSE | (0.003, 0.048), TRUE | (-0.199, 0.173), FALSE | (-0.039, 0.087), FALSE | (-0.878, 1.387), FALSE | (0.003, 0.048), TRUE | (-0.511, 0.174), FALSE | (-0.065, 0.035), FALSE | (-0.049, 0.04), FALSE | (-0.496, 0.565), FALSE |
| CD-cML | (0.131, 0.232), TRUE | (0.057, 0.323), TRUE | (-0.278, 0.449), FALSE | (0.007, 0.039), TRUE | (-0.139, 0.095), FALSE | (-0.017, 0.074), FALSE | (-0.674, 1.276), FALSE | (0.007, 0.041), TRUE | (-0.449, 0.097), FALSE | (-0.058, 0.032), FALSE | (-0.037, 0.03), FALSE | (-0.281, 0.347), FALSE |
| CD-Ratio | (0.131, 0.225), TRUE | (0.123, 0.353), TRUE | (-0.278, 0.385), FALSE | (0.009, 0.037), TRUE | (-0.139, 0.061), FALSE | (-0.025, 0.058), FALSE | (-0.44, 1.175), FALSE | (0.006, 0.039), TRUE | (-0.401, 0.043), FALSE | (-0.058, 0.032), FALSE | (-0.03, 0.03), FALSE | (-0.277, 0.305), FALSE |
| CD-Egger | (0.115, 0.293), TRUE | (0.001, 0.61), TRUE | (-0.47, 0.636), FALSE | (0.005, 0.044), TRUE | (-0.257, 0.128), FALSE | (-0.071, 0.142), FALSE | (-0.784, 1.365), FALSE | (0.006, 0.042), TRUE | (-0.535, 0.078), FALSE | (-0.058, 0.034), FALSE | (-0.043, 0.046), FALSE | (-0.355, 0.382), FALSE |
| LHC-MR | (0.135, 0.386), TRUE | (0.372, 0.759), TRUE | (-0.332, 1.551), FALSE | (0.013, 0.106), TRUE | (-0.138, 0.243), FALSE | (-0.043, 0.23), FALSE | (-0.095, 1.009), FALSE | (-0.032, 0.197), FALSE | (-1.182, 0.442), FALSE | (-0.298, 0.419), FALSE | (-0.201, 0.059), FALSE | (-0.286, 1.613), FALSE |
| Steiger | 0.768, TRUE | 0.146, FALSE | 0.014, FALSE | 0.25, FALSE | 0.411, TRUE | 0.233, FALSE | 0, FALSE | 0.5, FALSE | 0.312, FALSE | 0.531, TRUE | 0.414, FALSE | 0, FALSE |

## S2.3 Links to GWAS Summary Datasets

We downloaded the GWAS summary datasets from the IEU GWAS database [1], which are the same as the data included in R package `TwoSampleMR`. The links are shown in S10 Table.

S10 Table: Links for downloading GWAS summary datasets to be used in real data analysis.

| Trait | Link |
| --- | --- |
| TG | https://gwas.mrcieu.ac.uk/files/ebi-a-GCST002216/ebi-a-GCST002216.vcf.gz |
| LDL | https://gwas.mrcieu.ac.uk/files/ebi-a-GCST002222/ebi-a-GCST002222.vcf.gz |
| HDL | https://gwas.mrcieu.ac.uk/files/ebi-a-GCST002223/ebi-a-GCST002223.vcf.gz |
| Height | https://gwas.mrcieu.ac.uk/files/ieu-a-89/ieu-a-89.vcf.gz |
| BMI | https://gwas.mrcieu.ac.uk/files/ieu-a-835/ieu-a-835.vcf.gz |
| BF | https://gwas.mrcieu.ac.uk/files/ieu-a-999/ieu-a-999.vcf.gz |
| BW | https://gwas.mrcieu.ac.uk/files/ieu-a-1083/ieu-a-1083.vcf.gz |
| DBP | https://gwas.mrcieu.ac.uk/files/ieu-b-39/ieu-b-39.vcf.gz |
| SBP | https://gwas.mrcieu.ac.uk/files/ieu-b-38/ieu-b-38.vcf.gz |
| FG | https://gwas.mrcieu.ac.uk/files/ebi-a-GCST000568/ebi-a-GCST000568.vcf.gz |
| Smoke | https://gwas.mrcieu.ac.uk/files/ieu-b-25/ieu-b-25.vcf.gz |
| Alcohol | https://gwas.mrcieu.ac.uk/files/ieu-b-73/ieu-b-73.vcf.gz |
| CAD | https://gwas.mrcieu.ac.uk/files/ebi-a-GCST005195/ebi-a-GCST005195.vcf.gz |
| Stroke | https://gwas.mrcieu.ac.uk/files/ebi-a-GCST005838/ebi-a-GCST005838.vcf.gz |
| T2D | https://gwas.mrcieu.ac.uk/files/ieu-a-26/ieu-a-26.vcf.gz |
| Asthma | https://gwas.mrcieu.ac.uk/files/ebi-a-GCST006862/ebi-a-GCST006862.vcf.gz |

# S3 Theoretical Results

## S3.1 Proof of Theorem 1

**Theorem 1.** *Under Assumptions 1 and 2, if* $m_{XY}^0 \in \mathscr{M}$*, we have* $P(\hat{m}_I = m_{XY}^0) \to 1$ *and* $P(\hat{B}_{XY}(\hat{m}_I) = B_{XY}^0) \to 1$ *as* $N_1, N_2 \to \infty$*. Furthermore, the cMLE* $\hat{K}_{XY} := \hat{K}_{XY}(\hat{m}_I)$ *is consistent and asymptotically normal:*

$$\sqrt{V}(\hat{K}_{XY} - K_{XY}) \xrightarrow{d} N(0,1), \text{ as } N_1, N_2 \to \infty,$$

*where*

$$V = \sum_{g \in (B_{XY}^0)^c} \frac{\rho_{Xg}^2}{\sigma_{Xg}^2 \cdot K_{XY}^2 + \sigma_{Yg}^2}.$$

*Proof.* First, we show $P\left(\hat{B}_{XY}(m_{XY}^0) = B_{XY}^0\right) \to 1$, which is equivalent to show for any $B_1 \subseteq \{1, \cdots, m\}$ such that $|B_1| = m_{XY}^0$ and $B_1 \neq B_{XY}^0$, $P\left(\hat{B}_{XY}(m_{XY}^0) = B_1\right) \to 0$ as $N_1, N_2 \to \infty$. We have

$$
\begin{aligned}
& P(\hat{B}_{XY}(m_{XY}^0) = B_1) \\
\leq\ & P(\min_{\tilde{K}, \tilde{\rho}_{Xg}} \sum_{g \in B_1^c} \left( \frac{(r_{Xg} - \tilde{\rho}_{Xg})^2}{\mathrm{SE}(r_{Xg})^2} + \frac{(r_{Yg} - \tilde{K}\tilde{\rho}_{Xg})^2}{\mathrm{SE}(r_{Yg})^2} \right) \leq \min_{\tilde{K}, \tilde{\rho}_{Xg}} \sum_{g \in (B_{XY}^0)^c} \left( \frac{(r_{Xg} - \tilde{\rho}_{Xg})^2}{\mathrm{SE}(r_{Xg})^2} + \frac{(r_{Yg} - \tilde{K}\tilde{\rho}_{Xg})^2}{\mathrm{SE}(r_{Yg})^2} \right)) \\
\leq\ & P(\min_{\tilde{K}, \tilde{\rho}_{Xg}} \sum_{g \in B_1^c} \left( \frac{(r_{Xg} - \tilde{\rho}_{Xg})^2}{\mathrm{SE}(r_{Xg})^2} + \frac{(r_{Yg} - \tilde{K}\tilde{\rho}_{Xg})^2}{\mathrm{SE}(r_{Yg})^2} \right) \leq \sum_{g \in (B_{XY}^0)^c} \left( \frac{(r_{Xg} - \rho_{Xg})^2}{\mathrm{SE}(r_{Xg})^2} + \frac{(r_{Yg} - K_{XY}\rho_{Xg})^2}{\mathrm{SE}(r_{Yg})^2} \right)).
\end{aligned}
$$

Note that, for $g \in (B^0_{XY})^c$, $\frac{r_{Xg}-\rho_{Xg}}{\mathrm{SE}(r_{Xg})} \sim N(0,1)$ and $\frac{r_{Yg}-K_{XY}\rho_{Xg}}{\mathrm{SE}(r_{Yg})} \sim N(0,1)$. So for any $\varepsilon > 0$, there exists $C > 0$ such that

$$P\Big(\sum_{g\in(B^0_{XY})^c}\left(\frac{(r_{Xg}-\rho_{Xg})^2}{\mathrm{SE}(r_{Xg})^2}+\frac{(r_{Yg}-K_{XY}\rho_{Xg})^2}{\mathrm{SE}(r_{Yg})^2}\right) > C\Big) < \frac{\varepsilon}{2}. \tag{1}$$

And we have

$$P\Big(\min_{\tilde{K},\tilde{\rho}_{Xg}}\sum_{g\in B_1^c}\left(\frac{(r_{Xg}-\tilde{\rho}_{Xg})^2}{\mathrm{SE}(r_{Xg})^2}+\frac{(r_{Yg}-\tilde{K}\tilde{\rho}_{Xg})^2}{\mathrm{SE}(r_{Yg})^2}\right) \le \sum_{g\in(B^0_{XY})^c}\left(\frac{(r_{Xg}-\rho_{Xg})^2}{\mathrm{SE}(r_{Xg})^2}+\frac{(r_{Yg}-K_{XY}\rho_{Xg})^2}{\mathrm{SE}(r_{Yg})^2}\right)\Big)$$
$$\le P\Big(\min_{\tilde{K},\tilde{\rho}_{Xg}}\sum_{g\in B_1^c}\left(\frac{(r_{Xg}-\tilde{\rho}_{Xg})^2}{\mathrm{SE}(r_{Xg})^2}+\frac{(r_{Yg}-\tilde{K}\tilde{\rho}_{Xg})^2}{\mathrm{SE}(r_{Yg})^2}\right) \le C\Big) + P\Big(\sum_{g\in(B^0_{XY})^c}\left(\frac{(r_{Xg}-\rho_{Xg})^2}{\mathrm{SE}(r_{Xg})^2}+\frac{(r_{Yg}-K_{XY}\rho_{Xg})^2}{\mathrm{SE}(r_{Yg})^2}\right) > C\Big).$$

After profiling out $\tilde{\rho}_{Xg}$'s, we get

$$\min_{\tilde{K},\tilde{\rho}_{Xg}}\sum_{g\in B_1^c}\left(\frac{(r_{Xg}-\tilde{\rho}_{Xg})^2}{\mathrm{SE}(r_{Xg})^2}+\frac{(r_{Yg}-\tilde{K}\tilde{\rho}_{Xg})^2}{\mathrm{SE}(r_{Yg})^2}\right) = \min_{\tilde{K}}\sum_{g\in B_1^c}\frac{(r_{Yg}-\tilde{K}\cdot r_{Xg})^2}{\mathrm{SE}(r_{Yg})^2+\tilde{K}^2\mathrm{SE}(r_{Xg})^2},$$

so

$$P\Big(\min_{\tilde{K},\tilde{\rho}_{Xg}}\sum_{g\in B_1^c}\left(\frac{(r_{Xg}-\tilde{\rho}_{Xg})^2}{\mathrm{SE}(r_{Xg})^2}+\frac{(r_{Yg}-\tilde{K}\tilde{\rho}_{Xg})^2}{\mathrm{SE}(r_{Yg})^2}\right) \le C\Big)$$
$$= P\Big(\min_{\tilde{K}}\sum_{g\in B_1^c}\frac{(r_{Yg}-\tilde{K}\cdot r_{Xg})^2}{\mathrm{SE}(r_{Yg})^2+\tilde{K}^2\mathrm{SE}(r_{Xg})^2} \le C\Big).$$

We have $\frac{r_{Yg}-\tilde{K}\cdot r_{Xg}}{\sqrt{\mathrm{SE}(r_{Yg})^2+\tilde{K}^2\mathrm{SE}(r_{Xg})^2}} \sim N\big(\frac{K_{XY}\cdot\rho_{Xg}+b_{XYg}-\tilde{K}\cdot\rho_{Xg}}{\sqrt{\mathrm{SE}(r_{Yg})^2+\tilde{K}^2\mathrm{SE}(r_{Xg})^2}},1\big)$, so $\sum_{g\in B_1^c}\frac{(r_{Yg}-\tilde{K}\cdot r_{Xg})^2}{\mathrm{SE}(r_{Yg})^2+\tilde{K}^2\mathrm{SE}(r_{Xg})^2}$ follows non-central $\chi^2$ distribution with degrees of freedom $(m-m^0_{XY})$ and non-centrality parameter $\lambda_{\tilde{K}}$ depending on $\tilde{K}$

$$\lambda_{\tilde{K}} = \sum_{g\in B_1^c}\frac{(K_{XY}\cdot\rho_{Xg}+b_{XYg}-\tilde{K}\cdot\rho_{Xg})^2}{\mathrm{SE}(r_{Yg})^2+\tilde{K}^2\mathrm{SE}(r_{Xg})^2}.$$

With Assumption 2, we get

$$\lambda_{\tilde{K}} \ge \sum_{g\in B_1^c}\frac{(K_{XY}\cdot\rho_{Xg}+b_{XYg}-\tilde{K}\cdot\rho_{Xg})^2}{\frac{u_Y}{N_2}+\tilde{K}^2\cdot\frac{u_X}{l_n\cdot N_2}} = N_2\cdot\sum_{g\in B_1^c}\frac{(K_{XY}\cdot\rho_{Xg}+b_{XYg}-\tilde{K}\cdot\rho_{Xg})^2}{u_Y+\tilde{K}^2\cdot\frac{u_X}{l_n}}.$$

With Assumption 1, we know

$$\min_{\tilde{K}}\sum_{g\in B_1^c}\frac{(K_{XY}\cdot\rho_{Xg}+b_{XYg}-\tilde{K}\cdot\rho_{Xg})^2}{u_Y+\tilde{K}^2\cdot\frac{u_X}{l_n}} = v > 0,$$

here $v$ is a constant. This is because, with Assumption 1, there is no $\tilde{K}$ making $K_{XY}\cdot\rho_{Xg}+b_{XYg}-\tilde{K}\cdot\rho_{Xg}=0$ for all $g\in B_1^c$ simultaneously. So we have $\min_{\tilde{K}}\lambda_{\tilde{K}} \ge N_2\cdot v$. Then as $N_2$ large enough, we have

$$P\Big(\min_{\tilde{K}}\sum_{g\in B_1^c}\frac{(r_{Yg}-\tilde{K}\cdot r_{Xg})^2}{\mathrm{SE}(r_{Yg})^2+\tilde{K}^2\mathrm{SE}(r_{Xg})^2} \le C\Big) \le \frac{\varepsilon}{2}. \tag{2}$$

Combining (1) and (2), we get $P(\hat{B}_{XY}(m_{XY}^0) = B_{XY}^0) \to 1$ as $N_1, N_2 \to \infty$.

Next, we show $P(\hat{m}_I = m_{XY}^0) \to 1$. For any $m_1 < m_{XY}^0$, we have

$$
\begin{aligned}
&P(\hat{m}_I = m_1) \le P\left(\mathrm{BIC}(m_1) \le \mathrm{BIC}(m_{XY}^0)\right) \\
&= P\left(-2\cdot L\left(\hat{K}_{XY}(m_1), \hat{\rho}_{Xg}(m_1), \hat{b}_{XYg}(m_1)\right) + \log(n)\cdot m_1 \le -2\cdot L\left(\hat{K}_{XY}(m_{XY}^0), \hat{\rho}_{Xg}(m_{XY}^0), \hat{b}_{XYg}(m_{XY}^0)\right) + \log(n)\cdot m_{XY}^0\right) \\
&= P\left(2\cdot L\left(\hat{K}_{XY}(m_{XY}^0), \hat{\rho}_{Xg}(m_{XY}^0), \hat{b}_{XYg}(m_{XY}^0)\right) - 2\cdot L\left(\hat{K}_{XY}(m_1), \hat{\rho}_{Xg}(m_1), \hat{b}_{XYg}(m_1)\right) \le \log(n)(m_{XY}^0 - m_1)\right).
\end{aligned}
$$

As we have shown $P(\hat{B}_{XY}(m_{XY}^0) = B_{XY}^0) \to 1$, with probability goes to 1 we have

$$
\begin{aligned}
&2\cdot L\left(\hat{K}_{XY}(m_{XY}^0), \hat{\rho}_{Xg}(m_{XY}^0), \hat{b}_{XYg}(m_{XY}^0)\right) - 2\cdot L\left(\hat{K}_{XY}(m_1), \hat{\rho}_{Xg}(m_1), \hat{b}_{XYg}(m_1)\right) \\
&= \min_{\tilde{K}, \tilde{\rho}_{Xg}} \sum_{g\in \hat{B}_{m_1}^c} \left(\frac{(r_{Xg} - \tilde{\rho}_{Xg})^2}{\mathrm{SE}(r_{Xg})^2} + \frac{(r_{Yg} - \tilde{K}\tilde{\rho}_{Xg})^2}{\mathrm{SE}(r_{Yg})^2}\right) - \min_{\tilde{K}, \tilde{\rho}_{Xg}} \sum_{g\in (B_{XY}^0)^c} \left(\frac{(r_{Xg} - \tilde{\rho}_{Xg})^2}{\mathrm{SE}(r_{Xg})^2} + \frac{(r_{Yg} - \tilde{K}\tilde{\rho}_{Xg})^2}{\mathrm{SE}(r_{Yg})^2}\right) \\
&\ge \min_{\tilde{K}, \tilde{\rho}_{Xg}} \sum_{g\in \hat{B}_{m_1}^c} \left(\frac{(r_{Xg} - \tilde{\rho}_{Xg})^2}{\mathrm{SE}(r_{Xg})^2} + \frac{(r_{Yg} - \tilde{K}\tilde{\rho}_{Xg})^2}{\mathrm{SE}(r_{Yg})^2}\right) - \sum_{g\in (B_{XY}^0)^c} \left(\frac{(r_{Xg} - \rho_{Xg})^2}{\mathrm{SE}(r_{Xg})^2} + \frac{(r_{Yg} - K_{XY}\rho_{Xg})^2}{\mathrm{SE}(r_{Yg})^2}\right).
\end{aligned}
$$

Then we get

$$
\begin{aligned}
&P(\hat{m}_I = m_1) \\
&\le P\left(\min_{\tilde{K}, \tilde{\rho}_{Xg}} \sum_{g\in \hat{B}_{m_1}^c} \left(\frac{(r_{Xg} - \tilde{\rho}_{Xg})^2}{\mathrm{SE}(r_{Xg})^2} + \frac{(r_{Yg} - \tilde{K}\tilde{\rho}_{Xg})^2}{\mathrm{SE}(r_{Yg})^2}\right) \le \sum_{g\in (B_{XY}^0)^c} \left(\frac{(r_{Xg} - \rho_{Xg})^2}{\mathrm{SE}(r_{Xg})^2} + \frac{(r_{Yg} - K_{XY}\rho_{Xg})^2}{\mathrm{SE}(r_{Yg})^2}\right) + \log(n)(m_{XY}^0 - m_1)\right) \\
&\le \sum_{|B| = m_1} P\left(\min_{\tilde{K}, \tilde{\rho}_{Xg}} \sum_{g\in B^c} \left(\frac{(r_{Xg} - \tilde{\rho}_{Xg})^2}{\mathrm{SE}(r_{Xg})^2} + \frac{(r_{Yg} - \tilde{K}\tilde{\rho}_{Xg})^2}{\mathrm{SE}(r_{Yg})^2}\right) \le \sum_{g\in (B_{XY}^0)^c} \left(\frac{(r_{Xg} - \rho_{Xg})^2}{\mathrm{SE}(r_{Xg})^2} + \frac{(r_{Yg} - K_{XY}\rho_{Xg})^2}{\mathrm{SE}(r_{Yg})^2}\right) + \log(n)(m_{XY}^0 - m_1).\right)
\end{aligned}
$$

Similar as above, we get

$$
\min_{\tilde{K}, \tilde{\rho}_{Xg}} \sum_{g\in B^c} \left(\frac{(r_{Xg} - \tilde{\rho}_{Xg})^2}{\mathrm{SE}(r_{Xg})^2} + \frac{(r_{Yg} - \tilde{K}\tilde{\rho}_{Xg})^2}{\mathrm{SE}(r_{Yg})^2}\right) = \min_{\tilde{K}} \sum_{g\in B^c} \frac{(r_{Yg} - \tilde{K}\cdot r_{Xg})^2}{\mathrm{SE}(r_{Yg})^2 + \tilde{K}^2\mathrm{SE}(r_{Xg})^2},
$$

and $\sum_{g\in B^c} \frac{(r_{Yg} - \tilde{K}\cdot r_{Xg})^2}{\mathrm{SE}(r_{Yg})^2 + \tilde{K}^2\mathrm{SE}(r_{Xg})^2}$ follows non-central $\chi^2$ distribution with degrees of freedom $(m - m_1)$ and non-centrality parameter $\lambda_{\tilde{K}}$ depending on $\tilde{K}$

$$
\lambda_{\tilde{K}} = \sum_{g\in B^c} \frac{(K_{XY}\cdot \rho_{Xg} + b_{XYg} - \tilde{K}\cdot \rho_{Xg})^2}{\mathrm{SE}(r_{Yg})^2 + \tilde{K}^2\mathrm{SE}(r_{Xg})^2}.
$$

Similarly, since $m_1 < m_{XY}^0$, with Assumption 2 we have $\lambda_{\tilde{K}} \ge N_2\cdot v$ for some constant $v$, so for any $|B| = m_1$, we get

$$
P\left(\min_{\tilde{K}, \tilde{\rho}_{Xg}} \sum_{g\in B^c} \left(\frac{(r_{Xg} - \tilde{\rho}_{Xg})^2}{\mathrm{SE}(r_{Xg})^2} + \frac{(r_{Yg} - \tilde{K}\tilde{\rho}_{Xg})^2}{\mathrm{SE}(r_{Yg})^2}\right) \le \sum_{g\in (B_{XY}^0)^c} \left(\frac{(r_{Xg} - \rho_{Xg})^2}{\mathrm{SE}(r_{Xg})^2} + \frac{(r_{Yg} - K_{XY}\rho_{Xg})^2}{\mathrm{SE}(r_{Yg})^2}\right) + \log(n)(m_{XY}^0 - m_1)\right) \to 0.
$$

This gives us $P(\hat{m}_I = m_1) \to 0$ for any $m_1 < m_{XY}^0$. For any $m_1 > m_{XY}^0$, we have

$$
\begin{aligned}
&P(\hat{m}_I = m_1) \\
&\le P\left(\log(n)(m_1 - m_{XY}^0) \le \sum_{g\in (B_{XY}^0)^c} \left(\frac{(r_{Xg} - \rho_{Xg})^2}{\mathrm{SE}(r_{Xg})^2} + \frac{(r_{Yg} - K_{XY}\rho_{Xg})^2}{\mathrm{SE}(r_{Yg})^2}\right) - \min_{\tilde{K}, \tilde{\rho}_{Xg}} \sum_{g\in \hat{B}_{m_1}^c} \left(\frac{(r_{Xg} - \tilde{\rho}_{Xg})^2}{\mathrm{SE}(r_{Xg})^2} + \frac{(r_{Yg} - \tilde{K}\tilde{\rho}_{Xg})^2}{\mathrm{SE}(r_{Yg})^2}\right)\right) \\
&\le P\left(\log(n)(m_1 - m_{XY}^0) \le \sum_{g\in (B_{XY}^0)^c} \left(\frac{(r_{Xg} - \rho_{Xg})^2}{\mathrm{SE}(r_{Xg})^2} + \frac{(r_{Yg} - K_{XY}\rho_{Xg})^2}{\mathrm{SE}(r_{Yg})^2}\right)\right)
\end{aligned}
$$

Since $\sum_{g\in(B^0_{XY})^c}\left(\frac{(r_{Xg}-\rho_{Xg})^2}{\mathrm{SE}(r_{Xg})^2}+\frac{(r_{Yg}-K_{XY}\rho_{Xg})^2}{\mathrm{SE}(r_{Yg})^2}\right)$ is a central $\chi^2$ distribution with degrees of freedom $2(m-m^0_{XY})$, we get $P(\hat{m}_I=m_1)\to 0$ for any $m_1>m^0_{XY}$. So we have $P(\hat{m}_I=m^0_{XY})\to 1$ as $N_1,N_2\to\infty$.

As $P(\hat{B}_{XY}(\hat{m}_I)=B^0_{XY})\to 1$, we could consistently select all invalid IVs. Following Theorem 3.2 in [4], we have

$$\frac{V}{\sqrt{V_1}}(\hat{K}_{XY}-K_{XY})\xrightarrow{d}N(0,1),\ \text{as}\ N_1,N_2\to\infty,$$

where

$$V=\sum_{g\in(B^0_{XY})^c}\frac{\rho^2_{Xg}\sigma^2_{Xg}+\rho^2_{Yg}\sigma^2_{Yg}}{(\sigma^2_{Xg}\cdot K^2_{XY}+\sigma^2_{Yg})^2}=\sum_{g\in(B^0_{XY})^c}\frac{\rho^2_{Xg}}{\sigma^2_{Xg}\cdot K^2_{XY}+\sigma^2_{Yg}},$$

and

$$V_1=\sum_{g\in(B^0_{XY})^c}\frac{\rho^2_{Xg}\sigma^2_{Xg}+\rho^2_{Yg}\sigma^2_{Yg}+\sigma^2_{Xg}\sigma^2_{Yg}}{(\sigma^2_{Xg}\cdot K^2_{XY}+\sigma^2_{Yg})^2}.$$

In our model $\rho_{Xg}$'s and $\rho_{Yg}$'s are fixed constants, $\sigma^2_{Xg}$'s and $\sigma^2_{Yg}$'s are $O(1/n)$, so we have $V/V_1\to 1$, and

$$\sqrt{V}(\hat{K}_{XY}-K_{XY})\xrightarrow{d}N(0,1),\ \text{as}\ N_1,N_2\to\infty.$$

□

## S3.2 Proof of Theorem 2

First we introduce the definition of "converge weakly", as Definition 2.2 in [2].

**Definition 2.2 by Xiong et al. [2].** *$F(\cdot)$ is a distribution function, $F_n(\cdot)$ is random distribution function that depends on some random variable. We say $F_n(\cdot)$ converges weakly to $F(\cdot)$ in probability if for each continuous point $x$ of $F(\cdot)$, $F_n(x)\xrightarrow{p}F(x)$ as $n\to\infty$. This is denoted by $F_n(\cdot)\xrightarrow{w.P}F(\cdot)$.*

Now we show the proof of Theorem 2.

**Theorem 2.** *Under Assumptions 1 and 2, conditional on the original GWAS summary data, $\sqrt{V}(\hat{K}^{(t)}_{XY}-\hat{K}_{XY})\xrightarrow{w.P.}N(0,1)$ as $N_1,N_2\to\infty$.*

*Proof.* Denote $\tilde{B}=\{i:\tilde{b}_{XYg}\neq 0\}$ as the set of estimated invalid IVs with non-zero direct effects based on perturbed data. First we show that $P(\tilde{B}=B^0_{XY}|\mathscr{D})\xrightarrow{p}1$, which is equivalent to for any $\varepsilon>0,\delta>0$, there exists $n$ such that when $n_1>n,n_2>n$ we have $P\left(P(\tilde{B}=B^0_{XY}|\mathscr{D})<1-\varepsilon\right)<\delta$. Following similar argument in Theorem 1, we could get the unconditional probability $P(\tilde{B}=B^0_{XY})\to 1$. Suppose we could find a pair of $\varepsilon_0>0,\delta_0>0$ such that $P\left(P(\tilde{B}=B^0_{XY}|\mathscr{D})<1-\varepsilon_0\right)>\delta_0$ for arbitrarily large $n_1,n_2$, then we can get

$$P(\tilde{B}=B^0_{XY})=\int_{\mathscr{D}}P(\tilde{B}=B^0_{XY}|\mathscr{D})dF(\mathscr{D})<1-\varepsilon_0\delta_0,$$

contradicts that $P(\tilde{B}=B^0_{XY})\to 1$, thus we have shown that $P(\tilde{B}=B^0_{XY}|\mathscr{D})\xrightarrow{p}1$. Now we could focus on the case that $\tilde{B}=\hat{B}=B^0_{XY}$, for simplicity we use $\tilde{K}$, $\hat{K}$ to represent $\hat{K}^{(t)}_{XY}$, $\hat{K}_{XY}$. Similar to [4], after profiling out $\rho_{Xg}$'s in the original log-likelihood function, we have

$$\tilde{K}=\arg\min_K\sum_{g\in(B^0_{XY})^c}\frac{(\tilde{r}_{Yg}-K\cdot\tilde{r}_{Xg})^2}{\sigma^2_{Xg}\cdot K^2+\sigma^2_{Yg}},\quad \hat{K}=\arg\min_K\sum_{g\in(B^0_{XY})^c}\frac{(r_{Yg}-K\cdot r_{Xg})^2}{\sigma^2_{Xg}\cdot K^2+\sigma^2_{Yg}}. \tag{3}$$

Denote

$$f(K)=\sum_{g\in(B^0_{XY})^c}\frac{(\tilde{r}_{Yg}-K\cdot\tilde{r}_{Xg})^2}{\sigma^2_{Xg}\cdot K^2+\sigma^2_{Yg}},$$

and

$$\begin{aligned}\phi(K)=\frac{\partial f(K)}{\partial K}&=\sum_{g\in(B^0_{XY})^c}\frac{(\tilde{r}_{Yg}-K\tilde{r}_{Xg})(K\tilde{r}_{Yg}\sigma^2_{Xg}+\tilde{r}_{Xg}\sigma^2_{Yg})}{(\sigma^2_{Xg}K^2+\sigma^2_{Yg})^2}\\&=\sum_{g\in(B^0_{XY})^c}\frac{(r_{Yg}-Kr_{Xg})(Kr_{Yg}\sigma^2_{Xg}+r_{Xg}\sigma^2_{Yg})+(r_{Yg}-Kr_{Xg})(K\xi_g\sigma^2_{Xg}+\varepsilon_g\sigma^2_{Yg})+(\xi_g-K\varepsilon_g)(Kr_{Yg}\sigma^2_{Xg}+r_{Xg}\sigma^2_{Yg}+K\xi_g\sigma^2_{Xg}+\varepsilon_g\sigma^2_{Yg})}{(\sigma^2_{Xg}K^2+\sigma^2_{Yg})^2},\end{aligned}$$

here $\xi_g=\tilde{r}_{Yg}-r_{Yg}\sim N(0,\sigma^2_{Yg})$, $\varepsilon_g=\tilde{r}_{Xg}-r_{Xg}\sim N(0,\sigma^2_{Xg})$. We have

$$0=\phi(\tilde{K})=\phi(\hat{K})+\phi'(\hat{K})(\tilde{K}-\hat{K})+\frac{1}{2}\phi''(K^*)(\tilde{K}-\hat{K})^2,$$

with $K^*$ is between $\tilde{K}$ and $\hat{K}$, thus

$$\sqrt{V}(\tilde{K}-\hat{K})=\frac{-\phi(\hat{K})/\sqrt{V}}{\phi'(\hat{K})/V+(1/2)(\tilde{K}-\hat{K})\phi''(K^*)/V}.$$

Next we show $\phi(\hat{K})/\sqrt{V}|\mathscr{D}\xrightarrow{w.P}N(0,1)$. From equation (6), we can get

$$\phi(\hat{K})=\sum_{g\in(B^0_{XY})^c}\frac{(r_{Yg}-\hat{K}r_{Xg})(\hat{K}\xi_g\sigma^2_{Xg}+\varepsilon_g\sigma^2_{Yg})+(\xi_g-\hat{K}\varepsilon_g)(\hat{K}r_{Yg}\sigma^2_{Xg}+r_{Xg}\sigma^2_{Yg}+\hat{K}\xi_g\sigma^2_{Xg}+\varepsilon_g\sigma^2_{Yg})}{(\sigma^2_{Xg}\hat{K}^2+\sigma^2_{Yg})^2}.$$

Note that $\xi_g$'s and $\varepsilon_g$'s are $O_p(1/\sqrt{n})$, $n=\min(N_1,N_2)$, thus

$$\phi(\hat{K})=\sum_{g\in(B^0_{XY})^c}\frac{\xi_g(r_{Yg}\hat{K}\sigma^2_{Xg}-\hat{K}^2r_{Xg}\sigma^2_{Xg}+\hat{K}r_{Yg}\sigma^2_{Xg}+r_{Xg}\sigma^2_{Yg})+\varepsilon_g(r_{Yg}\sigma^2_{Yg}-\hat{K}r_{Xg}\sigma^2_{Yg}-\hat{K}^2r_{Yg}\sigma^2_{Xg}-\hat{K}r_{Xg}\sigma^2_{Yg})}{(\sigma^2_{Xg}\hat{K}^2+\sigma^2_{Yg})^2}+O_p(1), \tag{4}$$

thus $\phi(\hat{K})/\sqrt{V}|\mathscr{D}=N(0,V^*/V)|\mathscr{D}+O_p(1/\sqrt{n})$, with

$$V^*=\sum_{g\in(B^0_{XY})^c}\frac{\sigma^2_{Yg}(r_{Yg}\hat{K}\sigma^2_{Xg}-\hat{K}^2r_{Xg}\sigma^2_{Xg}+\hat{K}r_{Yg}\sigma^2_{Xg}+r_{Xg}\sigma^2_{Yg})^2+\sigma^2_{Xg}(r_{Yg}\sigma^2_{Yg}-\hat{K}r_{Xg}\sigma^2_{Yg}-\hat{K}^2r_{Yg}\sigma^2_{Xg}-\hat{K}r_{Xg}\sigma^2_{Yg})^2}{(\sigma^2_{Xg}\hat{K}^2+\sigma^2_{Yg})^4},$$

as $r_{Xg}\xrightarrow{p}\rho_{Xi}$, $r_{Yg}\xrightarrow{p}\rho_{Yi}$, $\hat{K}\xrightarrow{p}K_0$, we can get $V^*/V\xrightarrow{p}1$, thus we get $\phi(\hat{K})/\sqrt{V}|\mathscr{D}\xrightarrow{w.P}N(0,1)$.

Next we show $-\phi'(\hat{K})/V|\mathscr{D}\xrightarrow{w.P}1$. After some calculation we get

$$\phi'(K)=\sum_{g\in(B^0_{XY})^c}\frac{2\sigma^4_{Xg}\tilde{r}_{Xg}\tilde{r}_{Yg}\cdot K^3+3(\sigma^2_{Xg}\sigma^2_{Yg}\tilde{r}^2_{Xg}-\sigma^4_{Xg}\tilde{r}^2_{Yg})K^2-6\sigma^2_{Xg}\sigma^2_{Yg}\tilde{r}_{Xg}\tilde{r}_{Yg}K+(\sigma^2_{Xg}\sigma^2_{Yg}\tilde{r}^2_{Yg}-\tilde{r}^2_{Xg}\sigma^4_{Yg})}{(\sigma^2_{Xg}K^2+\sigma^2_{Yg})^3}, \tag{5}$$

as $\tilde{r}_{Xg} \xrightarrow{p} \rho_{Xi}$, $\tilde{r}_{Yg} \xrightarrow{p} \rho_{Yi}$, $\hat{K} \xrightarrow{p} K_0$, we get $-\phi'(\hat{K})/V \xrightarrow{p} 1$, with Theorem 3.3 in [2], $-\phi'(\hat{K})/V|\mathscr{D} \xrightarrow{w.P} 1$.

Based on equation (8), we can see $\phi''(K)$ has its numerator of order $n^5$ and its denominator of order $n^6$, thus $\phi''(K^*)/V = O_p(1)$. As $\tilde{K} \xrightarrow{p} K_0$, $\hat{K} \xrightarrow{p} K_0$, we have $\tilde{K} - \hat{K} \xrightarrow{p} 0$, again with Theorem 3.3 in [2] we get $\tilde{K} - \hat{K}|\mathscr{D} \xrightarrow{w.P} 0$. Thus we can get $\frac{1}{2}\phi''(K^*)(\tilde{K} - \hat{K})|\mathscr{D} \xrightarrow{w.P} 0$. Now with Theorem 3.2 in [2], we can get $\sqrt{V}(\tilde{K} - \hat{K})|\mathscr{D} \xrightarrow{w.P} N(0,1)$, completing the proof. □

## S3.3 MR-cML with Data Perturbation

Now we show that the data perturbation scheme is also consistent for MR-cML in [3]. We use the following notations: the true effects on $X$ are $\beta_{Xi}$'s, and those on $Y$ are $\beta_{Yi}$'s; the estimated/observed effects on $X$ are $\hat{\beta}_{Xi} \sim N(\beta_{Xi}, \sigma_{Xi}^2)$, and those on $Y$ are $\hat{\beta}_{Yi} \sim N(\beta_{Yi}, \sigma_{Yi}^2)$. Here $\sigma_{Xi}$'s and $\sigma_{Yi}$'s are the true standard deviations; in practice we have the standard errors $\hat{\sigma}_{Xi}$'s and $\hat{\sigma}_{Yi}$'s as their estimates from GWAS datasets, thus approximately we have $\hat{\beta}_{Xi} \sim N(\beta_{Xi}, \hat{\sigma}_{Xi}^2)$ and $\hat{\beta}_{Yi} \sim N(\beta_{Yi}, \hat{\sigma}_{Yi}^2)$. For simplicity and without ambiguity, we treat the standard errors $\hat{\sigma}_{Xi}$'s and $\hat{\sigma}_{Yi}$'s as the true values of $\sigma_{Xi}$'s and $\sigma_{Yi}$'s in the following. The perturbed effects on $X$ are $\tilde{\beta}_{Xi} \sim N(\hat{\beta}_{Xi}, \sigma_{Xi}^2)$, and the perturbed effects on $Y$ are $\tilde{\beta}_{Yi} \sim N(\hat{\beta}_{Yi}, \sigma_{Yi}^2)$. The true causal effect is $\theta_0$, the estimated causal effect based on the observed data with cML-BIC is $\hat{\theta}$, and the estimated causal effect based on a perturbed dataset with cML-BIC is $\tilde{\theta}$. Let $\mathscr{D} = \{(\hat{\beta}_{Xi}, \hat{\beta}_{Yi})|i = 1, \cdots, m\}$ denote the observed data.

**Assumption 1 for MR-cML.** *(Plurality condition.) Suppose that $B_0$ is the index set of the invalid IVs with non-zero direct effects, i.e. $r_i \neq 0$ if and only if $i \in B_0$, and $K_0 = |B_0|$. For any $B \subseteq \{1, \cdots, m\}$ and $|B| = K_0$, if $B \neq B_0$, then there does not exist any constant $S$ such that $r_i = S \cdot \beta_{Xi}$ for all $i \in B^c$.*

**Assumption 2 for MR-cML.** *(Orders of the variances and sample sizes.) There exist positive constants $l_X, l_Y, l_N$ and $u_X, u_Y, u_N$ such that we have $l_X/n_1 \leq \sigma_{Xi}^2 \leq u_X/n_1$, $l_Y/n_2 \leq \sigma_{Yi}^2 \leq u_Y/n_2$, and $l_N \cdot n_2 \leq n_1 \leq u_N \cdot n_2$ for $i = 1, \cdots, m$.*

Denote

$$V = \sum_{i \in B_0^c} \frac{\beta_{Xi}^2}{\sigma_{Xi}^2 \cdot \theta_0^2 + \sigma_{Yi}^2}.$$

**Theorem 2 for MR-cML.** *Under Assumptions 1 for MR-cML and 2 for MR-cML, according to Definition 2.2 in [2], $\sqrt{V}(\tilde{\theta} - \hat{\theta})|\mathscr{D} \xrightarrow{w.P} N(0,1)$ as $n_1, n_2 \to \infty$.*

*Proof.* Denote $\tilde{B} = \{i : \tilde{r}_i \neq 0\}$ as the set of estimated invalid IVs with non-zero direct effects based on perturbed data. First we show that $P(\tilde{B} = B_0|\mathscr{D}) \xrightarrow{p} 1$, which is equivalent to for any $\varepsilon > 0, \delta > 0$, there exists $n$ such that when $n_1 > n, n_2 > n$ we have $P\left(P(\tilde{B} = B_0|\mathscr{D}) < 1 - \varepsilon\right) < \delta$. Following similar argument in Theorem 1, we could get the unconditional probability $P(\tilde{B} = B_0) \to 1$. Suppose we could find a pair of $\varepsilon_0 > 0, \delta_0 > 0$ such that $P\left(P(\tilde{B} = B_0|\mathscr{D}) < 1 - \varepsilon_0\right) > \delta_0$ for arbitrarily large $n_1, n_2$, then we can get

$$P(\tilde{B} = B_0) = \int_{\mathscr{D}} P(\tilde{B} = B_0|\mathscr{D}) dF(\mathscr{D}) < 1 - \varepsilon_0 \delta_0,$$

contradicts that $P(\tilde{B}=B_0)\to 1$, thus we have shown that $P(\tilde{B}=B_0|\mathscr{D})\xrightarrow{p}1$. Now we could focus on the case that $\tilde{B}=\hat{B}=B_0$. Similar to [4], after profiling out $b_{Xi}$'s in the original log-likelihood function, we have

$$\tilde{\theta}=\arg\min_\theta\sum_{i\in B_0^c}\frac{(\tilde{\beta}_{Yi}-\theta\cdot\tilde{\beta}_{Xi})^2}{\sigma_{Xi}^2\cdot\theta^2+\sigma_{Yi}^2},\quad \hat{\theta}=\arg\min_\theta\sum_{i\in B_0^c}\frac{(\hat{\beta}_{Yi}-\theta\cdot\hat{\beta}_{Xi})^2}{\sigma_{Xi}^2\cdot\theta^2+\sigma_{Yi}^2}. \tag{6}$$

Denote

$$f(\theta)=\sum_{i\in B_0^c}\frac{(\tilde{\beta}_{Yi}-\theta\cdot\tilde{\beta}_{Xi})^2}{\sigma_{Xi}^2\cdot\theta^2+\sigma_{Yi}^2},$$

and

$$\begin{aligned}\phi(\theta)&=\frac{\partial f(\theta)}{\partial\theta}=\sum_{i\in B_0^c}\frac{(\tilde{\beta}_{Yi}-\theta\tilde{\beta}_{Xi})(\theta\tilde{\beta}_{Yi}\sigma_{Xi}^2+\tilde{\beta}_{Xi}\sigma_{Yi}^2)}{(\sigma_{Xi}^2\theta^2+\sigma_{Yi}^2)^2}\\ &=\sum_{i\in B_0^c}\frac{(\hat{\beta}_{Yi}-\theta\hat{\beta}_{Xi})(\theta\hat{\beta}_{Yi}\sigma_{Xi}^2+\hat{\beta}_{Xi}\sigma_{Yi}^2)+(\hat{\beta}_{Yi}-\theta\hat{\beta}_{Xi})(\theta\xi_i\sigma_{Xi}^2+\varepsilon_i\sigma_{Yi}^2)+(\xi_i-\theta\varepsilon_i)(\theta\hat{\beta}_{Yi}\sigma_{Xi}^2+\hat{\beta}_{Xi}\sigma_{Yi}^2+\theta\xi_i\sigma_{Xi}^2+\varepsilon_i\sigma_{Yi}^2)}{(\sigma_{Xi}^2\theta^2+\sigma_{Yi}^2)^2},\end{aligned}$$

here $\xi_i=\tilde{\beta}_{Yi}-\hat{\beta}_{Yi}\sim N(0,\sigma_{Yi}^2)$, $\varepsilon_i=\tilde{\beta}_{Xi}-\hat{\beta}_{Xi}\sim N(0,\sigma_{Xi}^2)$. We have

$$0=\phi(\tilde{\theta})=\phi(\hat{\theta})+\phi'(\hat{\theta})(\tilde{\theta}-\hat{\theta})+\frac{1}{2}\phi''(\theta^*)(\tilde{\theta}-\hat{\theta})^2,$$

with $\theta^*$ is between $\tilde{\theta}$ and $\hat{\theta}$, thus

$$\sqrt{V}(\tilde{\theta}-\hat{\theta})=\frac{-\phi(\hat{\theta})/\sqrt{V}}{\phi'(\hat{\theta})/V+(1/2)(\tilde{\theta}-\hat{\theta})\phi''(\theta^*)/V}.$$

Next we show $\phi(\hat{\theta})/\sqrt{V}|\mathscr{D}\xrightarrow{w.P}N(0,1)$. From equation (6), we can get

$$\phi(\hat{\theta})=\sum_{i\in B_0^c}\frac{(\hat{\beta}_{Yi}-\hat{\theta}\hat{\beta}_{Xi})(\hat{\theta}\xi_i\sigma_{Xi}^2+\varepsilon_i\sigma_{Yi}^2)+(\xi_i-\hat{\theta}\varepsilon_i)(\hat{\theta}\hat{\beta}_{Yi}\sigma_{Xi}^2+\hat{\beta}_{Xi}\sigma_{Yi}^2+\hat{\theta}\xi_i\sigma_{Xi}^2+\varepsilon_i\sigma_{Yi}^2)}{(\sigma_{Xi}^2\hat{\theta}^2+\sigma_{Yi}^2)^2}.$$

Note that $\xi_i$'s and $\varepsilon_i$'s are $O_p(1/\sqrt{n})$, $n=\min(n_1,n_2)$, thus

$$\phi(\hat{\theta})=\sum_{i\in B_0^c}\frac{\xi_i(\hat{\beta}_{Yi}\hat{\theta}\sigma_{Xi}^2-\hat{\theta}^2\hat{\beta}_{Xi}\sigma_{Xi}^2+\hat{\theta}\hat{\beta}_{Yi}\sigma_{Xi}^2+\hat{\beta}_{Xi}\sigma_{Yi}^2)+\varepsilon_i(\hat{\beta}_{Yi}\sigma_{Yi}^2-\hat{\theta}\hat{\beta}_{Xi}\sigma_{Yi}^2-\hat{\theta}^2\hat{\beta}_{Yi}\sigma_{Xi}^2-\hat{\theta}\hat{\beta}_{Xi}\sigma_{Yi}^2)}{(\sigma_{Xi}^2\hat{\theta}^2+\sigma_{Yi}^2)^2}+O_p(1), \tag{7}$$

thus $\phi(\hat{\theta})/\sqrt{V}|\mathscr{D}=N(0,V^*/V)|\mathscr{D}+O_p(1/\sqrt{n})$, with

$$V^*=\sum_{i\in B_0^c}\frac{\sigma_{Yi}^2(\hat{\beta}_{Yi}\hat{\theta}\sigma_{Xi}^2-\hat{\theta}^2\hat{\beta}_{Xi}\sigma_{Xi}^2+\hat{\theta}\hat{\beta}_{Yi}\sigma_{Xi}^2+\hat{\beta}_{Xi}\sigma_{Yi}^2)^2+\sigma_{Xi}^2(\hat{\beta}_{Yi}\sigma_{Yi}^2-\hat{\theta}\hat{\beta}_{Xi}\sigma_{Yi}^2-\hat{\theta}^2\hat{\beta}_{Yi}\sigma_{Xi}^2-\hat{\theta}\hat{\beta}_{Xi}\sigma_{Yi}^2)^2}{(\sigma_{Xi}^2\hat{\theta}^2+\sigma_{Yi}^2)^4},$$

as $\hat{\beta}_{Xi}\xrightarrow{p}\beta_{Xi}$, $\hat{\beta}_{Yi}\xrightarrow{p}\beta_{Yi}$, $\hat{\theta}\xrightarrow{p}\theta_0$, we can get $V^*/V\xrightarrow{p}1$, thus we get $\phi(\hat{\theta})/\sqrt{V}|\mathscr{D}\xrightarrow{w.P}N(0,1)$.

Next we show $-\phi'(\hat{\theta})/V|\mathscr{D}\xrightarrow{w.P}1$. After some calculation we get

$$\phi'(\theta)=\sum_{i\in B_0^c}\frac{2\sigma_{Xi}^4\tilde{\beta}_{Xi}\tilde{\beta}_{Yi}\cdot\theta^3+3(\sigma_{Xi}^2\sigma_{Yi}^2\tilde{\beta}_{Xi}^2-\sigma_{Xi}^4\tilde{\beta}_{Yi}^2)\theta^2-6\sigma_{Xi}^2\sigma_{Yi}^2\tilde{\beta}_{Xi}\tilde{\beta}_{Yi}\theta+(\sigma_{Xi}^2\sigma_{Yi}^2\tilde{\beta}_{Yi}^2-\tilde{\beta}_{Xi}^2\sigma_{Yi}^4)}{(\sigma_{Xi}^2\theta^2+\sigma_{Yi}^2)^3}, \tag{8}$$

as $\tilde{\beta}_{Xi} \xrightarrow{p} \beta_{Xi}$, $\tilde{\beta}_{Yi} \xrightarrow{p} \beta_{Yi}$, $\hat{\theta} \xrightarrow{p} \theta_0$, we get $-\phi'(\hat{\theta})/V \xrightarrow{p} 1$, with Theorem 3.3 in [2], $-\phi'(\hat{\theta})/V|\mathscr{D} \xrightarrow{w.P} 1$.

Based on equation (8), we can see $\phi''(\theta)$ has its numerator of order $n^5$ and its denominator of order $n^6$, thus $\phi''(\theta^*)/V = O_p(1)$. As $\tilde{\theta} \xrightarrow{p} \theta_0$, $\hat{\theta} \xrightarrow{p} \theta_0$, we have $\tilde{\theta} - \hat{\theta} \xrightarrow{p} 0$, again with Theorem 3.3 in [2] we get $\tilde{\theta} - \hat{\theta}|\mathscr{D} \xrightarrow{w.P} 0$. Thus we can get $\frac{1}{2}\phi''(\theta^*)(\tilde{\theta} - \hat{\theta})|\mathscr{D} \xrightarrow{w.P} 0$. Now with Theorem 3.2 in [2], we can get $\sqrt{V}(\tilde{\theta} - \hat{\theta})|\mathscr{D} \xrightarrow{w.P} N(0,1)$, completeing the proof. □

# References


[1] Lyon, M. S., Andrews, S. J., Elsworth, B., Gaunt, T. R., Hemani, G., & Marcora, E. (2021). The variant call format provides efficient and robust storage of GWAS summary statistics. *Genome Biology*, 22(1), 1-10.

[2] Xiong, S., & Li, G. (2008). Some results on the convergence of conditional distributions. Statistics & probability letters, 78(18), 3249-3253.

[3] Xue, H., Shen, X., & Pan, W. (2021). Constrained maximum likelihood-based Mendelian randomization robust to both correlated and uncorrelated pleiotropic effects. The American Journal of Human Genetics, 108(7), 1251-1269.

[4] Zhao, Q., Wang, J., Hemani, G., Bowden, J., & Small, D. S. (2020). Statistical inference in two-sample summary-data Mendelian randomization using robust adjusted profile score. The Annals of Statistics, 48(3), 1742-1769.
